# Systematic Catalyst Variation for Improved Stereoselective Epoxide Polymerization: Subtle Modifications Resulting in Superior Efficiency

**DOI:** 10.1021/jacs.6c02471

**Published:** 2026-07-05

**Authors:** Bai-Hao Ren, Bryce M. Lipinski, Lilliana S. Morris, Anna C. Overholts, Judy Pan, Xiao-Bing Lu, Geoffrey W. Coates

**Affiliations:** † Department of Chemistry and Chemical Biology, Baker Laboratory, 5922 Cornell University, Ithaca, New York 14853-1301, United States; ‡ State Key Laboratory of Fine Chemicals, Frontiers Science Center for Smart Materials, 12399 Dalian University of Technology, Dalian 116024, China

## Abstract

Isotactic poly­(propylene oxide) (*i*PPO) is a semicrystalline polyether that has emerged as a high-strength, photodegradable material for marine applications. To improve the accessibility of *i*PPO, catalysts with higher activity and selectivity are required. Using rational catalyst design informed by computational insights, we developed a flexibly tethered, bimetallic chromium catalyst exhibiting high enantioselectivity (*k*_*rel*_ ∼ 100) and unprecedented activity (TOF ∼ 50,000 h^–1^) for propylene oxide (PO) polymerization. Mechanistic studies reveal that high enantioselectivity originates from increased steric bulk at the *ortho* position of the salicylimine moiety, which increases steric repulsion between the alkoxide chain end and the ligand in the disfavored transition state. Furthermore, introducing geminal dimethyl groups that rigidify the flexible tether between the two ligand moieties significantly enhances catalyst activity by destabilizing the resting state during polymerization. The catalyst remains active at loadings as low as 0.5 ppm, enabling the synthesis of colorless, tough *i*PPO.

## Graphical abstract

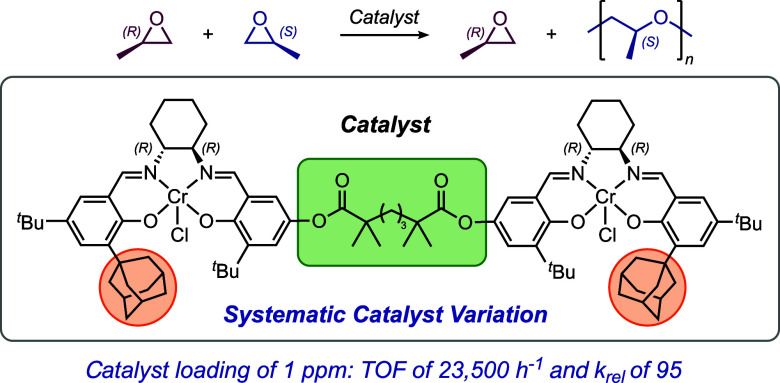

## Introduction

Over half the mass of the plastic in the Great Pacific Garbage Patch consists of fishing nets, ropes, and lines made from tough and persistent polyolefin and polyamide materials that are detrimental to aquatic life.^1–3^ In the search for tough yet degradable plastic alternatives for marine applications, isotactic poly­(propylene oxide) (*i*PPO) has attracted attention.^4^ This semicrystalline polyether is mechanically robust, exhibiting pronounced strain hardening with an ultimate tensile strength that is comparable to Nylon-6,6. Furthermore, its ethereal backbone is susceptible to oxidative degradation upon exposure to UVA light.^4^

The large-scale industrial synthesis of isotactic poly­(propylene oxide) (*i*PPO) remains challenging,^5–8^ because current production still relies on costly enantiopure propylene oxide (PO)^9–13^ and only a few catalysts have demonstrated effective stereocontrol.^14–19^ Because the crystallinity and mechanical properties of *i*PPO depend strongly on tacticity,^4^ chemists have focused on developing highly active and stereoselective polymerization catalysts for *rac*-PO, an abundant feedstock.^20^

In 2008, our group reported a rigid binaphthol-linked bimetallic (salen)­Co­(III) complex capable of enantioselectively polymerizing PO, with a relative rate constant (*k*_rel_ = *k*_fast_/*k*_slow_) > 300, to give highly isotactic PPO with [*mm*] >98% ([*mm*] is the percent of triads with *meso* configurations across the ether bonds).^21–23^ In 2017, we reported a related bimetallic (salalen)­Cr­(III) catalyst offering enhanced molecular weight control and tolerance to chain shuttling agents while maintaining high isoselectivity.^24^ Despite these advances, developing an isoselective PO polymerization catalyst suitable for the large-scale production of *i*PPO remains elusive.

The ideal catalyst for large-scale *i*PPO production would: (1) demonstrate high isoselectivity because the tacticity of *i*PPO greatly influences its mechanical properties, and (2) exhibit high activity at low catalyst loadings. Lower loadings reduce production costs through lower catalyst consumption and minimize polymer purification to remove colored, potentially toxic catalyst residues. Guided by Jacobsen’s seminal work on bimetallic cooperative asymmetric epoxide ring-opening,^25–28^ our group reported a flexibly tethered, bimetallic (salcy)Cr catalyst that produced *i*PPO with a narrow dispersity. Optimization identified a six-methylene linker as optimal, demonstrating moderate activity (TOF = 627 h^–1^) and enantioselectivity (*k*_rel_ = 59), producing *i*PPO with a [*mm*] = 88% at 125 ppm (μmol/mol) catalyst loading (Figure 1).^29,30^

**Figure 1:**
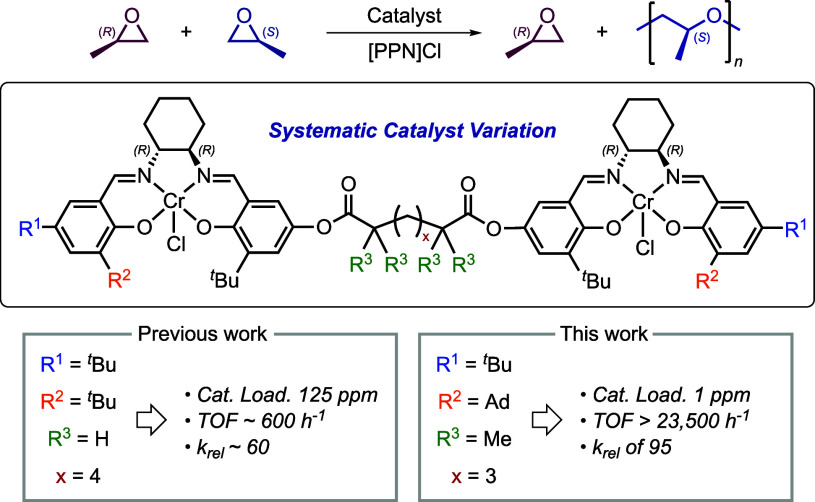
Tethered bimetallic catalysts for *i*PPO synthesis.

To further optimize the flexibly tethered, bimetallic (salcy)­Cr catalyst for improved enantioselectivity and activity, we report here a joint experimental and density functional theory (DFT) computational approach. We systematically varied the ligand framework to elucidate the connection between steric and electronic modifications and their impact on enantioselectivity and catalyst activity. Then, DFT computations further provided mechanistic insights into the observed trends. Optimization efforts led to the development of a catalyst with exceptionally high activity (TOF > 23,500 h^–1^) and enhanced enantioselectivity (*k*_rel_ = 95) at low catalyst loadings (1 ppm).

## Results and Discussion

### Experimentally Guided Ligand Modification

The flexibly tethered salcy ligand framework offers three main opportunities for catalyst optimization: variation of the *para-* (R^1^) and *ortho-* (R^2^) substituents on the salicylimine moiety and the identity of the flexible tether (R^3^ and *x*). We first investigated the steric and electronic effects of the *para*-position (R^1^) of the salcy framework. As R^1^ is distant from the Cr centers and has little influence on the catalytic pocket, changing R^1^ from ^*t*^Bu to smaller methyl or a bulky trityl gave similar activities (TOF = 643, 757, and 467 h^–1^ respectively, Table 1, entries 1–3). Conversely, changing the electronics of R^1^ influenced activity. The presence of electron-withdrawing groups, such as Cl and F, yielded higher activity (TOF = 1070 and 989 h^–1^, Table 1, entries 4 and 5), likely due to the enhanced Lewis acidity of the Cr centers.^31^ In contrast, an electron-donating OMe group resulted in lower catalyst activity (TOF = 595 h^–1^, Table 1, entry 6). However, all R^1^ modifications reduced enantioselectivity (entries 2–6) compared to the parent R^1^ = ^*t*^Bu catalyst. Balancing activity and enantioselectivity considerations, R^1^ = ^*t*^Bu was selected for subsequent catalyst optimization.

**Table 1:**
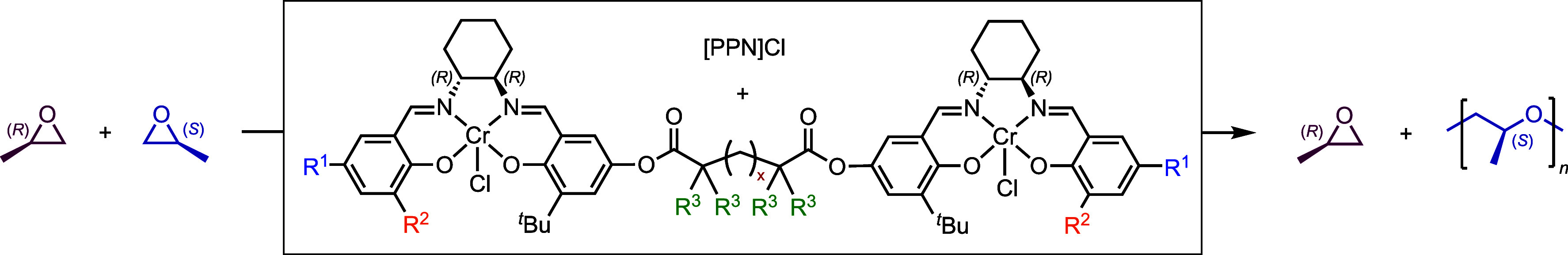
Preliminary Results of Catalyst Optimization^a^

entry (catalyst)	R^1^	R^2^	R^3^	*x*	time (h)	conv. (%)^b^	TOF (h^–1^)^c^	*M*_n_ (kDa)^d^	*Đ* ^d^	[*mm*] (%)^e^	**k*_rel_ ^f^*
1	^*t*^Bu	^*t*^Bu	H	4	6.0	49	643	41.2	1.41	87	63
2	Me	^*t*^Bu	H	4	5.0	47	757	51.3	1.41	86	48
3	CPh_3_	^*t*^Bu	H	4	8.0	47	467	44.9	1.48	85	46
4	Cl	^*t*^Bu	H	4	3.5	48	1070	45.6	1.41	81	33
5	F	^*t*^Bu	H	4	4.0	50	989	44.3	1.40	81	37
6	OMe	^*t*^Bu	H	4	6.0	45	595	41.9	1.35	87	47
7	^*t*^Bu	Me	H	4	9.0	38	334	23.1	1.19	79	16
8	^*t*^Bu	Ad	H	4	4.0	49	982	58.7	1.33	91	117
9	^*t*^Bu	CPh_3_	H	4	24.0	<1	n.a.^g^	n.a.^g^	n.a.^g^	n.a.^g^	n.a.^g^
10	^*t*^Bu	^*t*^Bu	Me	4	1.5	50	2590	41.6	1.46	85	53
11	^*t*^Bu	^*t*^Bu	Me	5	2.0	49	1970	44.1	1.45	86	58
12	^*t*^Bu	^*t*^Bu	Me	3	0.7	50	6150	46.2	1.48	83	42
13	^*t*^Bu	Ad	Me	3	0.8	48	5000	68.7	1.48	92	104

a Polymerization conditions: [PO]:[catalyst]:[PPNCl] = 8000:1:1, [PO] = 4.8 M in dimethoxyethane (DME), temperature = 23 °C.

b Determined gravimetrically.

c mmol PO consumed × mmol catalyst^–1^ × time^–1^.

d Determined by GPC in THF, calibrated with polystyrene standards.

e Determined by ^13^C NMR spectroscopic analysis.

f Calculated based on tacticity and conversion, for more details see Supporting Information.

g n.a. = not applicable.

We next probed the influence of steric bulk at the *ortho*-position (R^2^) of the salicylimine. As R^2^ is closer to the Cr metal centers, we anticipated substantial effects of altering R^2^ on activity and stereoselectivity. As expected, increasing the steric bulk of R^2^ led to an increase in enantioselectivity with Me < ^*t*^Bu < Ad (*k*_rel_ = 16, 63, and 117, respectively, Table 1, entries 7, 1, and 8).^32,33^ However, further increasing the steric bulk to a trityl group resulted in a complete loss of activity (Table 1, entry 9), which we attribute to steric repulsion restricting the cooperative epoxide ring-opening between the two salcy Cr units.

We next examined how the tether identity (R^3^ and *x*) influences activity and selectivity, because we hypothesized that the tether would greatly impact how the metal centers work together during polymerization.^29,34^ Previous mechanistic studies have shown that in these systems, one metal acts as a Lewis acid to activate the epoxide while the other metal delivers the nucleophile within a single chiral pocket, thereby enhancing activity and enantioselectivity.^27^ We hypothesized that adding geminal dimethyl groups to the linker would help impose the necessary bimetallic geometry through the Thorpe-Ingold effect.^35–37^ Gratifyingly, adding geminal dimethyl groups at the ends of the tether (R^3^ = Me) resulted in a 4-fold increase in activity (*x* = 4, TOF = 643 vs 2590 h^–1^, Table 1, entry 1 vs 10). Altering the number of methylene units between the geminal dimethyl groups also influenced the resulting activity. Increasing the tether length to *x* = 5 resulted in a slight decrease in activity (TOF = 1970 h^–1^, Table 1, entry 11), while decreasing the tether length to *x* = 3 led to an increase to a TOF of 6150 h^–1^, almost anchain-end order of magnitude higher than the original catalyst. This suggests the distance between the salcy frameworks is integral to catalyst efficiency. Interestingly, adding geminal dimethyl groups to the flexible linker had little influence on the resulting enantioselectivity (Table 1, entries 1 and 10–12).

Finally, with these catalyst optimization trends established, we aimed to design a catalyst with high selectivity and activity. By combining our optimized catalyst substituents (Table 1, entry 13), we created a highly active (TOF = 5000 h^–1^) and enantioselective (*k*_rel_ = 104) catalyst for propylene oxide (also active and selective for 1-butene oxide and 1-hexene oxide, Table S2) for further investigations (*vide infra*).

### DFT Mechanistic Investigation

To gain a deeper understanding of experimental observations, we conducted a DFT-based mechanistic investigation. Our calculations first established the catalyst geometry, using 1-methoxypropan-2-olate as a simplified model for the propagating chain end. The geometry of tethered catalysts of this nature is described in the work by Jacobsen and co-workers as “Head to Tail” (**H–T**) or “Head to Head” (**H–H**, Figure 2A) depending on whether the cyclohexyldiamine moieties face opposite or the same directions, respectively.^25^ We calculated the relative energy of transition states for (*R*)/(*S*)-PO ring-opening in **H–T** and **H–H** conformations mediated by the (*R,R,R,R*)-catalyst **1** (Figure 2B). The computational results indicate that the **H–H** conformation is preferred in both (*R*)- and (*S*)-PO ring-opening, as it better accommodates the steric bulk of the growing polymer chain-end (for more details, see Figure S1).

**Figure 2:**
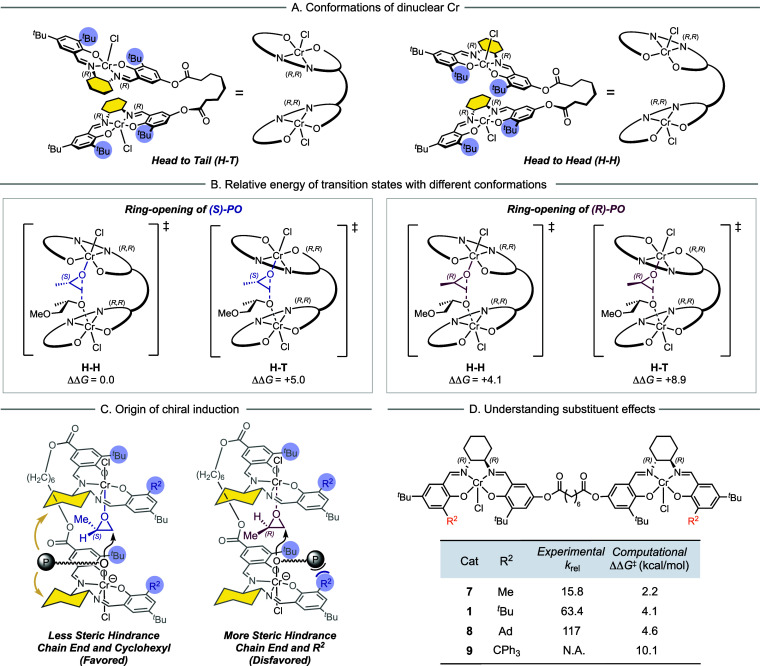
Computational investigation of chiral induction with catalyst **1** as the model: (A) Conformations of dinuclear Cr­(III) complex; (B) relative energy (kcal/mol) of transition states with different conformations; (C) illustration of chiral induction; and (D) understanding substituent effects.

Experimentally, we observed that the (*R,R,R,R*) catalyst generates (*S*)-*i*PPO leaving unreacted enantioenriched (*R*)-PO. DFT calculations show that the (*S*)-PO ring-opening transition state is 4.1 kcal/mol lower in Gibbs free energy than the (*R*)-PO analogue (see Figure S2). Intriguingly, in the (*S*)-PO ring-opening transition state, the polymer chain end lies near the cyclohexyldiamine groups, experiencing less steric hindrance than the chain end in the (*R*)-PO ring-opening transition state, where it faces toward the R^2^ substituents (for more detailed 3D structures, see Figure S3).

The chain end orientation change during (*R*) vs (*S*)-PO ring-opening also explains the experimental observation that increasing the steric bulk of R^2^ results in an increase in enantioselectivity. A smaller R^2^ provides less steric hindrance, reducing the ΔΔ*G* ^‡^ between the (*R*)/(*S*)-PO ring-opening transition states. DFT calculations agree that R^2^ = Me (ΔΔ*G* ^‡^ = 2.2 kcal/mol, *k*_rel_ = 16) is less selective than R^2^ = ^*t*^Bu (ΔΔ*G* ^‡^ = 4.1 kcal/mol, *k*_rel_ = 63) which is less selective than R^2^ = Ad (ΔΔ*G* ^‡^ = 4.6 kcal/mol, *k*_rel_ = 117, Figure 2D). However, even though a theoretically superior enantioselectivity was obtained by trityl (ΔΔ*G* ^‡^ = 10.1 kcal/mol, catalyst **9**), no polymerization occurred under the standard conditions, indicating that excessive R^2^ steric hindrance can inhibit catalysis entirely.

We also wanted to clarify how the flexible linker influences catalyst activity. Specifically, we were interested in understanding why the addition of the geminal dimethyl substituents and shortening the tether drastically increases catalyst activity. To elucidate the rationale for these experimental observations, we conducted a computational exploration of the reaction coordinate (Figure S4). Initially, the metal-alkoxide resting-state (**AA**) undergoes ligand exchange via Cr–O (ether-type) dissociation, followed by subsequent PO coordination (**R**). Then, as the rate-determining step, the nucleophilic alkoxide chain end attacks the activated PO.

After analyzing key geometric parameters along the reaction coordinate (for all the parameters we analyzed, see Table S1), we found that as the distance between the two cyclohexyldiamine moieties (*D*_cycl_) in **AA** decreased, the catalyst activity increased (a linear inverse correlation, Figure S5). The geminal dimethyl groups (R^3^ = Me) influence activity by pushing the two salcy frameworks of **AA** together, which destabilizes the resting state, increasing activity. This effect is amplified with shorter methylene linkers, explaining the superior activity of catalyst **12** compared to **10** and **11**. In agreement with experimental data, the most active catalyst (catalyst **12**) possesses the shortest *D*_cycl_(**AA**) of 5.27 Å (Table 2, entry 5).

**Table 2:**
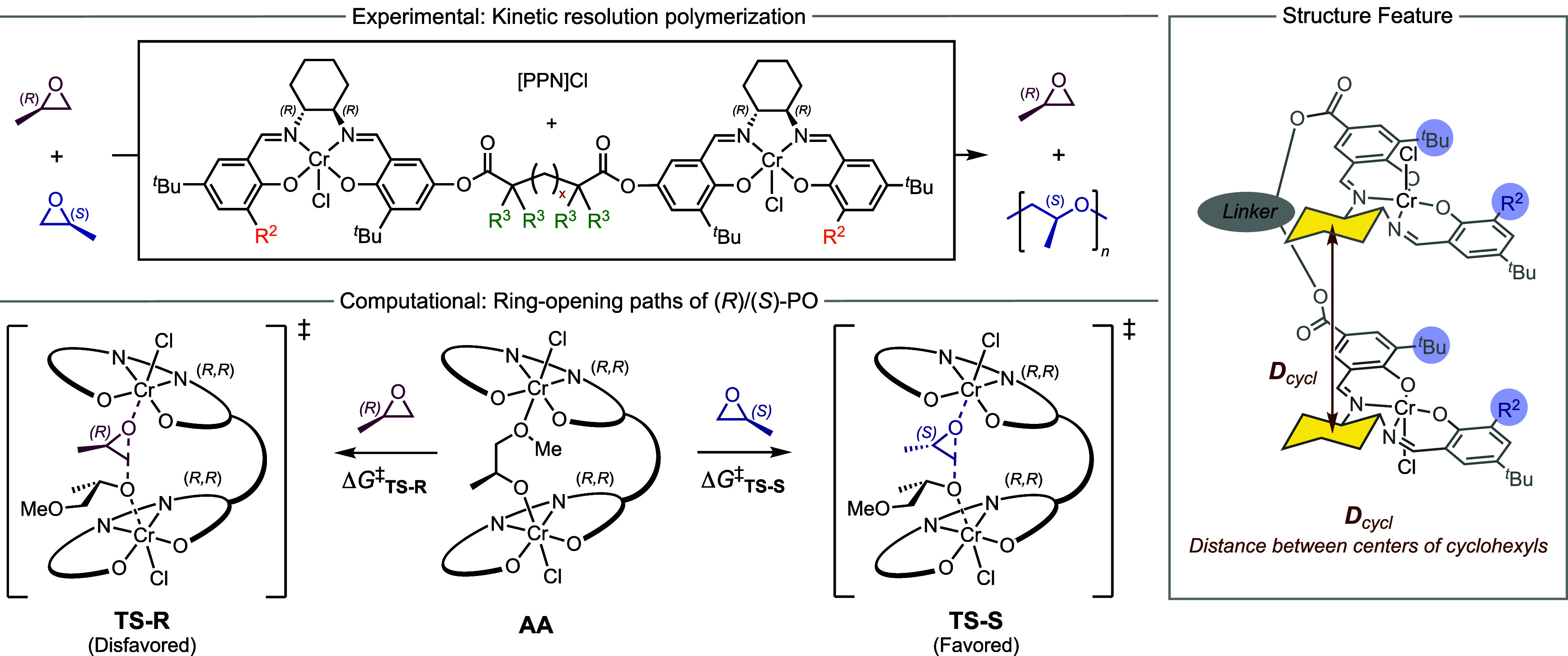
Computational Results for Understanding the Role of the Linker

					*D*_cycl_ ^a^					
entry	catalyst	R^2^	R^3^	*n*	**AA**	**TS-S**	**TS-R**	Δ*G* ^‡^_**TS‑S**_ ^b^	Δ*G* ^‡^_**TS‑R**_ ^b^	ΔΔ*G* ^‡^ ^b^
1	**1**	^*t*^Bu	H	4	7.25	7.79	5.59	18.9	23.1	4.1
2	**8**	Ad	H	4	7.53	7.86	5.85	18.1	22.7	4.6
3	**10**	^*t*^Bu	Me	4	5.85	7.79	5.88	16.5	20.0	3.5
4	**11**	^*t*^Bu	Me	5	6.64	7.90	5.89	16.7	20.3	3.6
5	**12**	^*t*^Bu	Me	3	5.27	7.68	5.61	14.7	18.1	3.4
6	**13**	Ad	Me	3	5.45	7.58	5.72	15.4	20.3	4.9

a *D*_cycl_ is the distance between centers of cyclohexyls, given in Å.

b Differences of Gibbs free energy were given in kcal/mol; Δ*G* ^‡^_**TS‑S**_ and Δ*G* ^‡^_**TS‑R**_ are calculated relative to **AA**; predicted stereoselectivity (ΔΔ*G* ^‡^) = Δ*G* ^‡^_**TS‑R**_ – Δ*G* ^‡^_**TS‑S**_.

Moreover, we also found that geminal dimethyl had little influence on the structures of transition states: all *D*_cycl_(**TS-S**)­s are about 7.70 Å and *D*_cycl_(**TS-R**)­s are roughly 5.60 Å (Table 2). This indicated that the linker has a minimal influence on enantioselectivity, which is consistent with both computational results (ΔΔ*G* ^‡^_**stereo**_ ∼ 4.0 kcal/mol for R^2^ = ^*t*^Bu (catalysts **1** and **10**–**12**) and ΔΔ*G* ^‡^_**stereo**_ ∼ 4.8 kcal/mol for R^2^ = Ad (catalysts **8** and **13**, Table 2), and experimental observations (Table 1). Therefore, catalyst **13**, which combines the advantages of R^2^ and the tether, was identified as the most effective catalyst in terms of both enantioselectivity and activity.

### Application of the Optimized Catalyst

Using the optimized catalyst **13**, we examined its industrial potential, focusing on performance at low catalyst loadings. We first varied catalyst loading. At 10 ppm (μmol/mol) catalyst loading in neat PO (Table 3, entry 1), conversion reached 49% in 0.9 h (TOF > 50,000 h^–1^), while maintaining excellent enantioselectivity (*k*_rel_ = 96). We also observed a broader dispersity (*Đ* = 2.50) which was attributed to high viscosity affecting mass transfer and adventitious water causing chain termination. As catalyst loading was further lowered to 5, 1, and 0.5 ppm (Table 3, entries 2–4), activity (TOF = 35,200, 23,500, and 20,200 h^–1^) and enantioselectivity dropped slightly (*k*_rel_ = 95, 95, and 80).

**Table 3:**
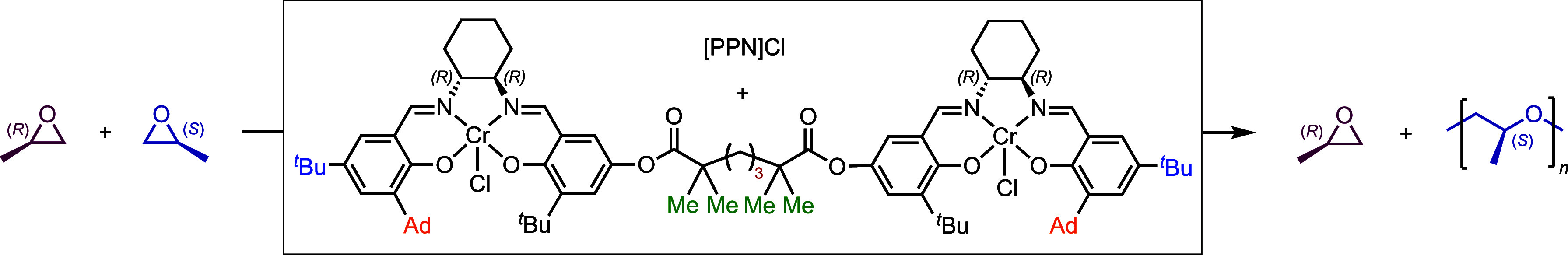
Catalyst Loading Screen^a^

entry	[Cat]/[PO]	Time (h)	Conv. (%)^b^	TOF (h^–1^)^c^	*M*_n_ (kDa)^d^	*Đ* ^d^	[*mm*] (%)^e^	*k*_rel_ ^f^
1	10 ppm	0.9	49	53,900	149	2.50	92	96
2	5 ppm	1.5	26	35,200	182	1.86	96	95
3	1 ppm	15	35	23,500	246	1.95	95	95
4	0.5 ppm	39	39	20,200	296	2.12	94	80

a Polymerization conditions: Polymerization in neat PO; [Cat]/[PPNCl] = 1/1; Temperature = 23 °C.

b Determined gravimetrically.

c mmol PO consumed × mmol catalyst^–1^ × time^–1^.

d Determined by GPC in THF, calibrated with polystyrene standards.

e Determined by ^13^C NMR spectroscopic analysis.

f Calculated based on tacticity and conversion, for more details see Supporting Information.

Overall, the catalyst displayed unprecedented activity and enantioselectivity even at 0.5 ppm catalyst loading. High activity significantly reduces residual metal content in the final polymer, yielding a lighter-colored product with potentially lower toxicity. *i*PPO synthesized with a catalyst loading of 0.5 ppm (Table 3, entry 4) was nearly colorless (Figure S6). Furthermore, we investigated the mechanical properties of *i*PPO with *M*_n_ of 296 kDa (Table 3, entry 4). The polymer has an ultimate tensile strength of 81 MPa and exhibited the serrated response (Figure S7). This result is consistent with our previous report and comparable to that of Nylon-6,6.^4^

## Conclusions

In summary, we conducted a systematic catalyst optimization study to design a catalyst with excellent activity and high enantioselectivity. DFT calculations were used to rationalize observed trends. Enantioselectivity is primarily governed by steric effects from the *ortho*-position (R^2^) of the salicylimine moiety. Because R^2^ lies near the polymer chain end in the disfavored transition state, larger R^2^ substituents lead to a higher ΔΔ*G* ^‡^_**stereo**_, thus higher enantioselectivity.

The flexible linker strongly influences catalyst activity. Linkers with terminal geminal dimethyl groups compress the salen framework, destabilizing the resting state (**AA**) without significantly changing the transition state (**TS**), which increases activity. Combining steric bulk at R^2^ = Ad and adding geminal dimethyl groups on the linker, a fully optimized catalyst **13** shows exceptional enantioselectivity (*k*_rel_ > 100) and superior activity (TOF > 50,000 h^–1^). Remarkably, even at a catalyst loading as low as 0.5 ppm (13 ppm by mass), the catalyst retains its high efficiency (*k*_rel_ = 80, TOF = 20,200 h^–1^), producing an almost colorless polymer with a tensile strength of 81 MPa. These results set a new benchmark for stereoselective PO polymerization and highlight the industrial potential of such catalysts, offering a practical pathway toward scalable, sustainable production of high-performance *i*PPO materials.

## Supplementary Material



## Notes

General information, supplementary computational discussion, more details of complex synthesis, procedures of polymerization and characterization of polymers (NMR spectra and GPC curve) (PDF) and Cartesian coordinates of the calculation structures mentioned in the text (*xyz*) (PDF)
