# Supplementary material for: Systematic Catalyst Variation for Improved Stereoselective Epoxide Polymerization: Subtle Modifications Resulting in Superior Efficiency
Source: J Am Chem Soc. 2026 Jul 5;148(27):28276–82. doi: 10.1021/jacs.6c02471 (PMC13383729; doi:10.1021/jacs.6c02471)
Supplement: Supplementary file 1 [file ja6c02471_si_001.pdf]

Supporting Information for

Systematic Catalyst Variation for Improved Stereoselective Epoxide Polymerization: Subtle Modifications Resulting in Superior Efficiency

Bai-Hao Ren,[†] Bryce M. Lipinski,[†] Lilliana S. Morris,[†] Anna C. Overholts,[†] Judy Pan,[†] Xiao-Bing Lu[‡] and Geoffrey W. Coates[†]*

[†]Department of Chemistry and Chemical Biology, Baker Laboratory, Cornell University, Ithaca, New York 14853-1301, United States

[‡]State Key Laboratory of Fine Chemicals, Frontiers Science Center for Smart Materials, Dalian University of Technology, Dalian 116024, China

Email: coates@cornell.edu

Table of Contents

1. General Information S2

2. Tacticity Calculations S4

3. More details for DFT S7

4. Polymer Properties S13

5. Polymerization of Other Epoxides S15

6. Polymerization Characterization Data S17

7. Synthetic Procedures S53

7.1 Diacids and Diacid Chlorides S53

7.2 Phenols S55

7.3 Salicylaldehydes S56

7.4 Salen Moieties S58

7.5 Ligands S62

7.6 Catalysts S67

8. ^{1}H and ^{13}C NMR Spectra S69

9. Reference S89

1. General Information

All manipulations of air- and/or water-sensitive compounds were carried out under dry nitrogen using an MBraun Labmaster glovebox or standard Schlenk line techniques. NMR spectra were recorded on a 500 MHz Bruker AV III HD spectrometer with broadband Prodigy Cryoprobe (^{1}H, 500 MHz). ^{1}H NMR spectra were referenced with residual solvent shifts ($CHCl_3$ = 7.26 ppm). ^{13}C NMR spectra were referenced by solvent shifts ($CDCl_3$ = 77.16 ppm). High-resolution mass spectrometry (HRMS) analyses were performed on a Thermo Scientific Exactive Orbitrap MS system equipped with an Ion Sense DART ion source. Gel permeation chromatography (GPC) was performed using an Agilent 1260 Infinity system, equipped with UV and refractive index detectors, and two Agilent PolyPore columns (5 micron, 4.6 mm ID). The GPC columns were eluted with THF at 30 °C at 0.3 mL/min and were calibrated with polystyrene standards.

Polymerization

General PO Polymerization Procedure: In a nitrogen filled glovebox, catalyst (0.0018 mmol, 1 equiv.) and [PPN]Cl (1.0 mg, 0.0018 mmol, 1 equiv.) were added to a 20 mL septa vial equipped with a stir bar and 2 mL DME. PO (835 mg, 14.4 mmol, 8000 equiv.) was added by mass difference using a 1 mL plastic syringe to start each reaction. Reactions were quenched by rapid removal of volatiles by reduced pressure. Conversion was determined gravimetrically while accounting for residual catalyst and [PPN]Cl.

Density Functional Theory Calculations

DFT calculations were performed as follows unless otherwise specified: All calculations were performed using the Gaussian 16 suite of programs. The PBE0-D3(BJ) DFT method was used for single-point energy calculations, geometry optimizations, and frequency analyses. The 6-31G(d) electron basis set was used for all atoms. Frequency calculations were performed to identify the geometrically-optimized stationary points (no imaginary frequencies for minima and one imaginary frequency for transition-state structures) and obtain thermodynamic data. To improve the energy accuracy, the single-point energy of each optimized structure was calculated using 6-311+G(d,p) for all atoms and the solvation model based on density (SMD) with tetrahydrofuran as the solvent to simulate the solvent environments of epoxides. The relative free energy was obtained by combining the single-point energy with the Gibbs free energy correction. In (salen)Cr complexes, Cr tends to adopt a high-spin state, and therefore, all the binuclear Cr complexes were computed in the septet state here.[1]

Materials

HPLC grade methylene chloride, methanol (MeOH), tetrahydrofuran (THF), toluene, and hexane were purchased from Fisher Scientific, purified over solvent columns, and degassed by three freeze-pump-thaw cycles. Deuterated solvents were purchased from Cambridge Isotope Laboratory. 3 Å molecular sieves, purchased from Strem, were activated by heating at 200 °C under vacuum for 18 hours. Dimethoxyethane (DME) was purchased from Sigma-Aldrich and dried over Na/benzophenone for at least three days. DME was then vacuum transferred, degassed by three freeze-pump-thaw cycles, and stored over 3 Å molecular sieves. Propylene oxide (PO) was purchased from Sigma-Aldrich, dried over calcium hydride for three days, and subsequently treated with n-butyllithium at –78 °C for 2 h before being vacuum transferred to a Straus storage flask. PO was degassed by three freeze-pump-thaw cycles. Bis(triphenylphosphine)iminium chloride ([PPN]Cl), purchased from Sigma-Aldrich, was recrystallized at room temperature over 5 days from anhydrous methylene chloride/hexane and dried at 80 °C under vacuum overnight.[2] Triethylamine was purchased from Sigma-Aldrich and degassed before use. Chromium(II) chloride was purchased from Strem Chemicals and stored under a nitrogen atmosphere. The ligand component (*R*,*R*)-1,2-diaminocyclohexane was purchased from Combi-Blocks and used as received to form the mono-HCl salt as previously reported.[3] All other reagents were purchased from commercial sources and used as received.

Tensile Testing Procedure

The tensile testing procedure was slightly modified based on our previous report.[2]

Sheets of *i*PPO for tensile testing were prepared using a 4.0 × 4.0 × 0.6 cm stainless-steel square mold on a Carver 4120 hydraulic hot press at 90 °C for 10 min. After pressing, the mold was removed from the press and placed between two metal plates to accelerate heat dissipation. The resulting sheets were then die-cut using an ISO 37-4 die cutter, affording 6 tensile specimens per sample. Uniaxial tensile elongation measurements were performed on a Shimadzu Autograph AGS-X tensile tester under ambient conditions at a crosshead speed of 10 mm min^{-1} until failure.

2. Tacticity Calculations

Quantifying Polymer Isotacticity: Although there is some overlap of the *mr* and *rm* peaks, the polyethers synthesized in this paper exhibit triad resolution of the methine carbon.[4] Due to this overlap, [*mr*] and [*rm*] were determined from [*rr*]. The *mm* (δ = 75.62 ppm), clearly visible ^{13}C satellite upfield of *mm, mr* + *rm* (δ = 75.46, 75.42 ppm), and *rr* (δ = 75.22 ppm) peaks were integrated separately. The selectivity factor (k_{rel}) for each polymerization was calculated using S7 and S8, where *c* is the conversion.

$$mm_{total} = \int mm + 2 \times \int mm_{satellite} \qquad \text{(S1)}$$

$$(mr + rm)_{total} = \int mr + \int rm - \int mm_{satellite} \qquad \text{(S2)}$$

$$rr_{total} = \int rr \qquad \text{(S3)}$$

$$[mm] = \frac{mm_{total}}{mm_{total} + (mr + rm)_{total} + rr_{total}} \qquad \text{(S4)}$$

$$([mr] + [rm]) = \frac{(mr + rm)_{total}}{mm_{total} + (mr + rm)_{total} + rr_{total}} \qquad \text{(S5)}$$

$$[rr] = \frac{rr_{total}}{mm_{total} + (mr + rm)_{total} + rr_{total}} \qquad \text{(S6)}$$

$$ee_{(p)} = (2[mm] + [mr] + [rm] - 1)^{1/2} \qquad \text{(S7)}$$

The calculation of k_{rel}:

The concentrations of the *S*- and *R*-enantiomers of PO remaining during the reaction are denoted as [*S*] and [*R*], respectively. The enantiomeric excess of the unreacted PO, ee_{PO} is defined as:

$$ee_{\mathrm{PO}} = \frac{[S] - [R]}{[S] + [R]}$$

In addition, if *c* is the conversion of PO, the total concentration of unreacted PO is given by

$$[\mathrm{PO}] \times (1 - c) = [S] + [R]$$

wherein $[\mathrm{PO}]_0$ is the initial concentration of PO.

Therefore, combining the above two equations gives

$$[S] = \frac{(1-ee_{\mathrm{PO}})(1-c)}{2} \times [\mathrm{PO}]_0 \;\; and \;\; [R] = \frac{(1+ee_{\mathrm{PO}})(1-c)}{2} \times [\mathrm{PO}]_0$$

Since the consumption of each enantiomer follows first-order kinetics, one obtains

$$\frac{d[S]}{dt} = -k_S[S] \;\; and \;\; \frac{d[R]}{dt} = -k_R[R]$$

We could get

$$k_S = \frac{\ln[S]_o - \ln[S]}{t} = \frac{\ln(0.5[\mathrm{PO}]_0) - \ln[S]}{t} \;\; and \;\; k_R = \frac{\ln(0.5[\mathrm{PO}]_0) - \ln[R]}{t}$$

Therefore,

$$k_{rel} = \frac{k_S}{k_R} = \frac{\ln(0.5[\mathrm{PO}]_0) - \ln[S]}{\ln(0.5[\mathrm{PO}]_0) - \ln[R]} = \frac{\ln(0.5[\mathrm{PO}]_0) - \ln\frac{(1-ee_{PO})(1-c)}{2} \times [\mathrm{PO}]_0}{\ln(0.5[\mathrm{PO}]_0) - \ln\frac{(1+ee_{PO})(1-c)}{2} \times [\mathrm{PO}]_0} = \frac{\ln[(1-ee_{\mathrm{PO}})(1-c)]}{\ln[(1+ee_{\mathrm{PO}})\,(1-c)]}$$

Moreover, the enantiomeric excess of the product, $ee_{(\mathrm{p})}$, is related to the enantiomeric excess of the remaining substrate through mass balance:

$$ee_{(p)} = ee_{\mathrm{PO}} \times \frac{1-c}{c}$$

Therefore,

$$k_{rel} = \frac{k_S}{k_R} = \frac{\ln[(1-ee_{\mathrm{PO}})(1-c)]}{\ln[(1+ee_{\mathrm{PO}})\,(1-c)]} = \frac{\ln[1-c(1+ee_{(p)})]}{\ln[1-c(1-ee_{(p)})]} \quad \text{(S8)}$$

Example of calculation for Table 1, entry 1:

$$mm_{total} = 806.58 + 2 \times 25.61 = 857.80$$

$$(mr + rm)_{total} = \int mr + \int rm - \int mm_{satellite} = 110.86 - 26.61 = 84.25$$

$$rr_{total} = \int rr = 41.19$$

$$[mm] = \frac{mm_{total}}{mm_{total} + (mr+rm)_{total} + rr_{total}} = \frac{857.80}{857.80 + 84.25 + 41.19} = 0.8724$$

$$([mr]+[rm]) = \frac{(mr+rm)_{total}}{mm_{total}+(mr+rm)_{total}+rr_{total}} = \frac{84.25}{857.80+84.25+41.19} = 0.0857$$

$$[rr] = \frac{rr_{total}}{mm_{total}+(mr+rm)_{total}+rr_{total}} = \frac{41.19}{857.80+84.25+41.19} = 0.0419$$

$$ee_{(p)} = (2[mm]+[mr]+[rm]-1)^{1/2} = (2\times 0.8724+0.0857-1)^{1/2} = 0.911$$

$$k_{rel} = \frac{\ln[1-c(1+ee_{(p)})]}{\ln[1-c(1-ee_{(p)})]} = \frac{\ln[1-0.492\times(1+0.911)]}{\ln[1-0.492\times(1-0.911)]} = 63$$

3. More details for DFT

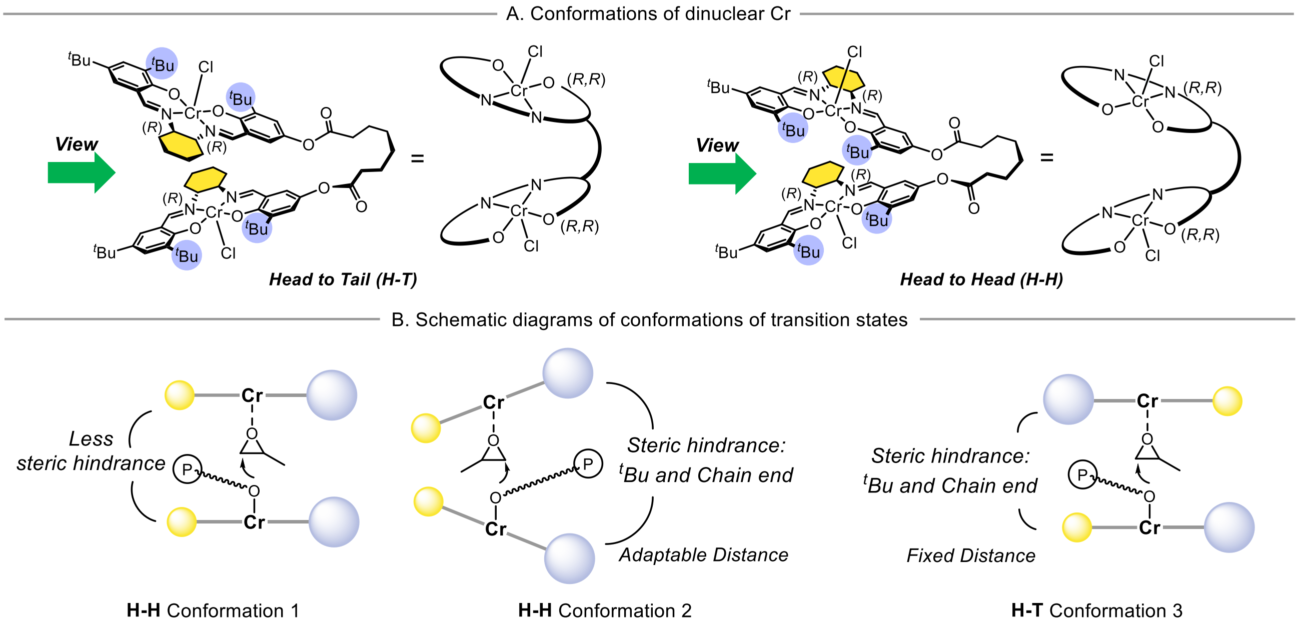

Figure S1. Understanding the conformations of transition states.

More discussion: Due to the structural characteristics of the transition states, the distance between the two Cr centers is constrained. However, the **H-H** conformation is more flexible in accommodating steric hindrance than the **H-T** conformation. When comparing conformations **1** and **2**, conformation **1** is more stable because of the reduced steric repulsion between the chain end and the cyclohexyl group.

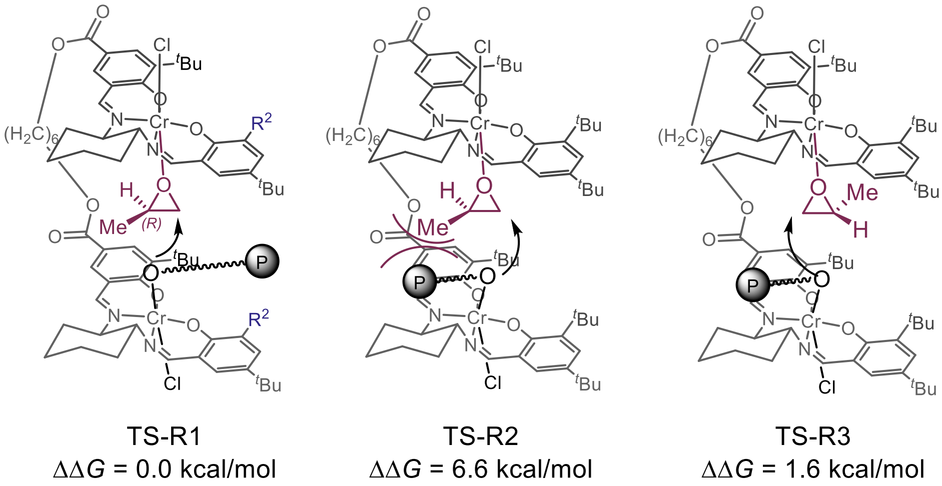

Figure S2. Representative conformations of transition states of (R)-PO ring-opening mediated by (*R*,*R*,*R*,*R*)-catalyst **1**. Gibbs free energy are related to **TS-R1**, given in kcal/mol.

More discussion: **TS-R1** is determined to be the most stable one. **TS-R2** is optimized by rotation of chain end, bringing it closer to the cyclohexyl group. The higher energy of **TS-R2** relative to **TS-R1** arises from steric repulsion between PO and the chain end.**TS-R3** is optimized by rotation of PO based on **TS-R2**, moving it away from the chain end. The increase in energy for **TS-R3** originates from steric interactions between PO and the salen framework.

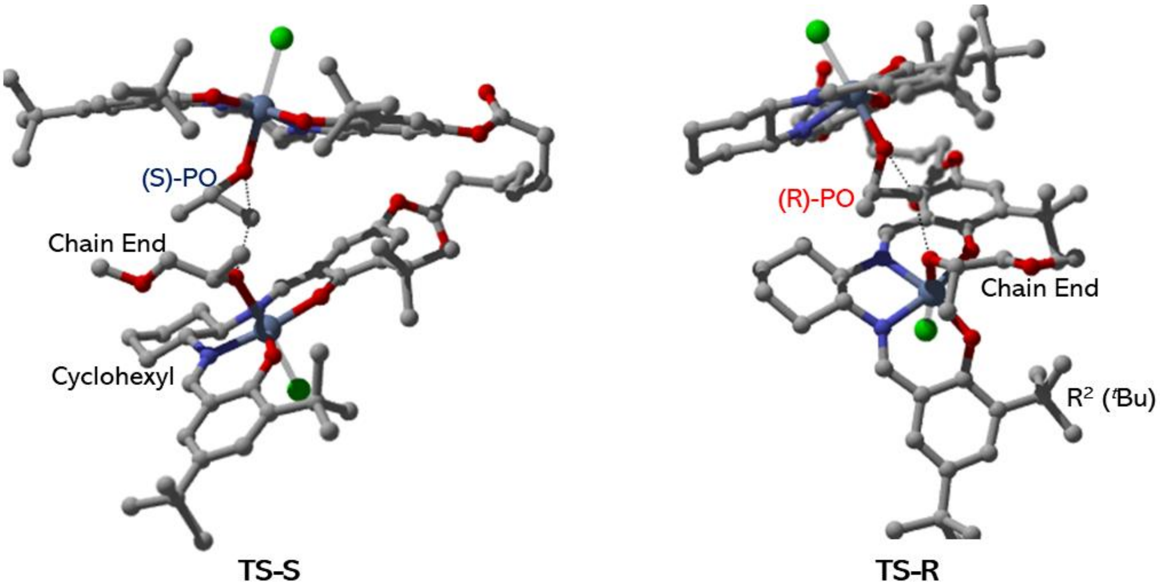

Figure S3. 3D structures of transition states of PO ring-opening mediated by (*R*,*R*,*R*,*R*)-catalyst **1.** Left: (*S*)-PO ring-opening (favored transition state); Right: (*R*)-PO ring-opening (unfavored transition state).

More discussion: Chain end in **TS-S** is close to cyclohexyl; chain end in **TS-R** is close to R^2 (^{t}Bu).

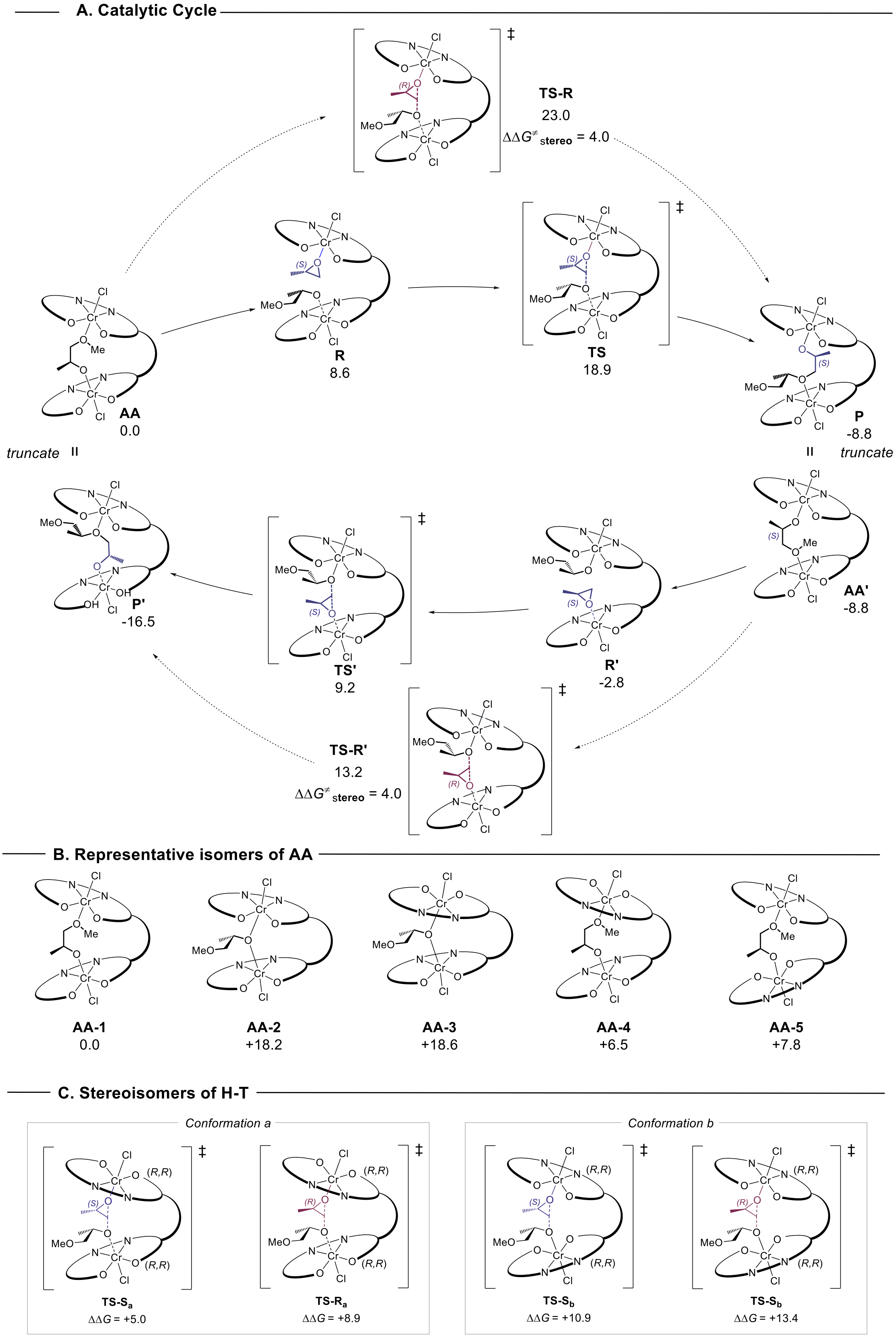

Figure S4. (A) Catalytic cycle of chain growth with two PO insertion, (B) representative isomers of **AA** and (C) stereoisomers of **H-T** transition states.

More discussion: Since the **H-H** conformation of the bimetallic Cr catalyst is preferred, the two Cr centers are theoretically nonequivalent. Therefore, we investigated chain growth involving two successive PO insertions. In the first insertion, one Cr center functions as a Lewis acid to activate the PO, while the other one coordinates the chain end. In the second insertion, the roles of the two Cr centers are interchanged. The results indicate that the two chain-growth pathways are very similar (ΔG = 18.9 and 18.0 kcal/mol, and $\Delta\Delta G^{\ddagger}_{\mathbf{stereo}}$ = 4.0 kcal/mol for both). Consequently, only one pathway (top one) was considered for further discussion.

Based on the investigation of different isomers of **AA**, the structure in which the chain-end alkoxide coordinates with both Cr centers (**AA-2**) exhibits higher energy than the structure in which the alkoxide and another ether oxygen coordinate independently with the two Cr centers (**AA**). Moreover, the **H-H** conformation is also preferred in **AA** (among **AA-1**–**AA-5**). Overall, **AA-1** is identified as the most stable structure and was selected for further discussion.

There are two possible **H-T** conformations, referred to as **a** and **b** (Figure S4C). By comparing the relative energy barriers for the ring-opening of (*R*)/(*S*)-PO, conformation **a** is found to be preferred and is therefore adopted for further discussion in the main text. The data in Figure S4C are referenced to the transition state of (*S*)-PO ring-opening in the **H-H** conformation (**TS**, Figure S4C).

Table S1. All geometric parameters of catalyst during PO ring-opening

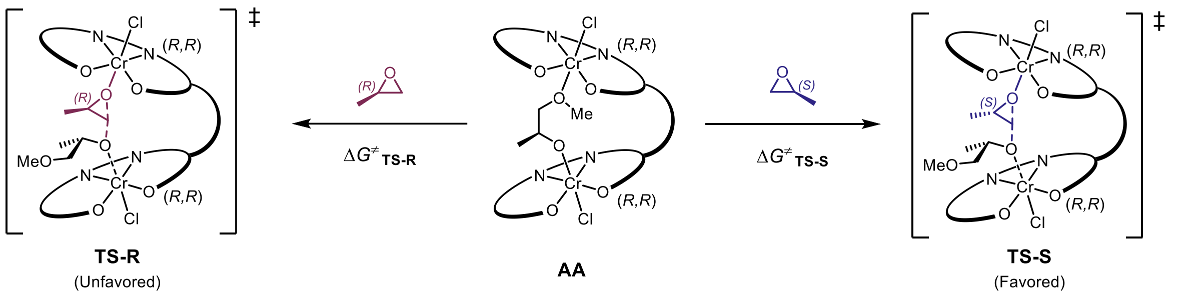

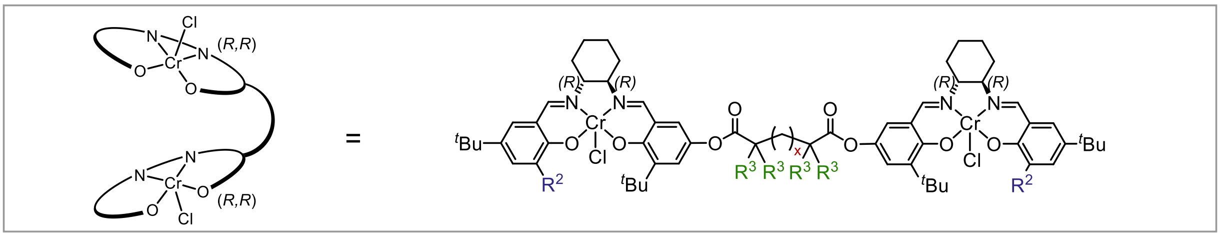

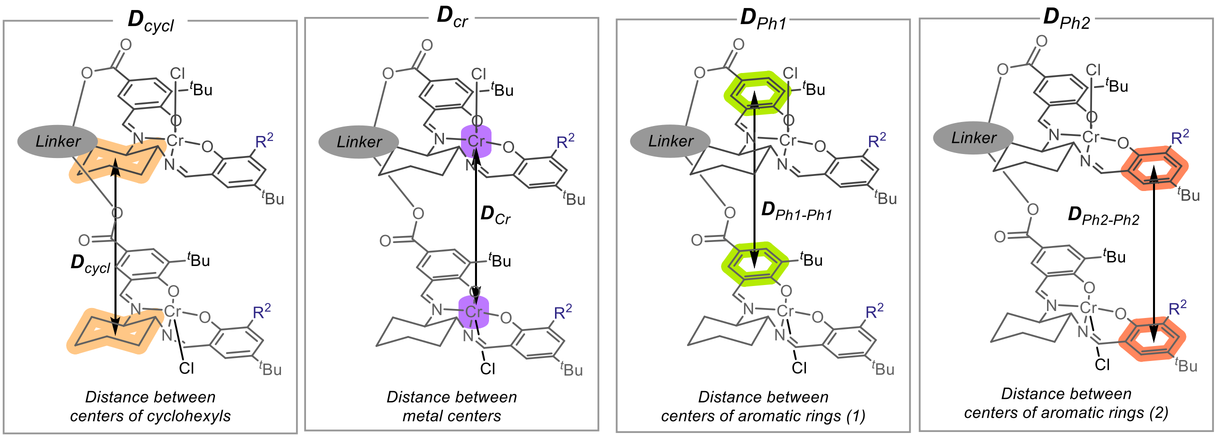

				**AA**				**TS-S** (favored)					**TS-R** (unfavored)			
Cat	R^2	R^3	x	Distance (Å)				Distance (Å)				$\Delta G^{\ddagger}_{\text{TS-S}}$	Distance (Å)				$\Delta G^{\ddagger}_{\text{TS-R}}$
				Cr	Cycl	Ph1	Ph2	Cr	Cycl	Ph1	Ph2	(kcal/mol)	Cr	Cycl	Ph1	Ph2	(kcal/mol)
**7**	Me	H	4	6.82	7.39	4.48	10.90	6.88	7.78	4.59	10.98	20.6	6.78	5.51	4.58	11.62	22.8
**1**	^{t}Bu	H	4	6.85	7.25	4.51	10.81	6.95	7.79	4.65	10.76	18.9	6.80	5.59	4.59	11.50	23.1
**8**	Ad	H	4	6.84	7.53	4.90	11.13	6.96	7.86	4.62	10.77	18.1	6.90	5.85	4.65	10.36	22.7
**9**	CPh_3	H	4	6.78	6.57	4.50	11.53	7.04	7.50	5.00	10.65	26.9	7.07	5.53	4.80	10.79	37.0
**10**	^{t}Bu	Me	4	6.65	5.85	4.70	11.23	6.95	7.79	4.59	10.78	16.5	6.84	5.88	4.79	11.51	20.0
**11**	^{t}Bu	Me	5	6.82	6.64	4.42	10.87	6.91	7.90	4.99	10.82	16.7	6.91	5.89	5.04	11.49	20.3
**12**	^{t}Bu	Me	3	6.79	5.27	5.00	9.79	6.96	7.68	4.48	10.77	14.7	6.83	5.61	4.60	11.45	18.1
**13**	Ad	Me	3	6.80	5.45	5.50	8.58	6.93	7.58	4.47	10.73	15.4	6.85	5.72	4.62	11.18	20.3

More discussion: The distances between the two Cr centers are constrained in **AA** and **TS**s due to structural characteristics. The distances between the centers of the aromatic rings are consistent with the trend observed for D_{cycl}.

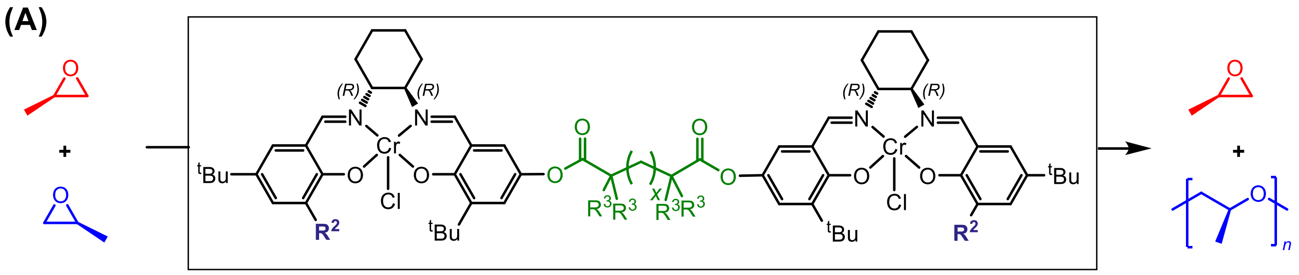

catalyst	R^2	R^3	x	D_{cycl} (Å)	$\Delta G^{\neq}$ (kcal/mol)	TOF (h^{-1})	ln[TOF (h^{-1})]
**1**	^{t}Bu	H	4	7.25	18.9	643	6.47
**8**	Ad	H	4	7.53	18.1	982	6.89
**10**	^{t}Bu	Me	4	5.85	16.5	2592	7.86
**11**	^{t}Bu	Me	5	6.64	16.7	1970	7.59
**12**	^{t}Bu	Me	3	5.27	14.7	6153	8.72
**13**	Ad	Me	3	5.45	15.4	5004	8.52

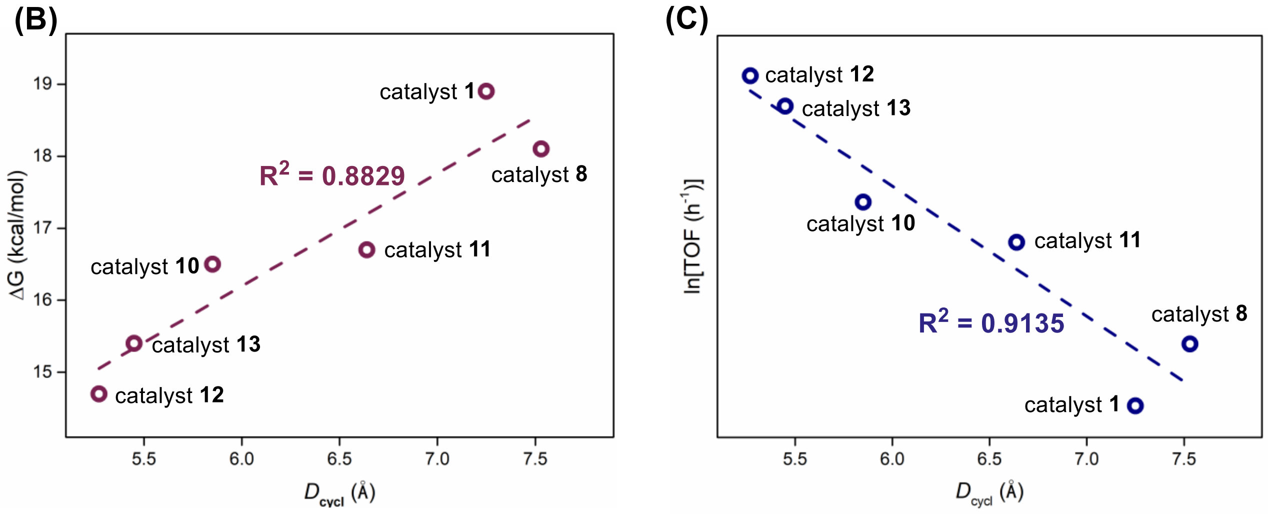

Figure S5. Linear relationship between D_{cycl} and activity. (A) Detailed data; (B) D_{cycl}–ΔG correlation; (C) D_{cycl}–ln(TOF) correlation.

4. Polymer Properties

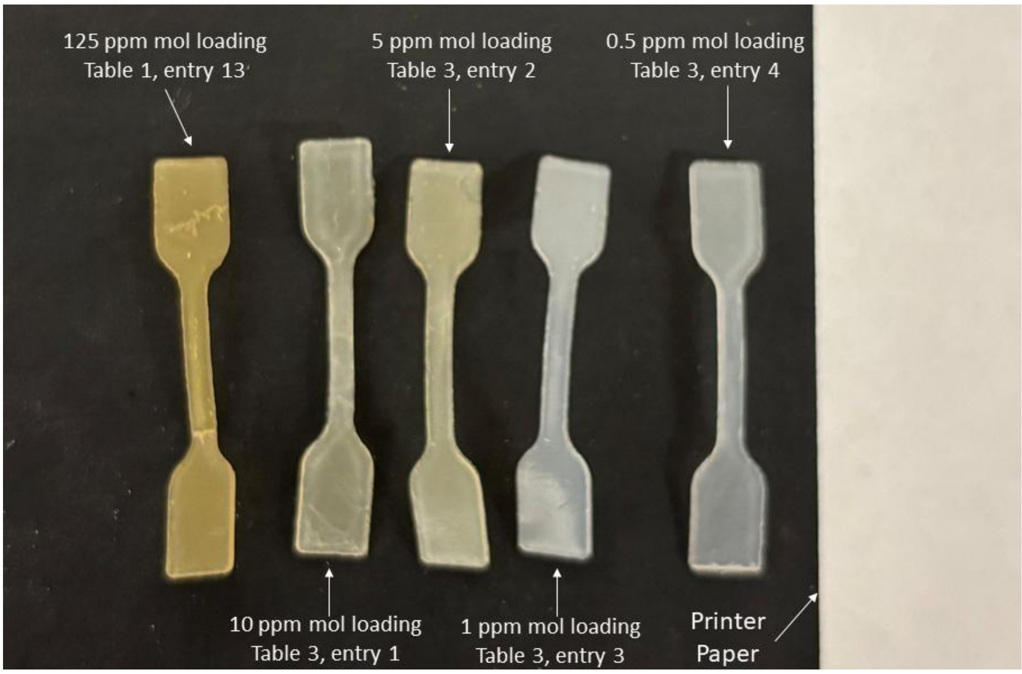

Figure S6. Color of resulting *i*PPO due to catalyst residues.

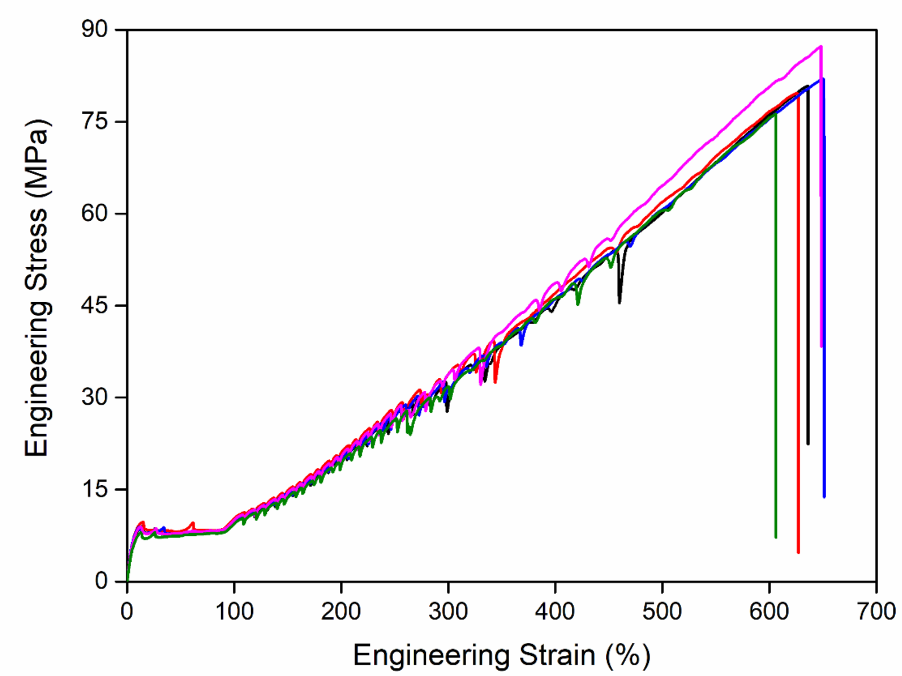

Figure S7. Stress–strain curve of *i*PPO with M_n of 296 kDa (Table 3, entry 4). The resulting tensile strength of 81 MPa is also close to the previous report (~75 MPa).[2]

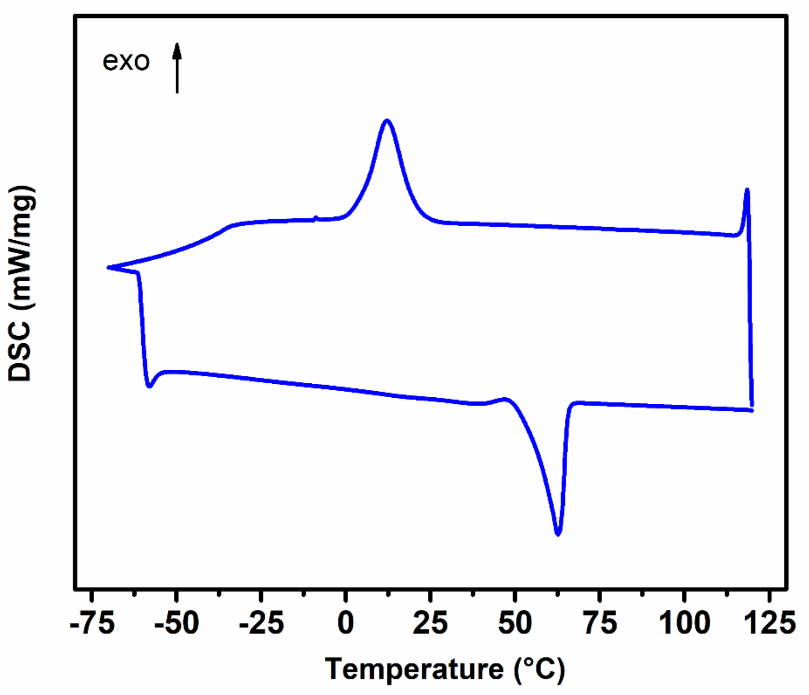

Figure S8. DSC thermogram of *i*PPO with M_n of 296 kDa (Table 3, entry 4).

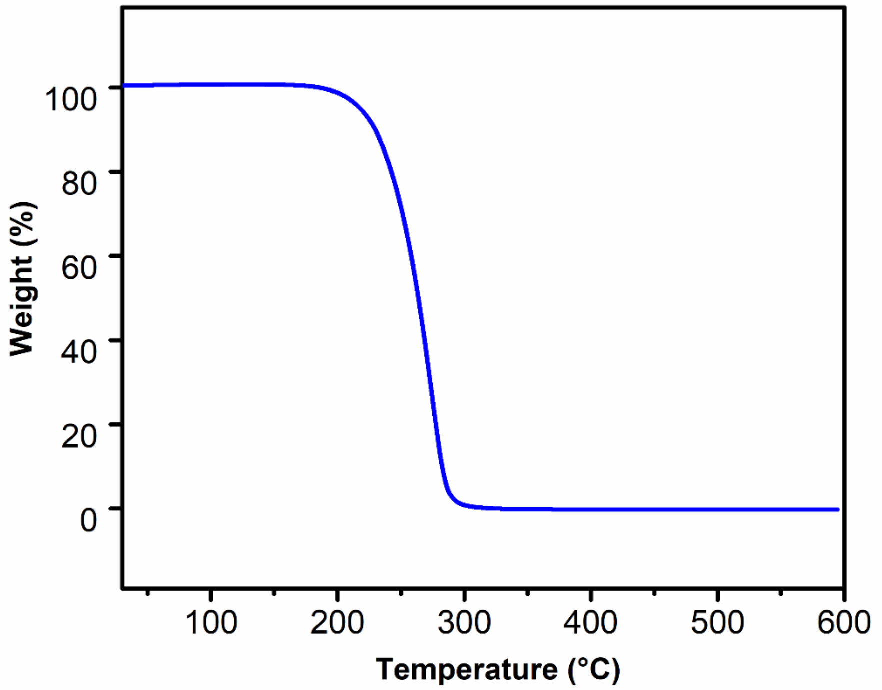

Figure S9. TGA thermogram of *i*PPO with M_n of 296 kDa (Table 3, entry 4)

5. Polymerization of Other Epoxides

Table S2. Polymerization of other epoxides

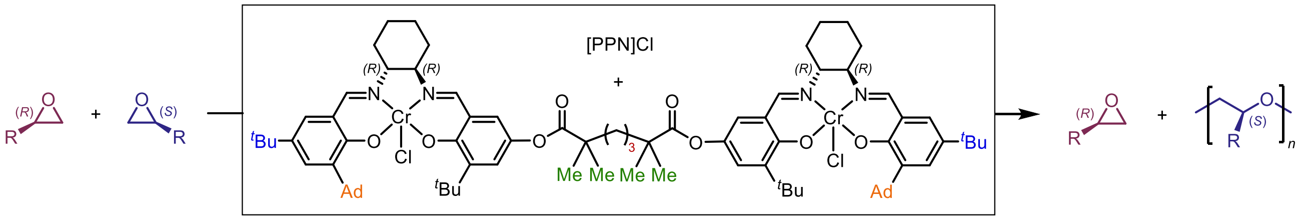

Epoxide	R	Time (h)	Conv. (%)	TOF (h^{-1})[c]	[*mm*][d]	k_{rel}[e]	M_n (kDa)[f]	$Đ$[f]
1,2-Epoxybutane (BO)[a]	Et	1.5	24	1209	>99%	>300	49.7	2.59
1,2-Epoxyhexane (HO)[b]	^{n}Bu	6.5	33	203	91%	38	30.4	2.02

[a]Polymerization condition: Polymerization in neat BO, [Cat]/[PPNCl]/[BO] = 1/1/8000, temperature = 23 °C, and conversion was determined gravimetrically; [b]Polymerization condition: Polymerization in neat HO; [Cat]/[PPNCl]/[BO] = 1/1/4000, Temperature = 23 °C, and conversion was determined by ^{1}H NMR. [c]mmol epoxide consumed · mmol catalyst^{-1} · time^{-1}. [d]Determined by ^{13}C NMR spectroscopic analysis. [e]Calculated based on tacticity and conversion. [f]Determined by GPC in THF, calibrated with polystyrene standards

Table S3. Reproducibility Study of PO Polymerization

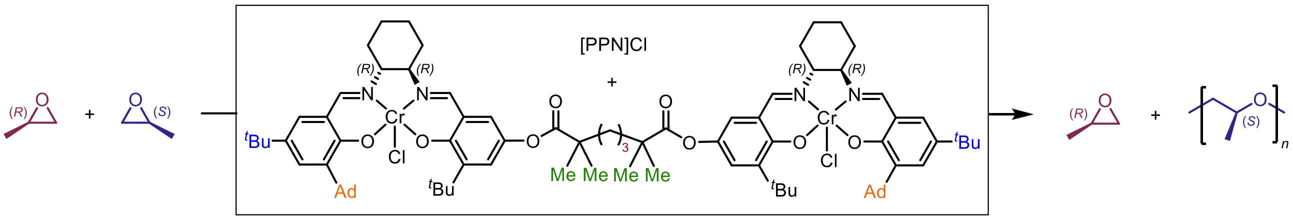

Cat. Loading	Entry	Time	Conv. (%)	TOF (10^5 h^{-1})	[*mm*]	$ee_{(p)}$	k_{rel}
10 ppm	Run 1	42 min	40.9	5.85	95.9%	96.1%	101
	Run 2	50 min	47.9	5.75	92.4%	93.7%	91
	Run 3	55 min	49.4	5.39	92.2%	93.5%	96
	Average			5.66 ± 0.24			96 ± 5
5 ppm	Run 1	90 min	26.4	3.52	96.1%	96.9%	95
	Run 2	110 min	33.7	3.67	96.1%	96.6%	91
	Run 3	120 min	37.7	3.79	95.0%	96.0%	88
	Average			3.66 ± 0.14			96 ± 4
1 ppm	Run 1	11 h	27.9	2.54	96.4%	97.0%	95
	Run 2	15 h	35.3	2.35	95.4%	96.5%	95
	Run 3	20 h	47.8	2.35	92.5%	94.2%	94
	Average			2.41 ± 0.11			95 ± 1
0.5 ppm	Run 1	24 h	27.3	2.27	96.0%	96.7%	85
	Run 2	39 h	39.4	2.02	93.8%	95.4%	80
	Run 3	48 h	46.0	1.92	92.7%	94.6%	89
	Average			2.07 ± 0.18			85 ± 5

All conditions are same with Table 3.

6. Polymerization Characterization Data

GPC, ^{1}H NMR, and ^{13}C NMR data are included here for all polymerizations that yielded polymer:

Table 1, entry 1:

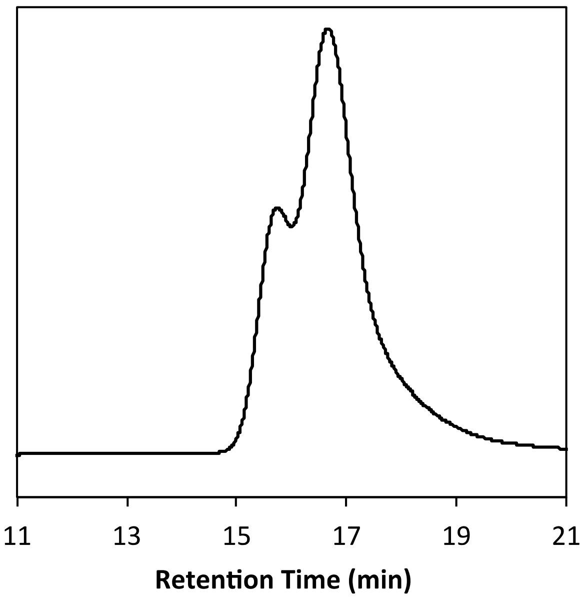

GPC chromatogram of the polymer of Table 1, entry 1

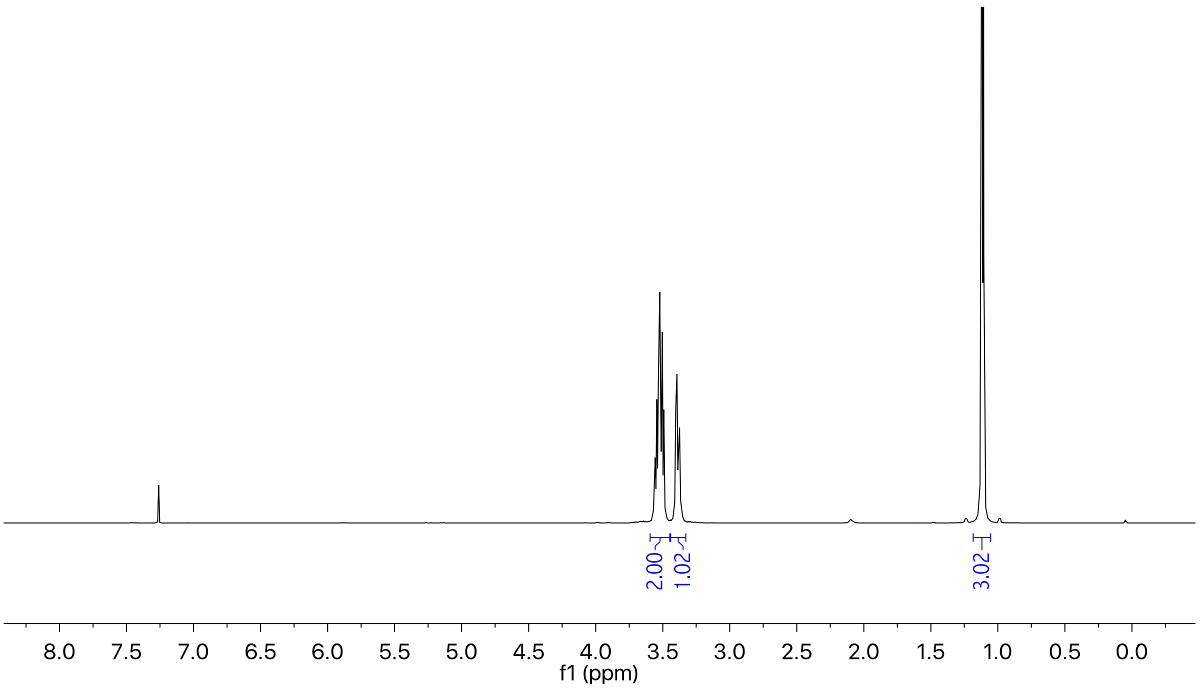

^{1}H NMR spectrum of the polymer of Table 1, entry 1

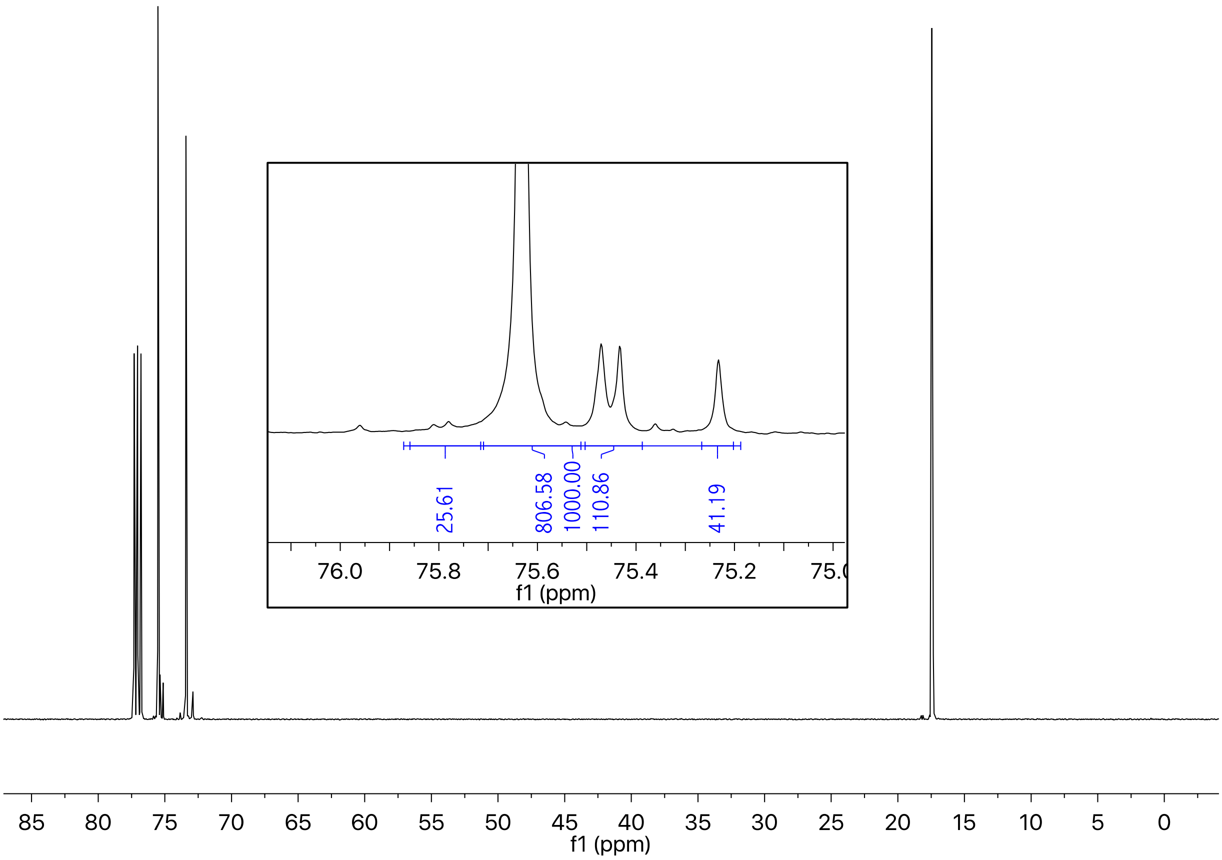

^{13}C NMR spectrum of the polymer of Table 1, entry 1

Table 1, entry 2:

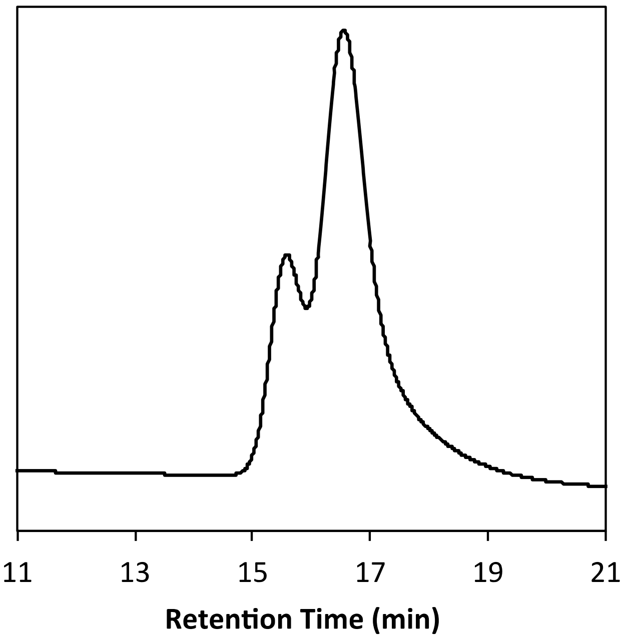

GPC chromatogram of the polymer of Table 1, entry 2

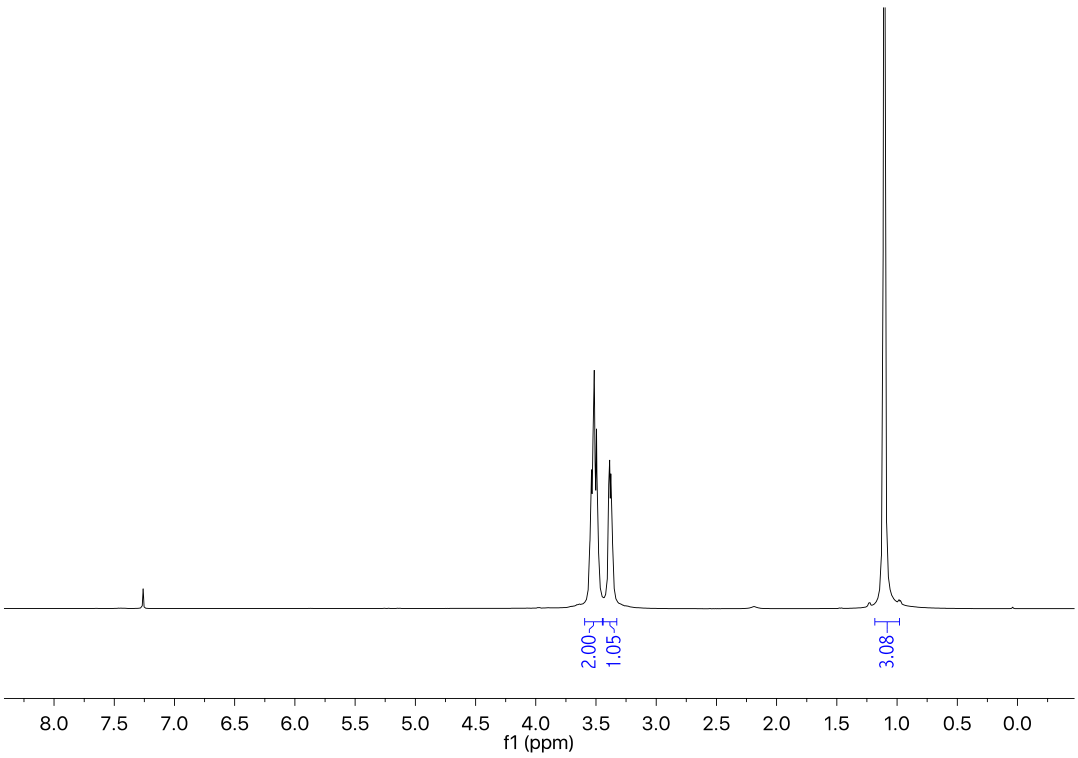

^{1}H NMR spectrum of the polymer of Table 1, entry 2

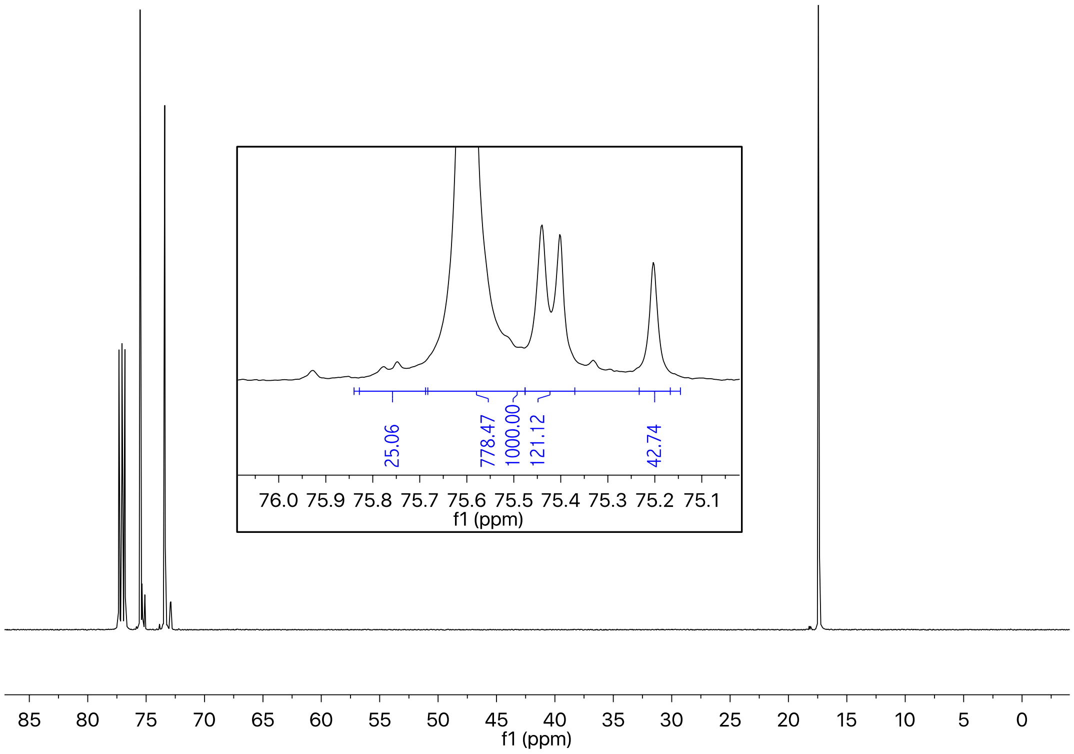

^{13}C NMR spectrum of the polymer of Table 1, entry 2

Table 1, entry 3:

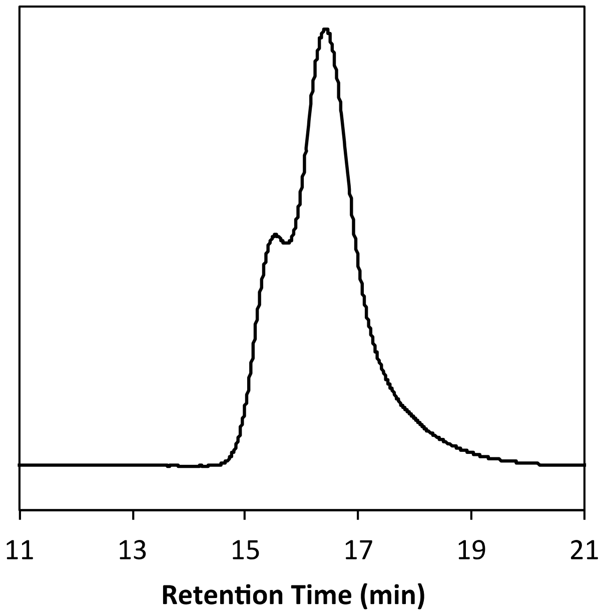

GPC chromatogram of the polymer of Table 1, entry 3

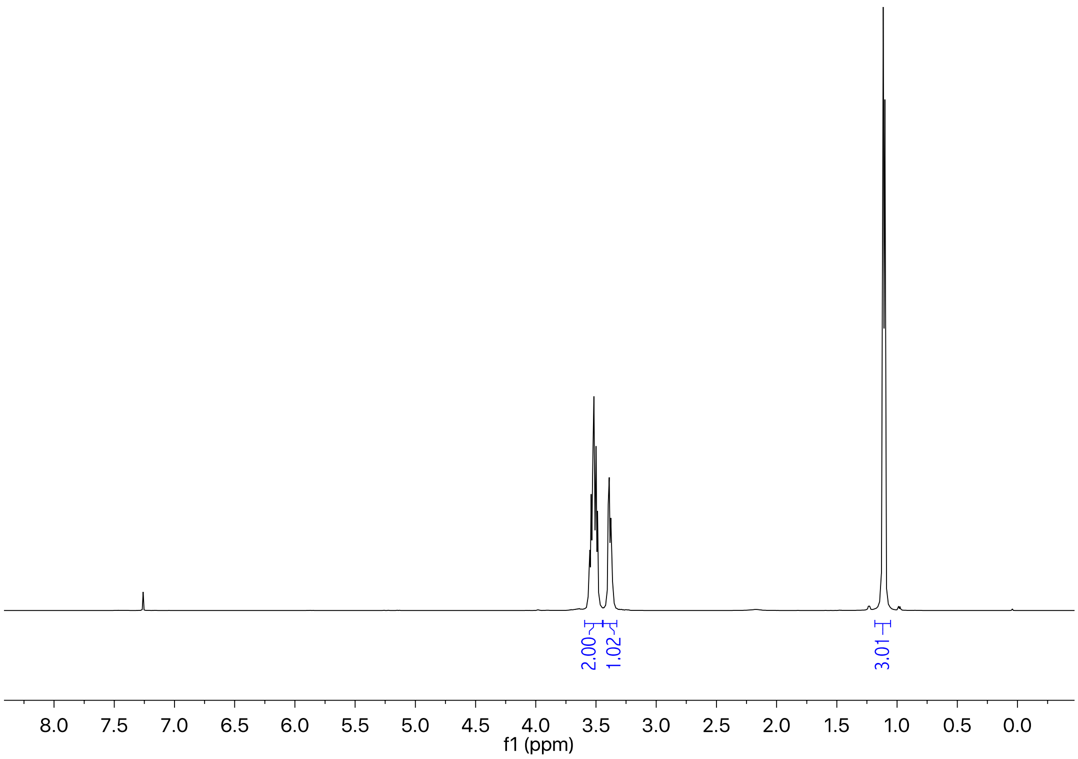

^{1}H NMR spectrum of the polymer of Table 1, entry 3

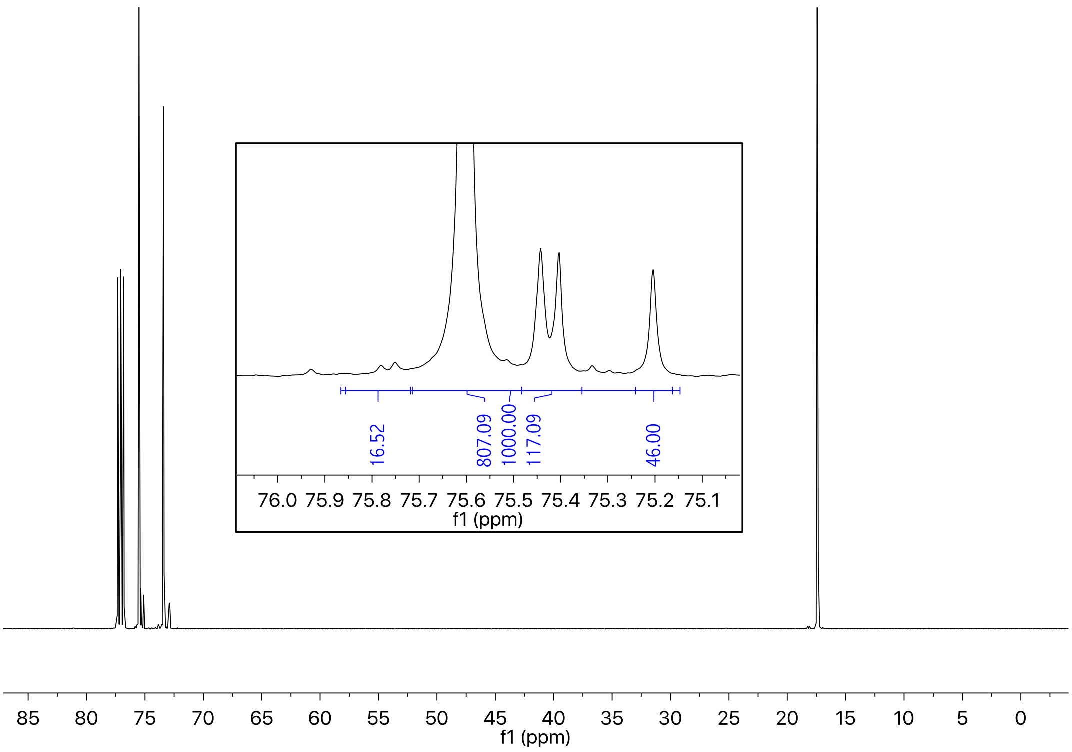

^{13}C NMR spectrum of the polymer of Table 1, entry 3

Table 1, entry 4:

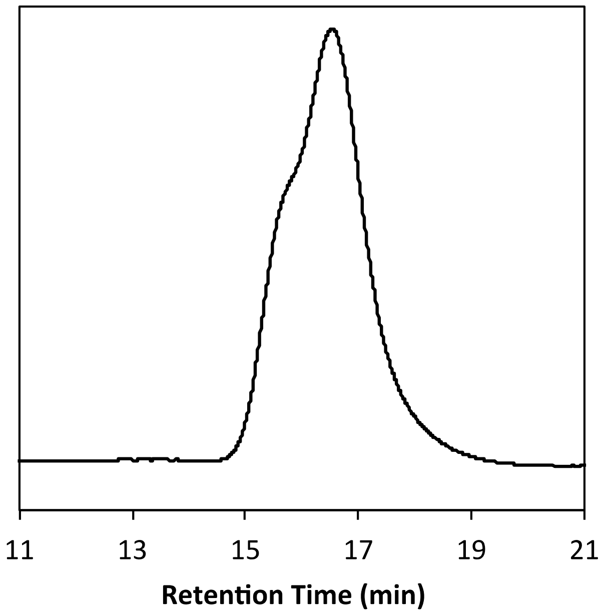

GPC chromatogram of the polymer of Table 1, entry 4

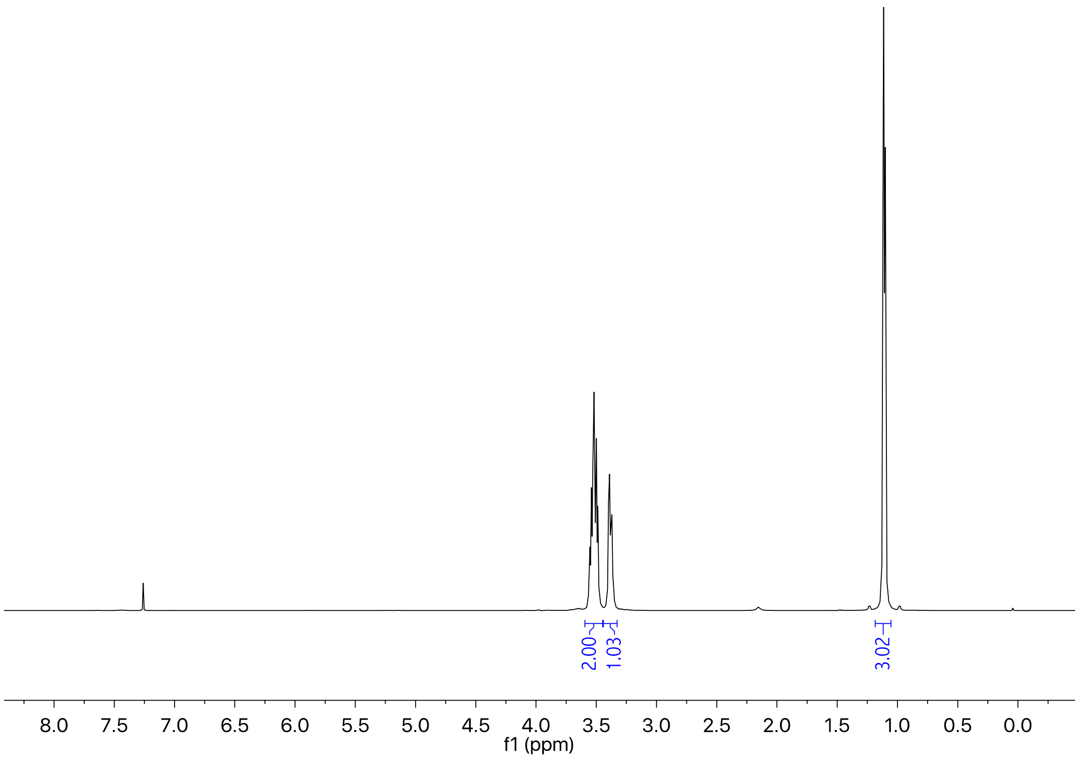

^{1}H NMR spectrum of the polymer of Table 1, entry 4

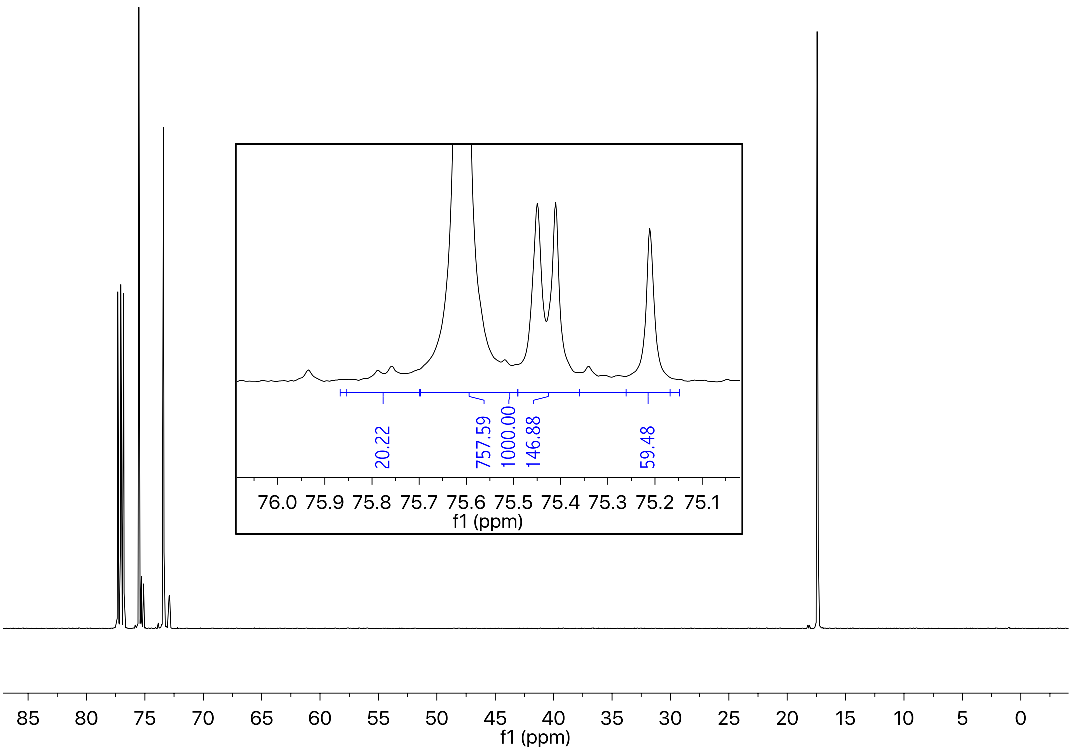

^{13}C NMR spectrum of the polymer of Table 1, entry 4

Table 1, entry 5:

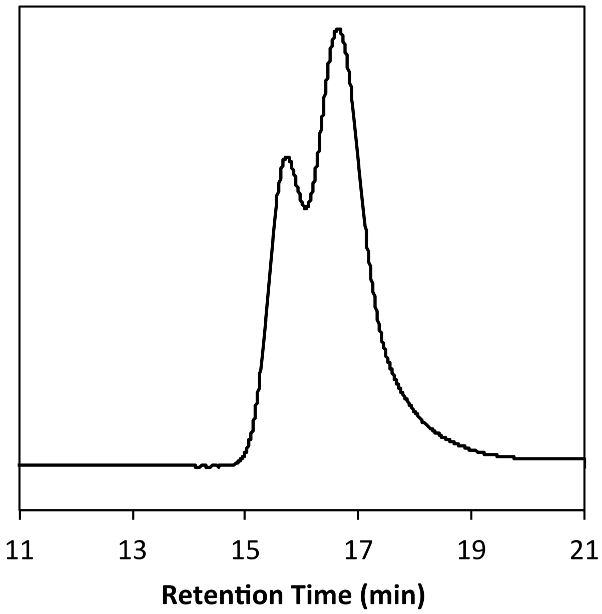

GPC chromatogram of the polymer of Table 1, entry 5

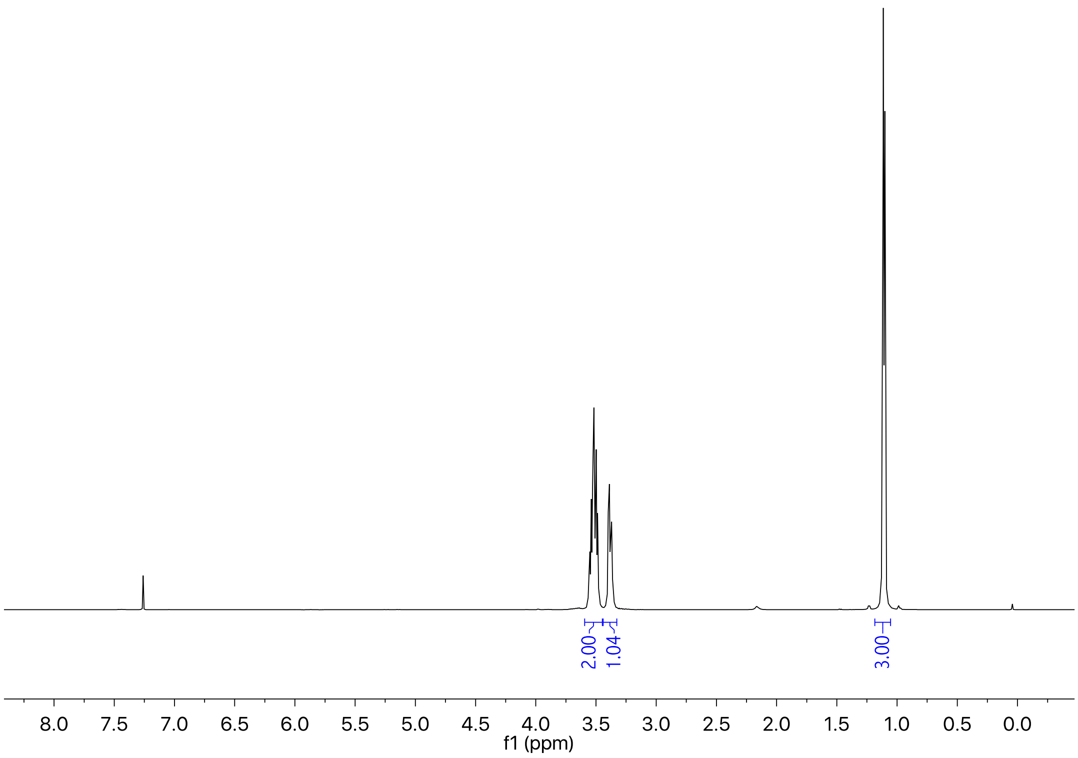

^{1}H NMR spectrum of the polymer of Table 1, entry 5

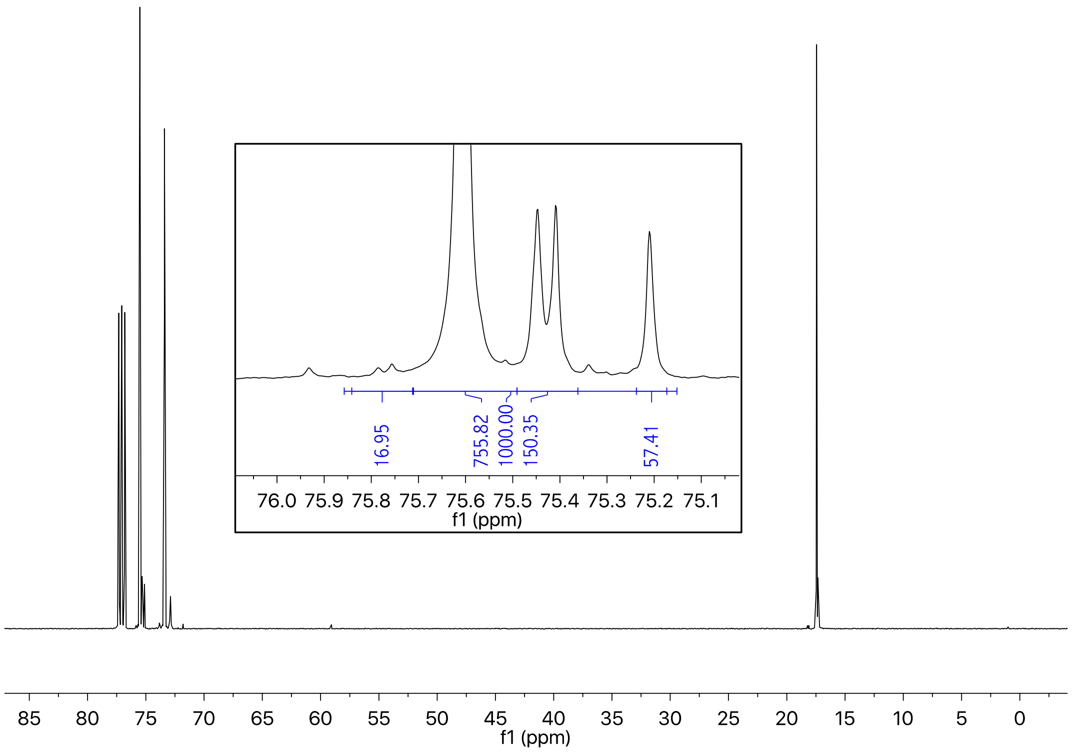

^{13}C NMR spectrum of the polymer of Table 1, entry 5

Table 1, entry 6:

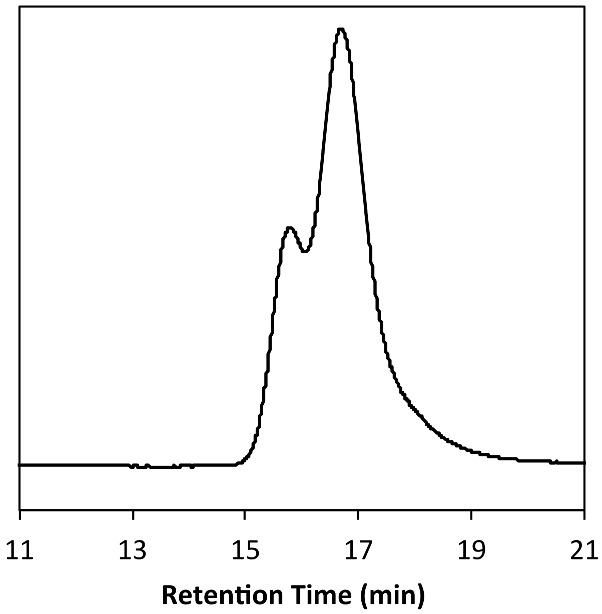

GPC chromatogram of the polymer of Table 1, entry 6

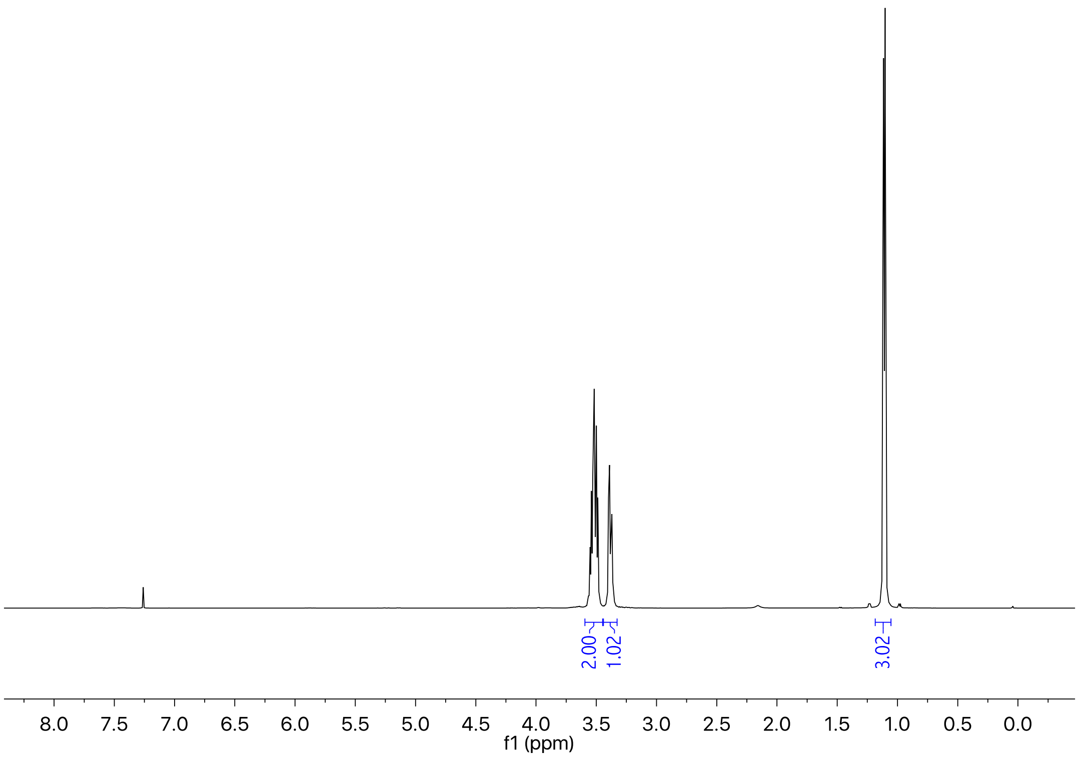

^{1}H NMR spectrum of the polymer of Table 1, entry 6

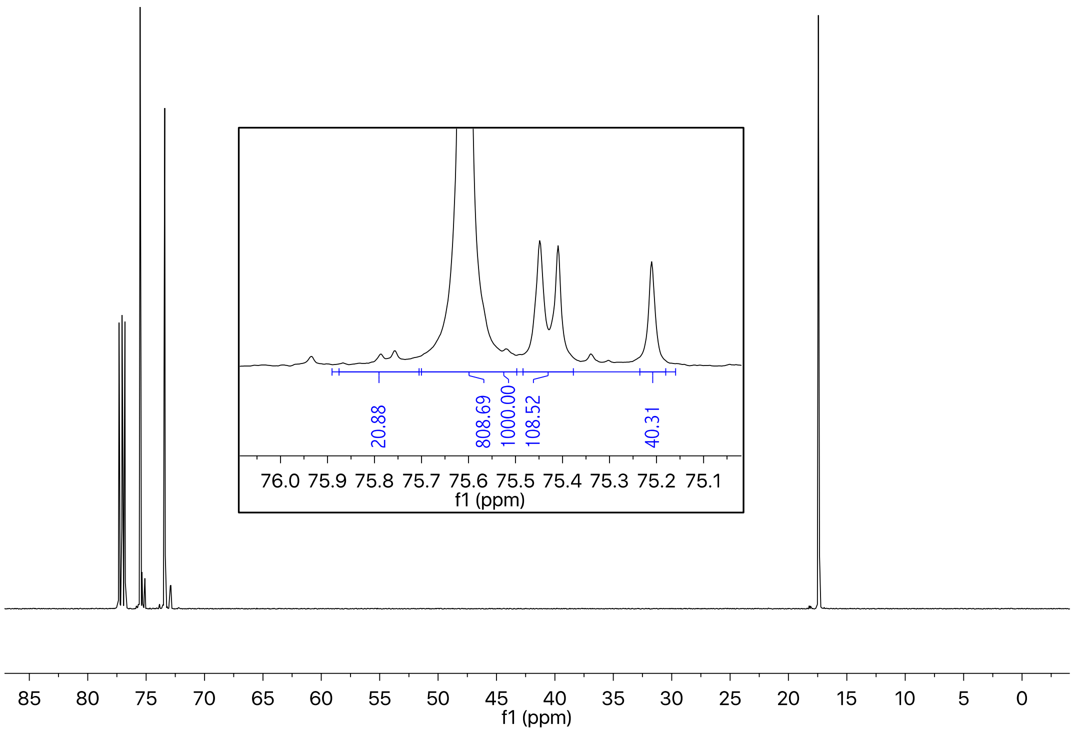

^{13}C NMR spectrum of the polymer of Table 1, entry 6

Table 1, entry 7:

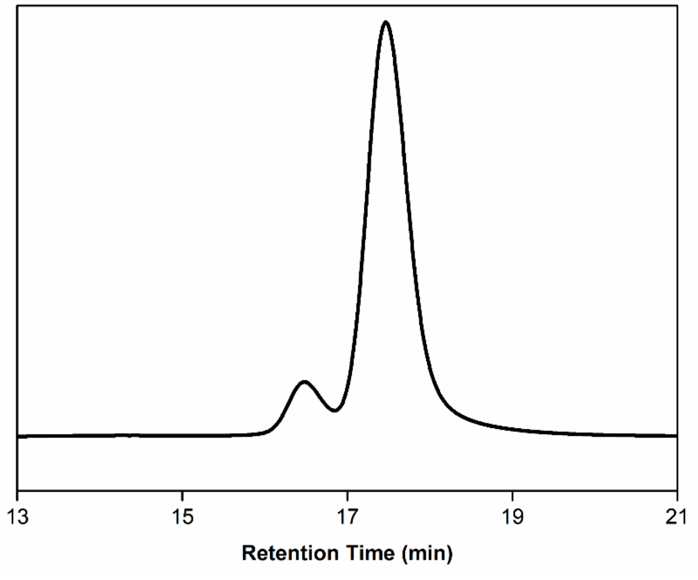

GPC chromatogram of the polymer of Table 1, entry 7

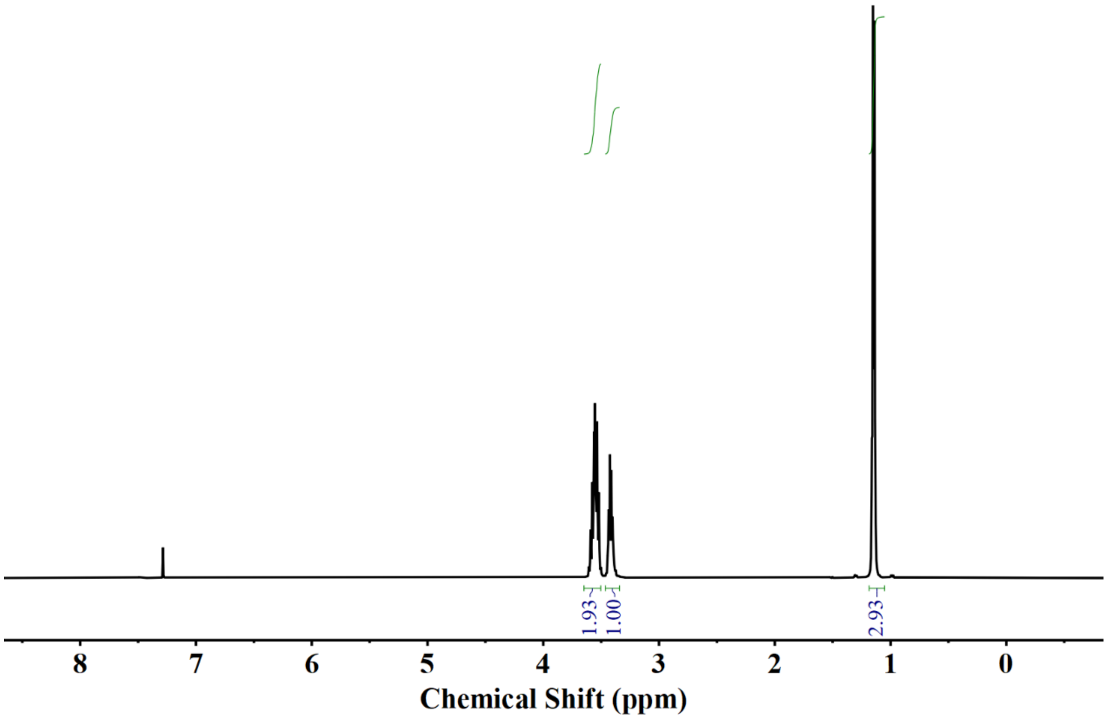

^{1}H NMR spectrum of the polymer of Table 1, entry 7

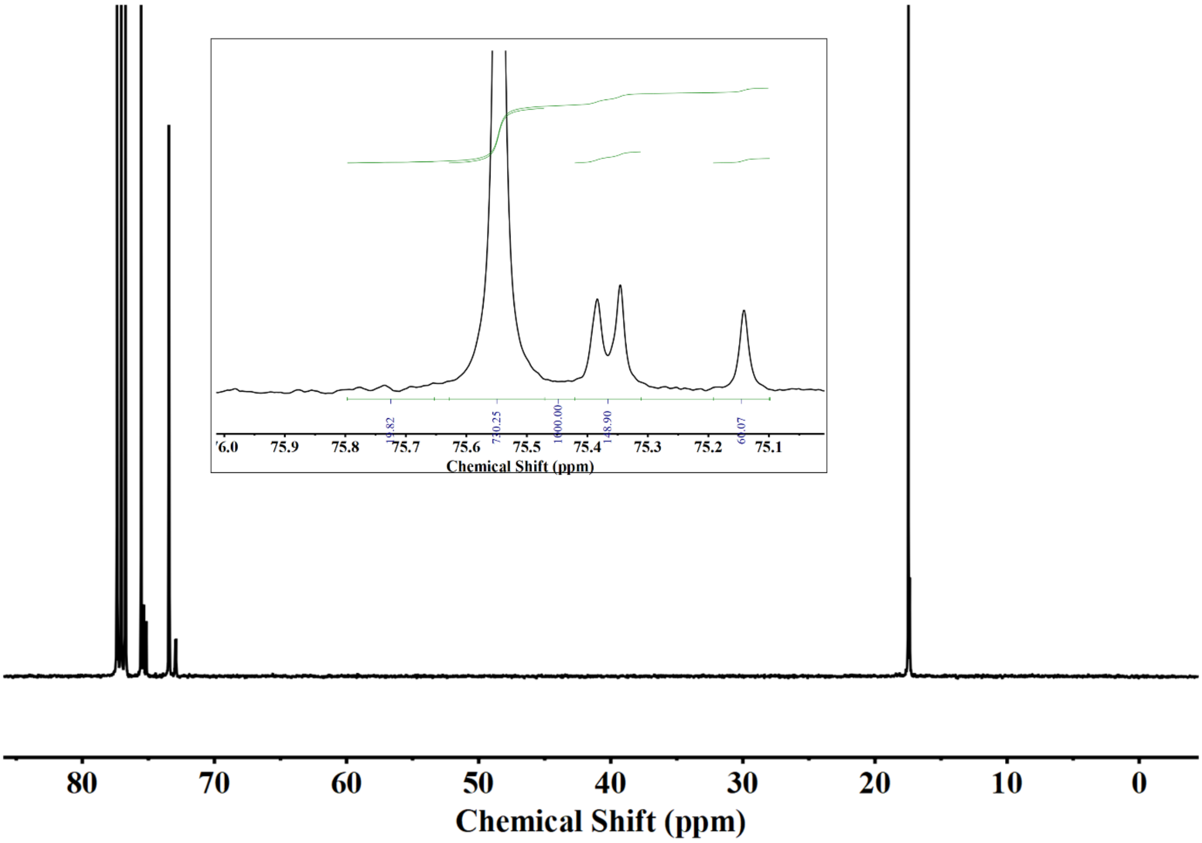

^{13}C NMR spectrum of the polymer of Table 1, entry 7

Table 1, entry 8:

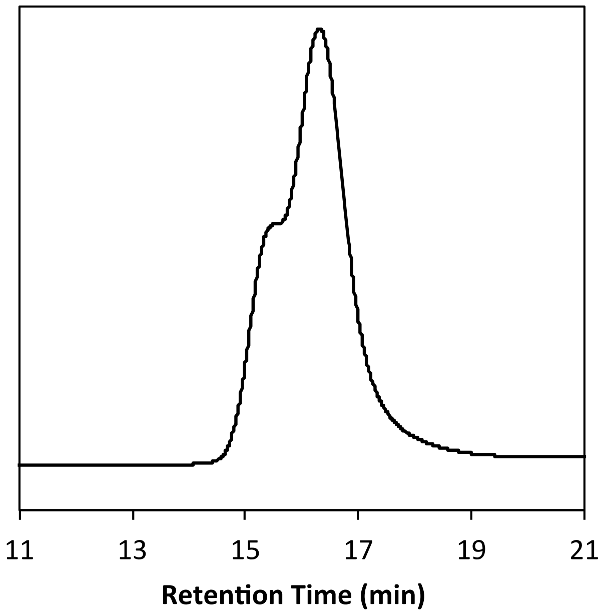

GPC chromatogram of the polymer of Table 1, entry 8

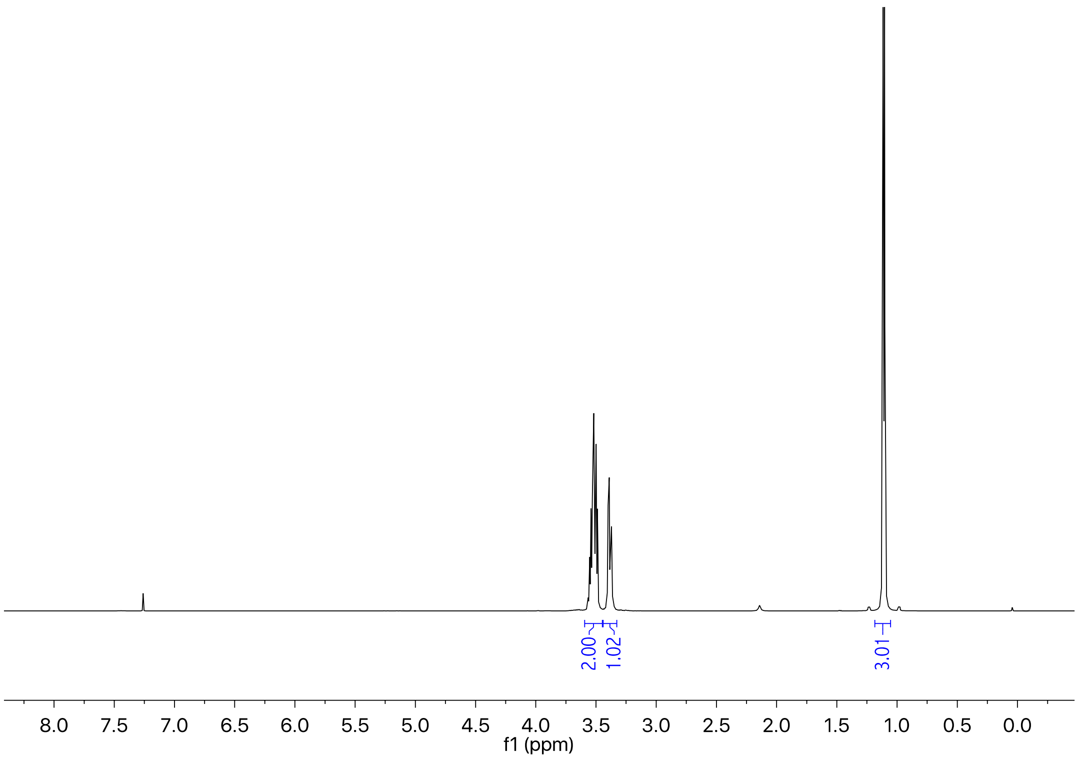

^{1}H NMR spectrum of the polymer of Table 1, entry 8

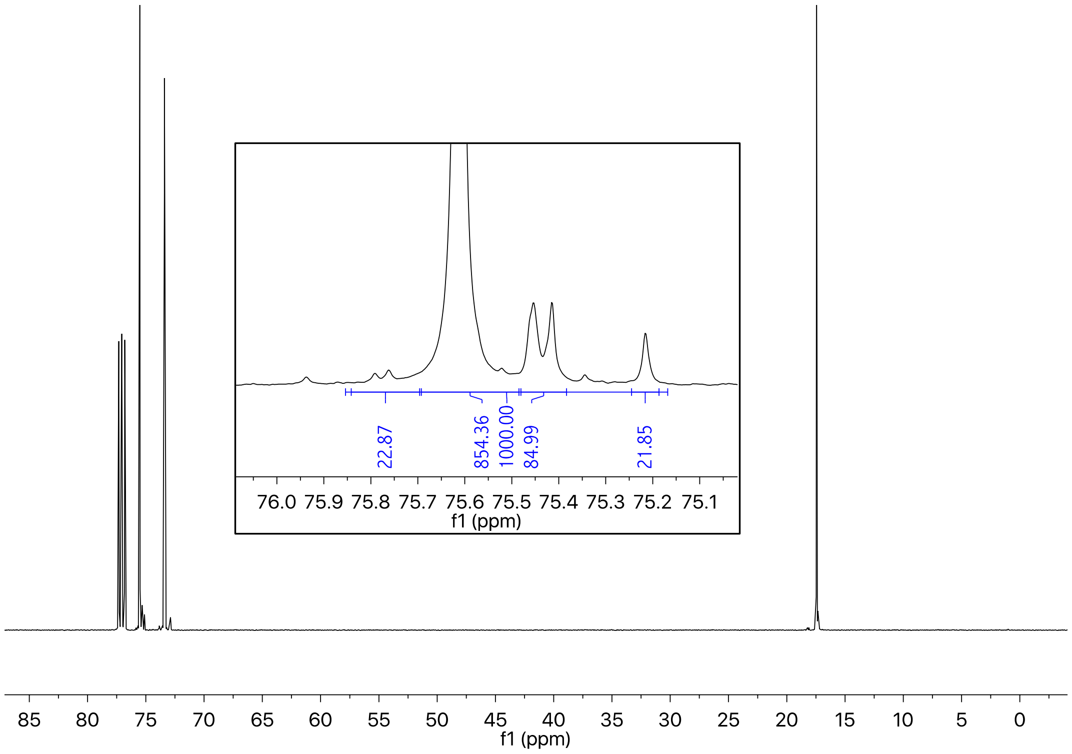

^{13}C NMR spectrum of the polymer of Table 1, entry 8

Table 1, entry 10:

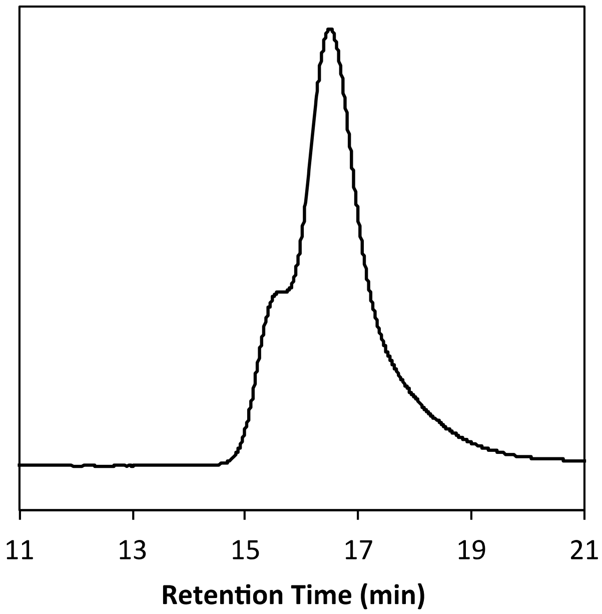

GPC chromatogram of the polymer of Table 1, entry 10

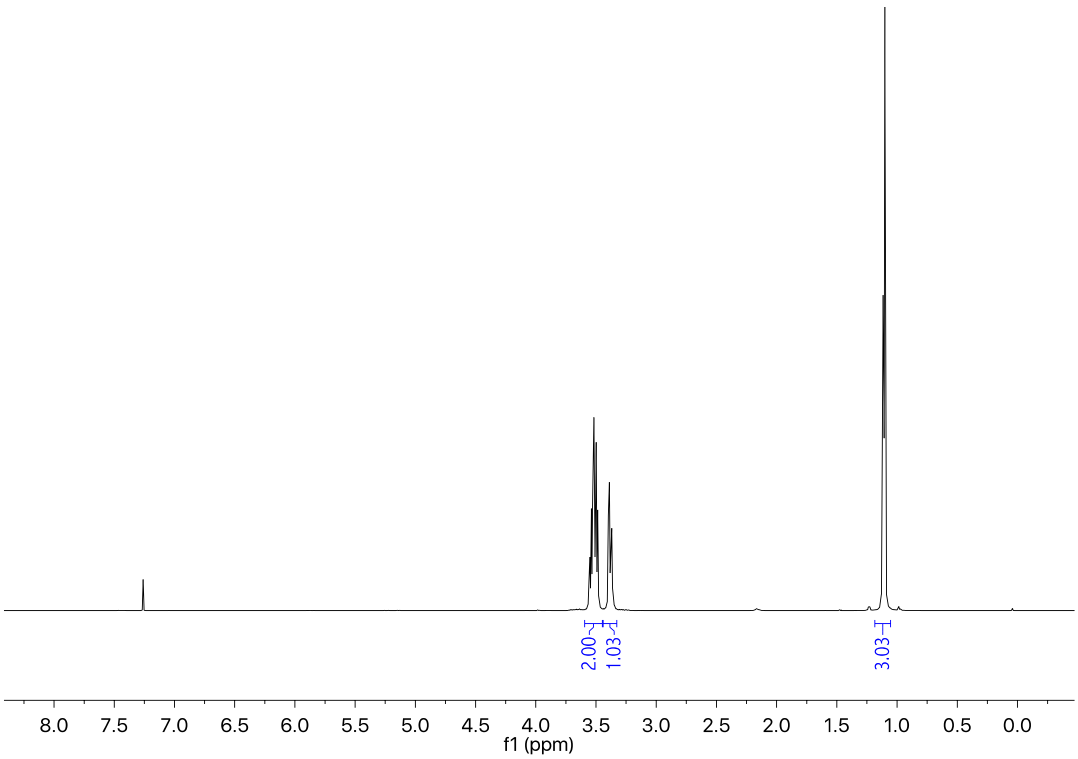

^{1}H NMR spectrum of the polymer of Table 1, entry 10

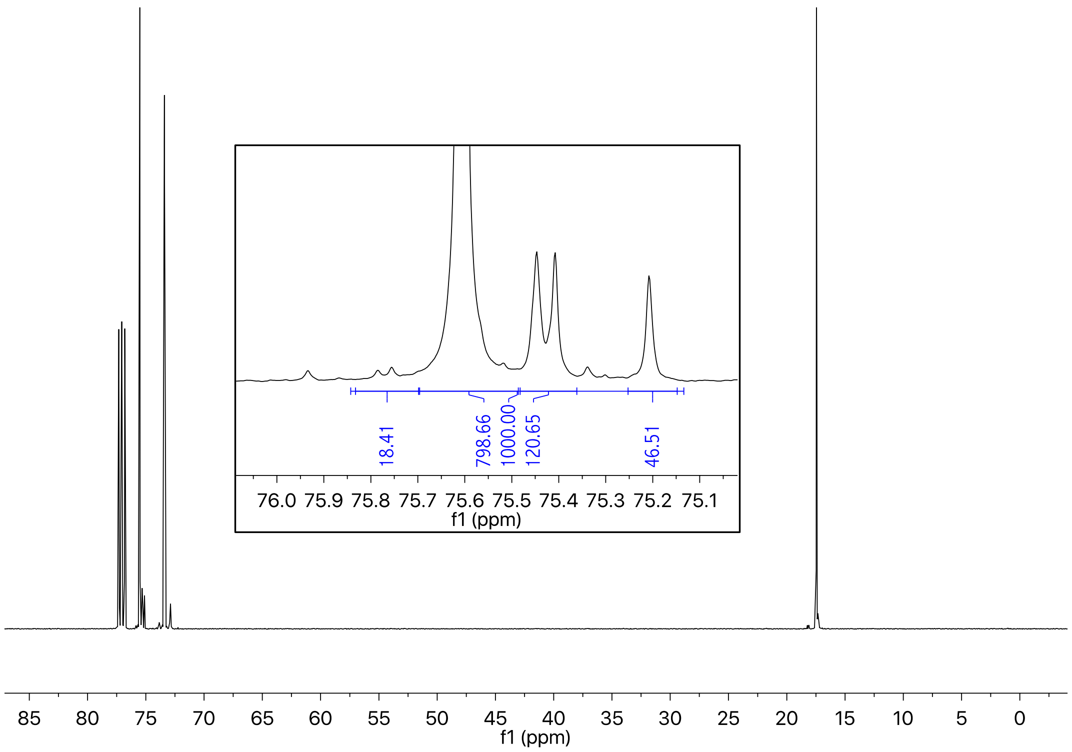

^{13}C NMR spectrum of the polymer of Table 1, entry 10

Table 1, entry 11:

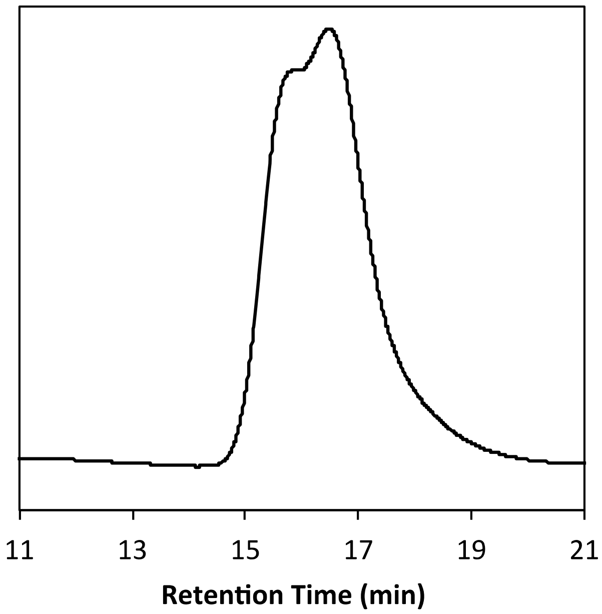

GPC chromatogram of the polymer of Table 1, entry 11

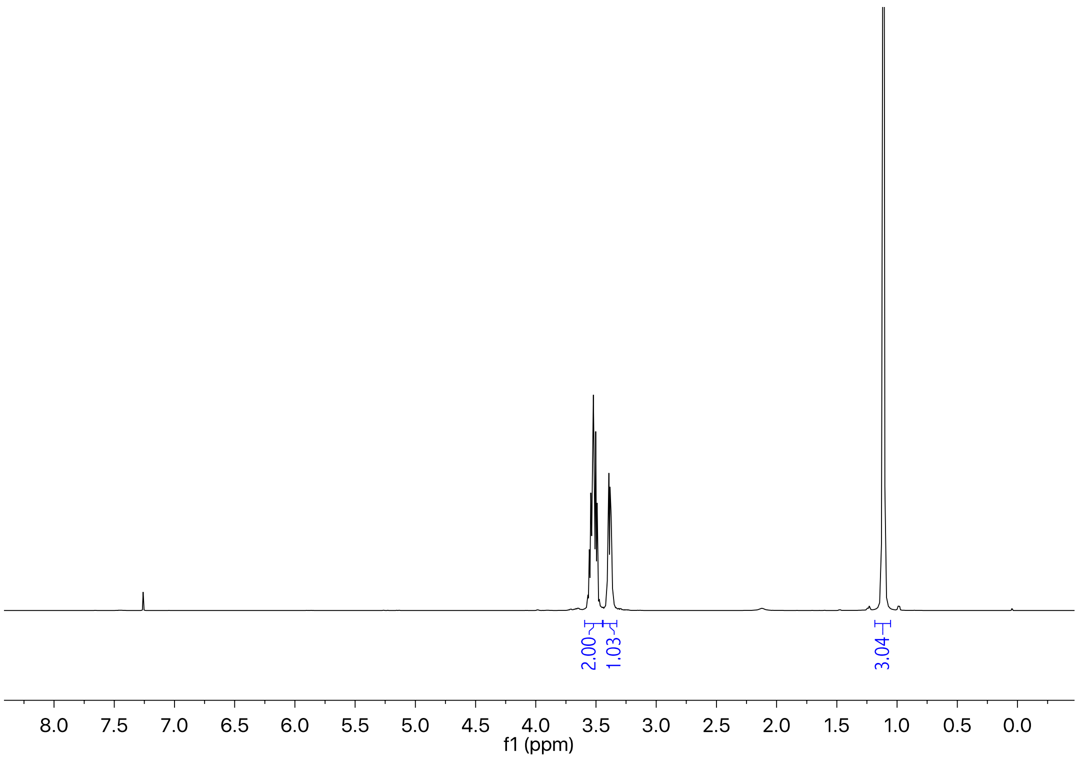

^{1}H NMR spectrum of the polymer of Table 1, entry 11

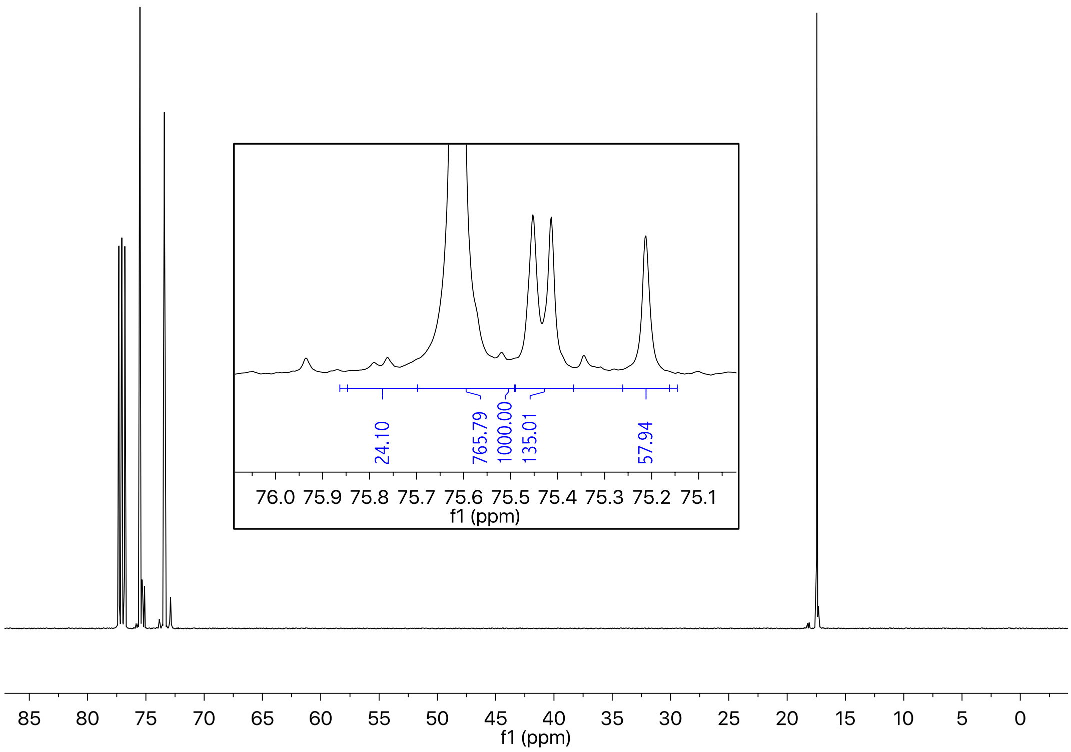

^{13}C NMR spectrum of the polymer of Table 1, entry 11

Table 1, entry 12:

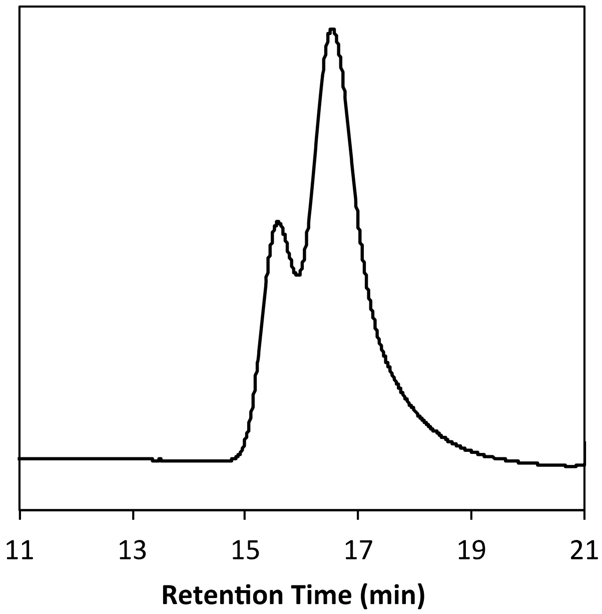

GPC chromatogram of the polymer of Table 1, entry 12

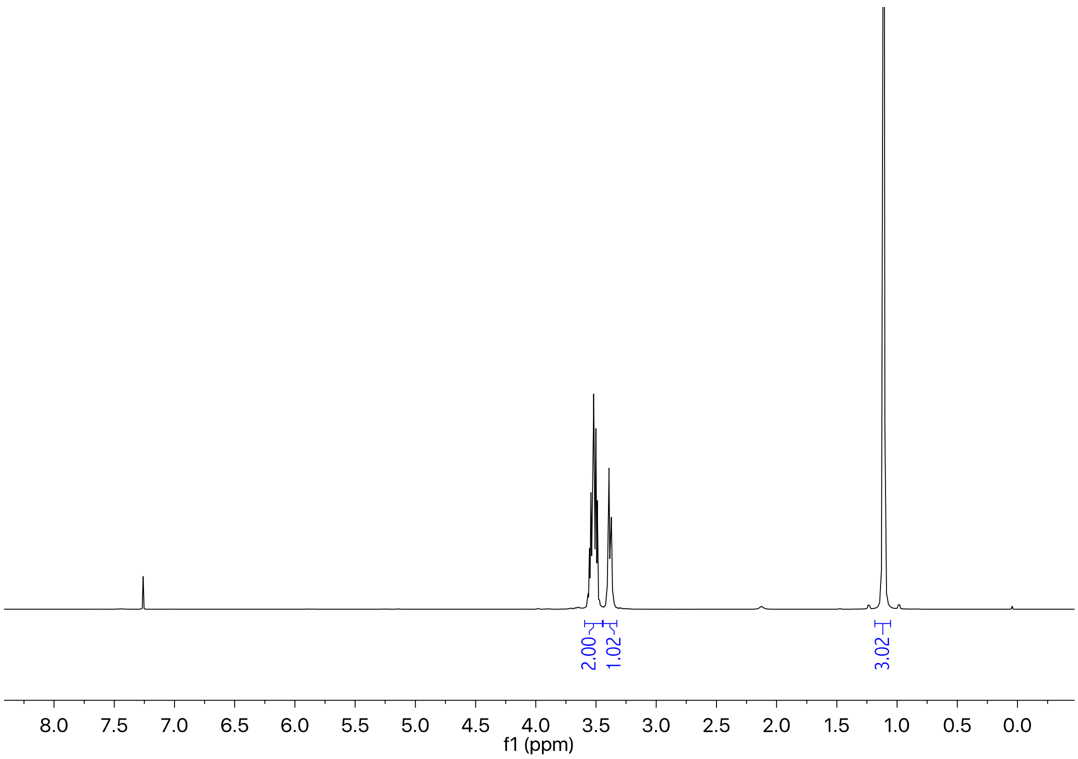

^{1}H NMR spectrum of the polymer of Table 1, entry 12

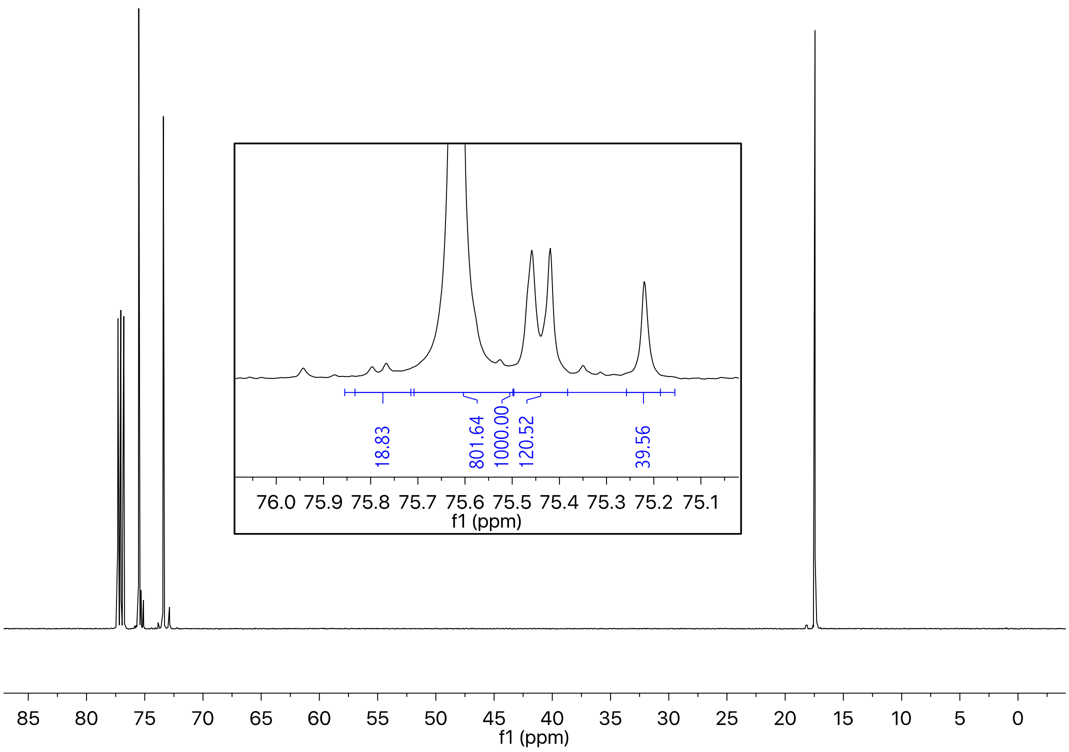

^{13}C NMR spectrum of the polymer of Table 1, entry 12

Table 1, entry 13:

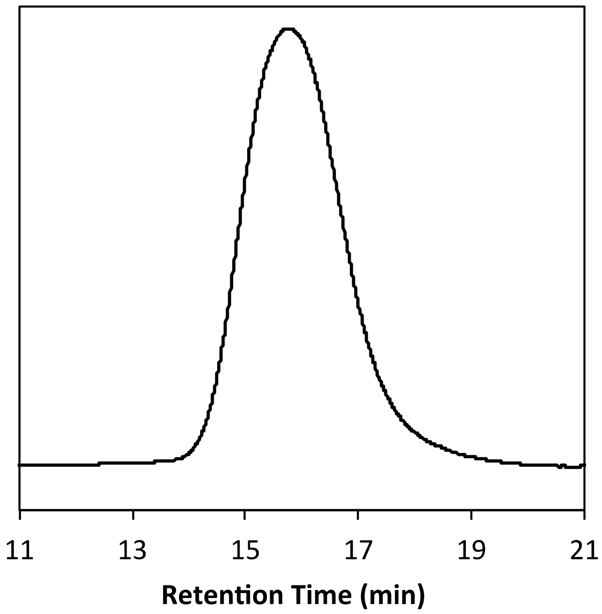

GPC chromatogram of the polymer of Table 1, entry 13

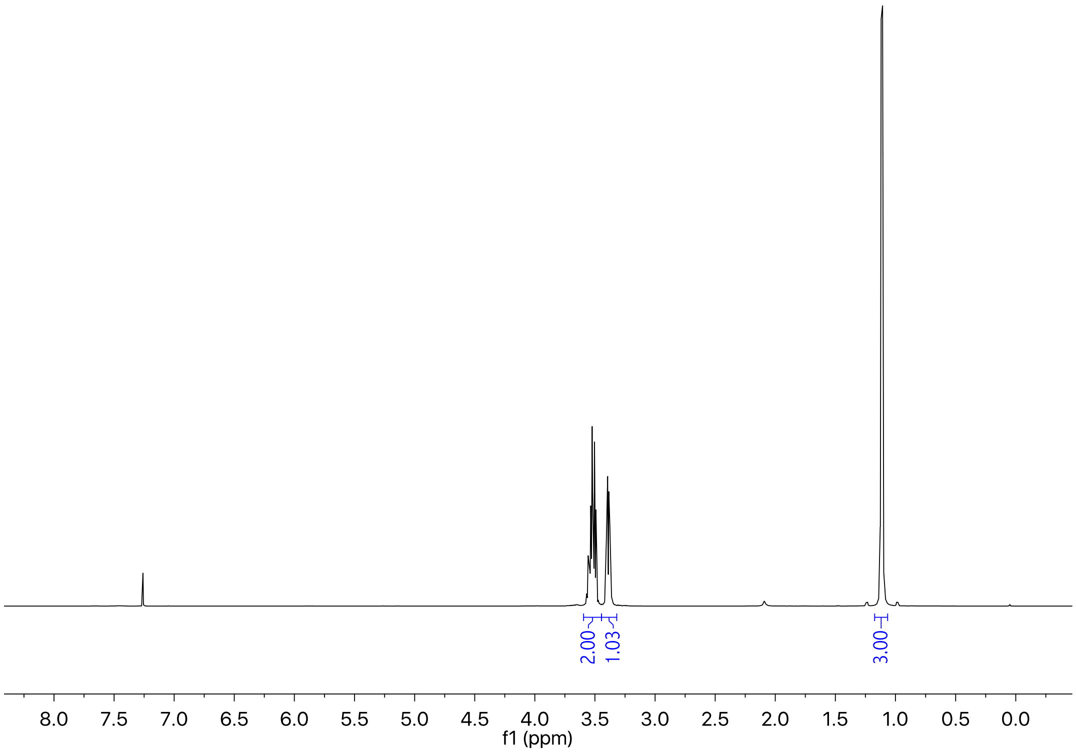

^{1}H NMR spectrum of the polymer of Table 1, entry 13

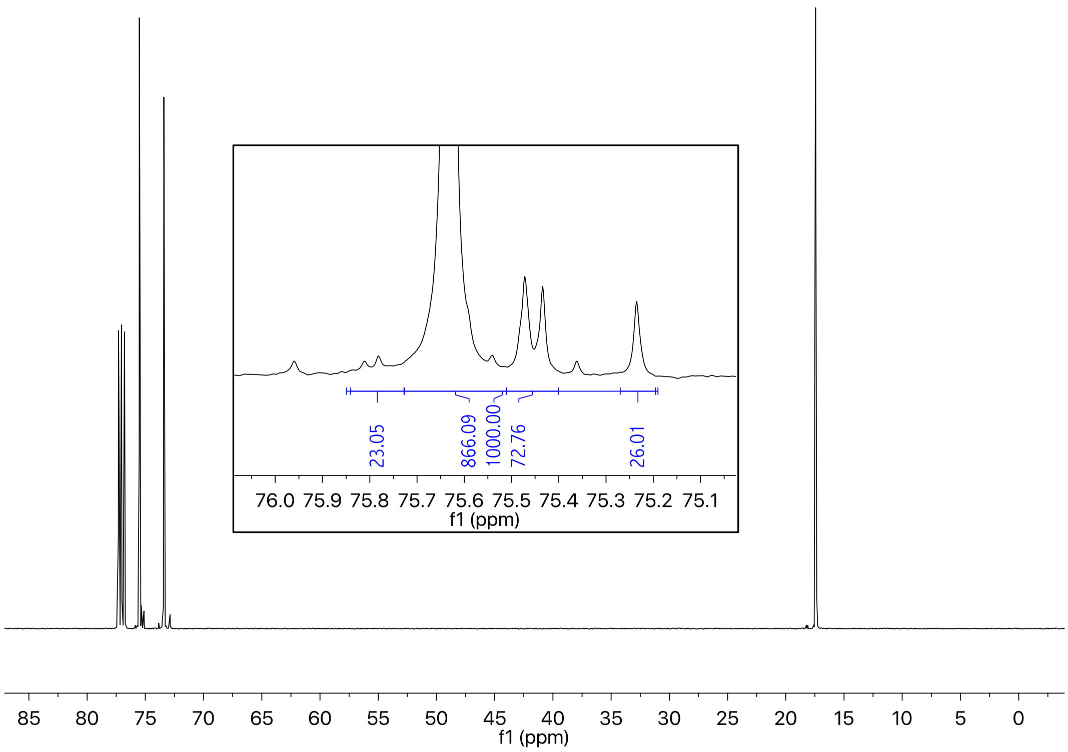

^{13}C NMR spectrum of the polymer of Table 1, entry 13

Table 3, entry 1:

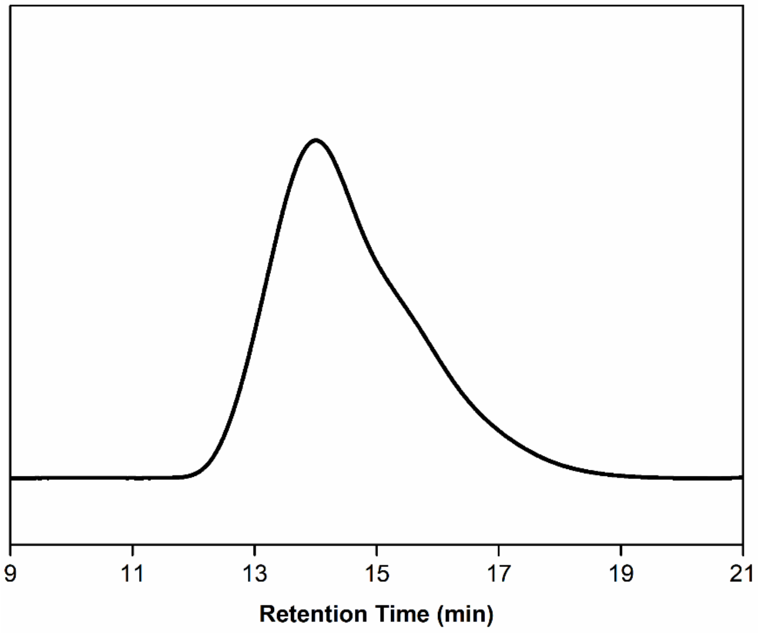

GPC chromatogram of the polymer of Table 3, entry 1

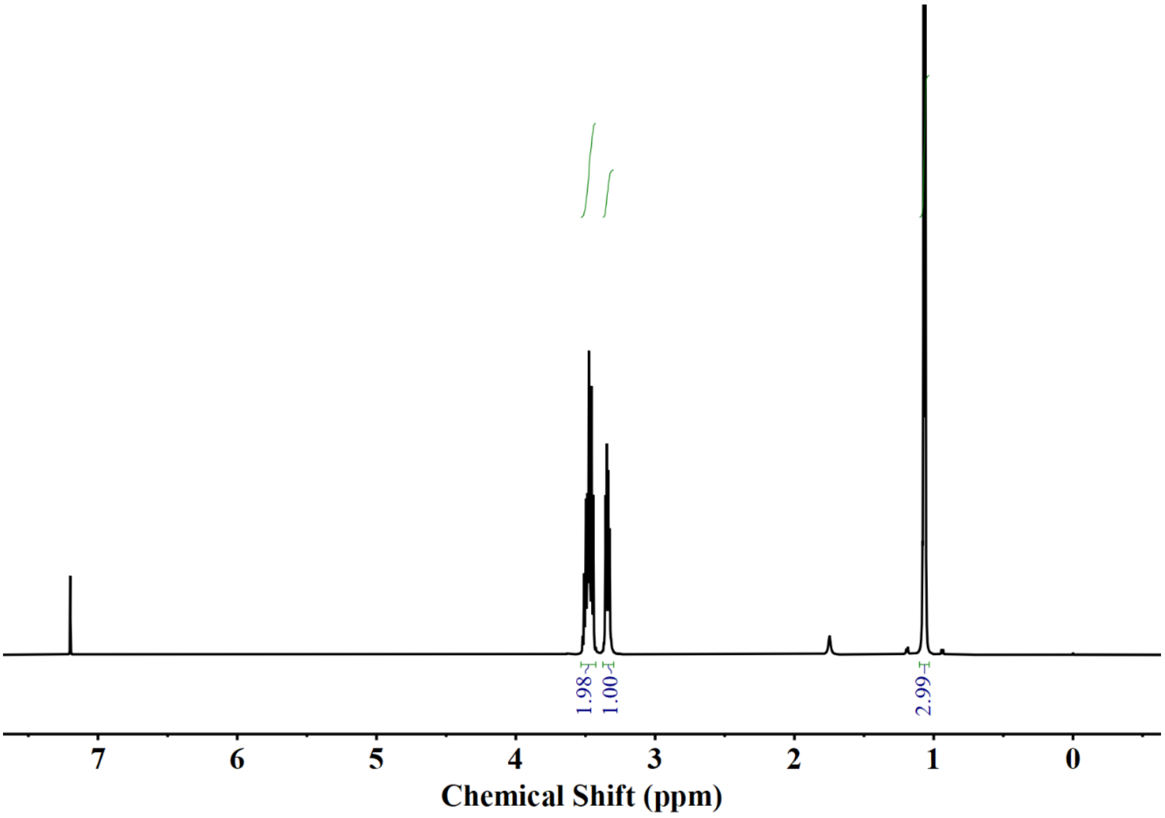

^{1}H NMR spectrum of the polymer of Table 3, entry 1

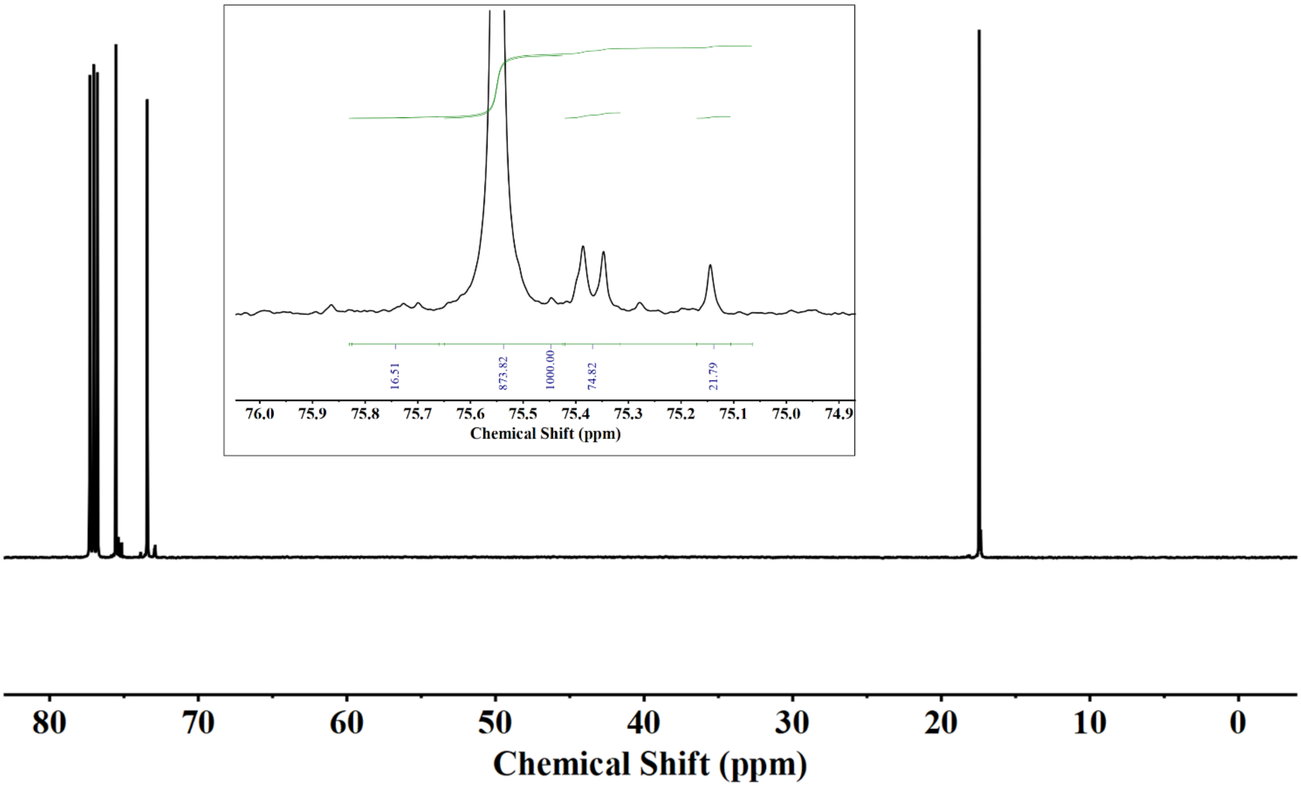

^{13}C NMR spectrum of the polymer of Table 3, entry 1

Table 3, entry 2:

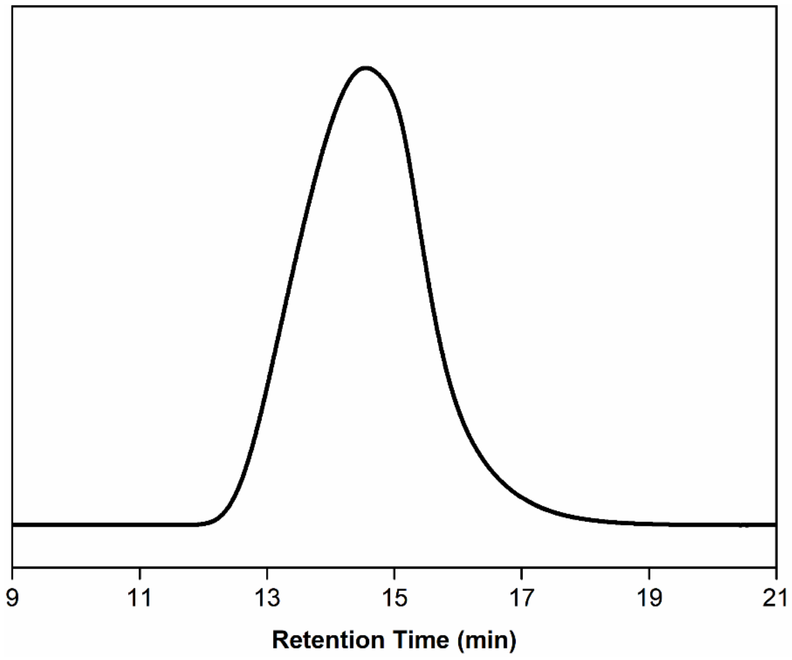

GPC chromatogram of the polymer of Table 3, entry 2

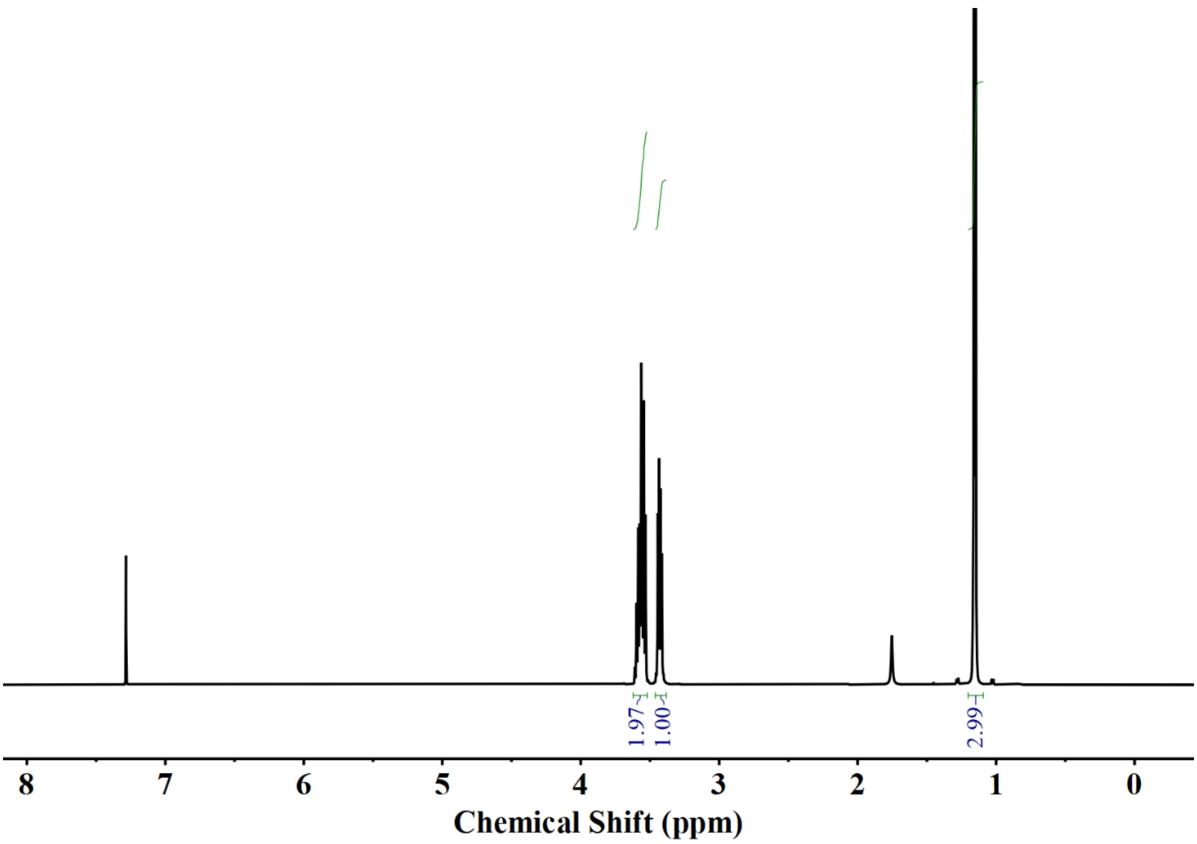

^{1}H NMR spectrum of the polymer of Table 3, entry 2

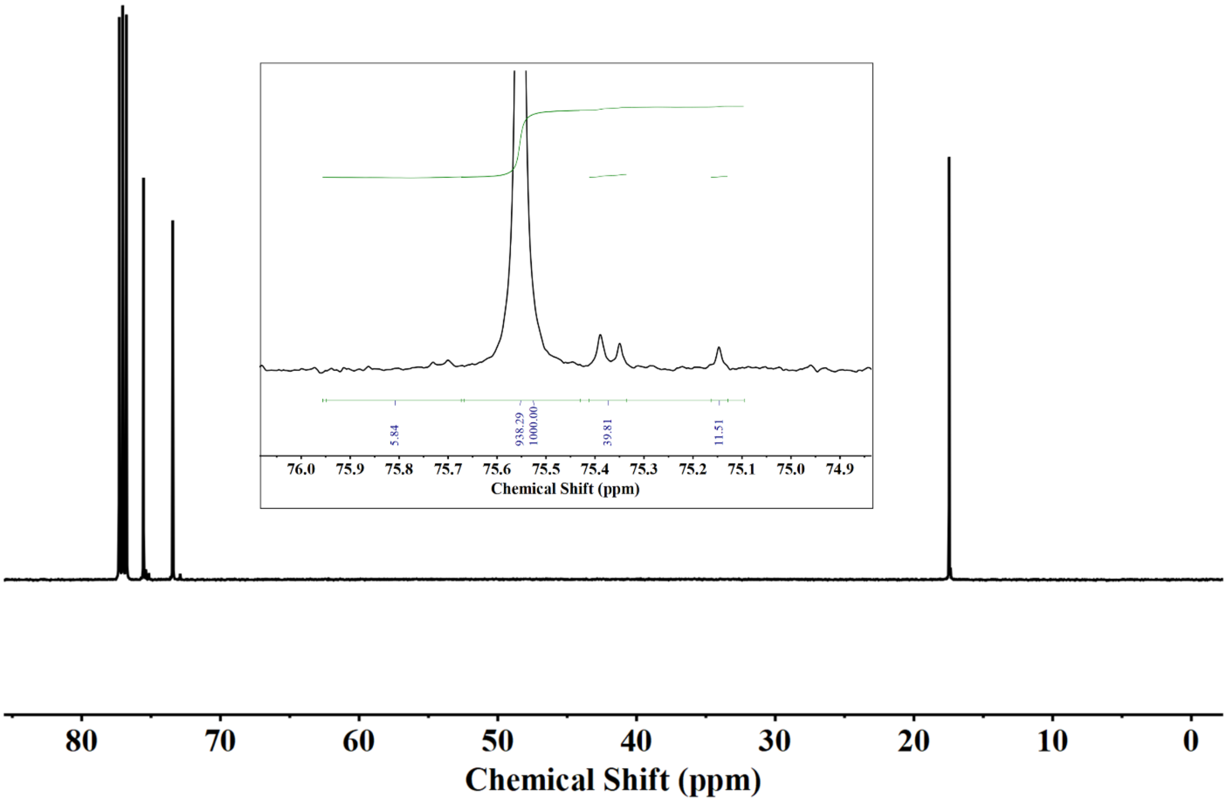

^{13}C NMR spectrum of the polymer of Table 3, entry 2

Table 3, entry 3:

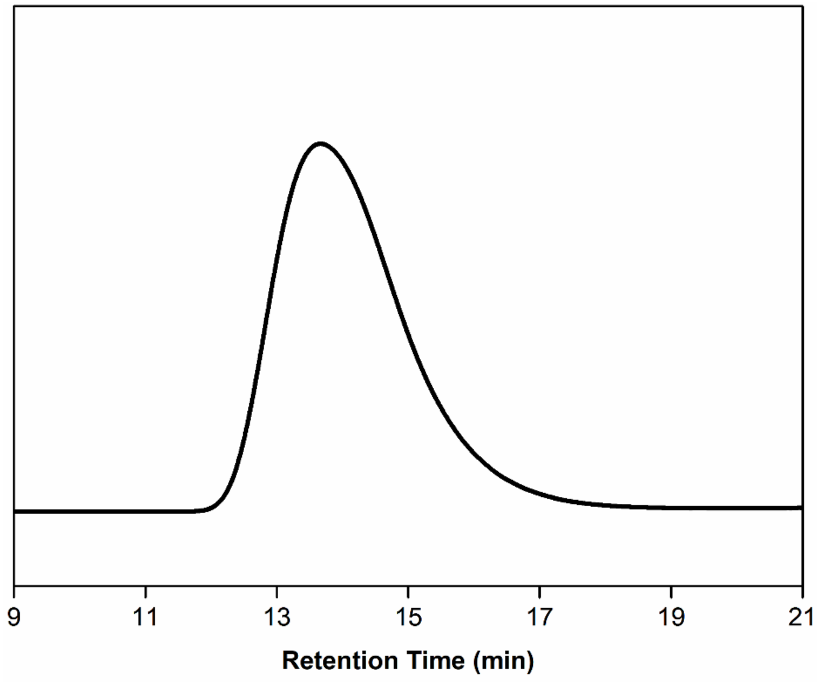

GPC chromatogram of the polymer of Table 3, entry 3

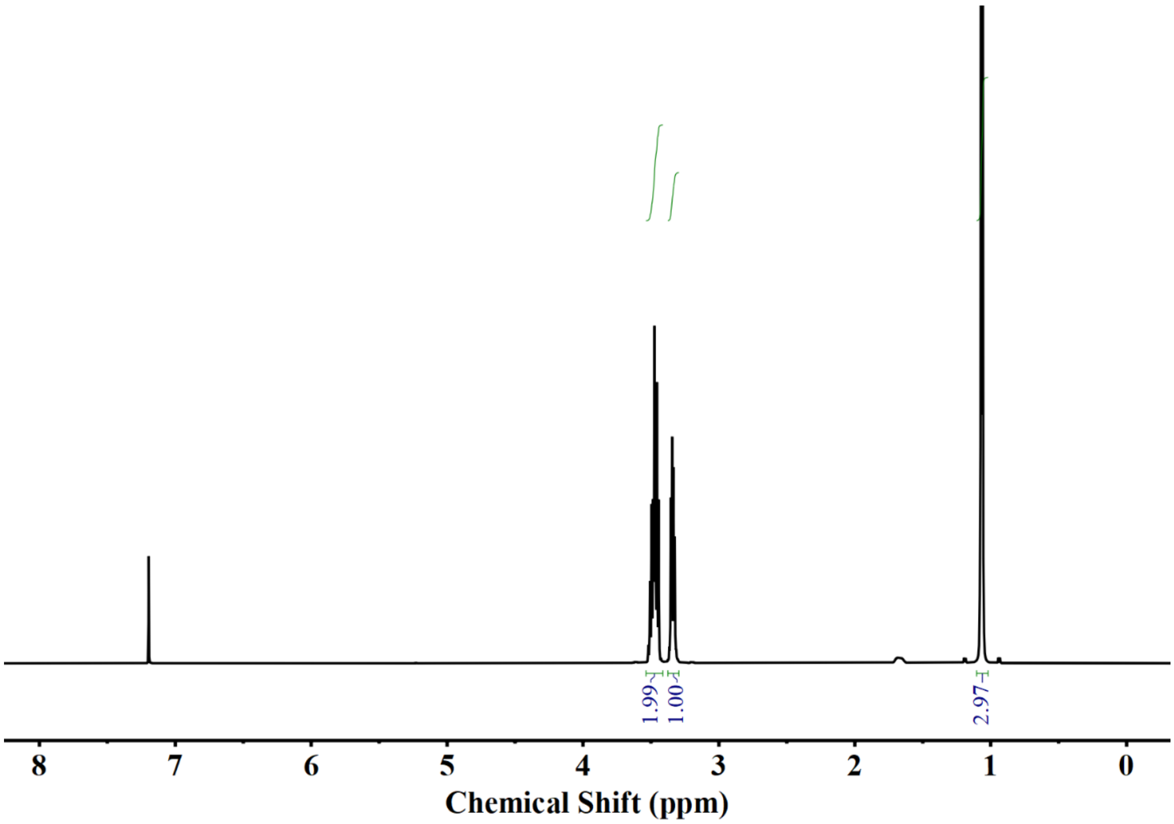

^{1}H NMR spectrum of the polymer of Table 3, entry 3

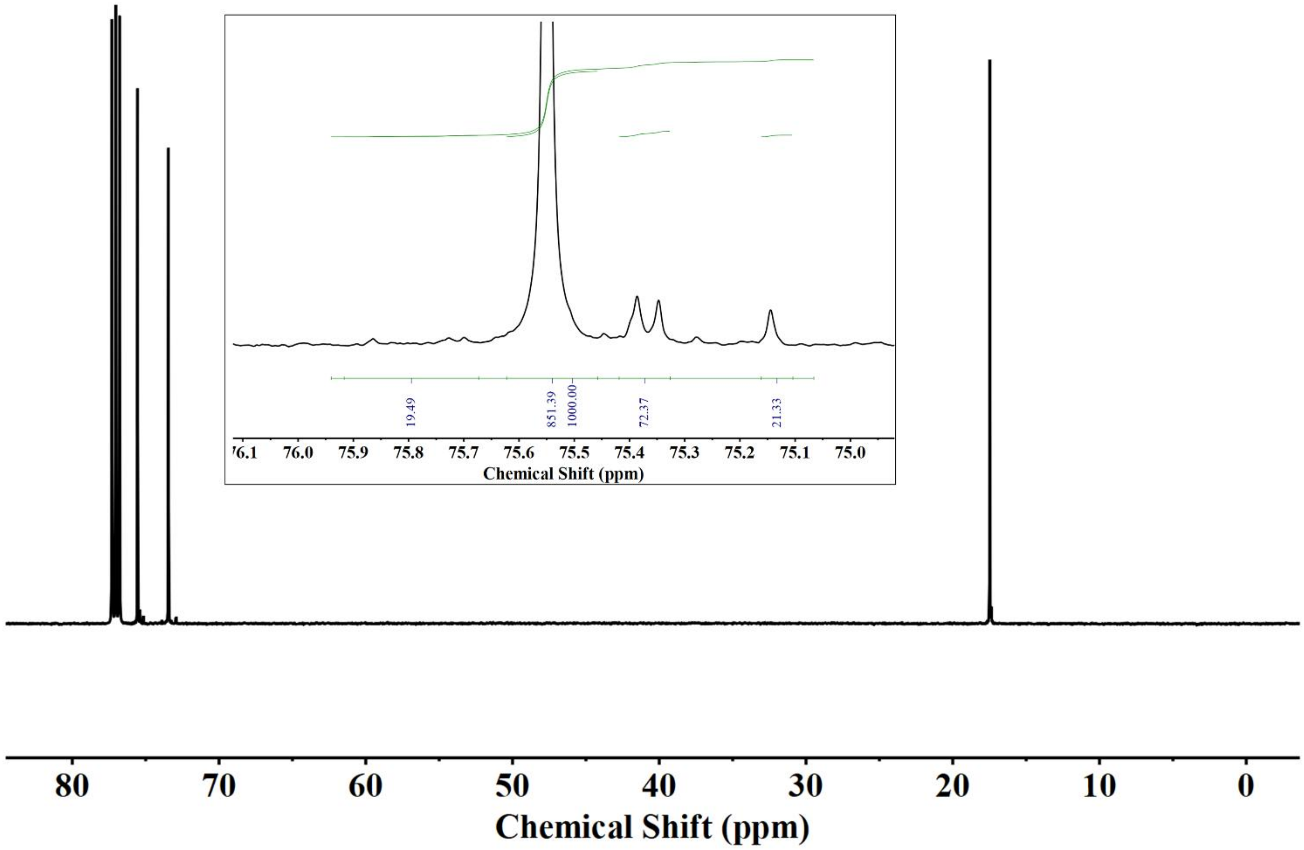

^{13}C NMR spectrum of the polymer of Table 3, entry 3

Table 3, entry 4:

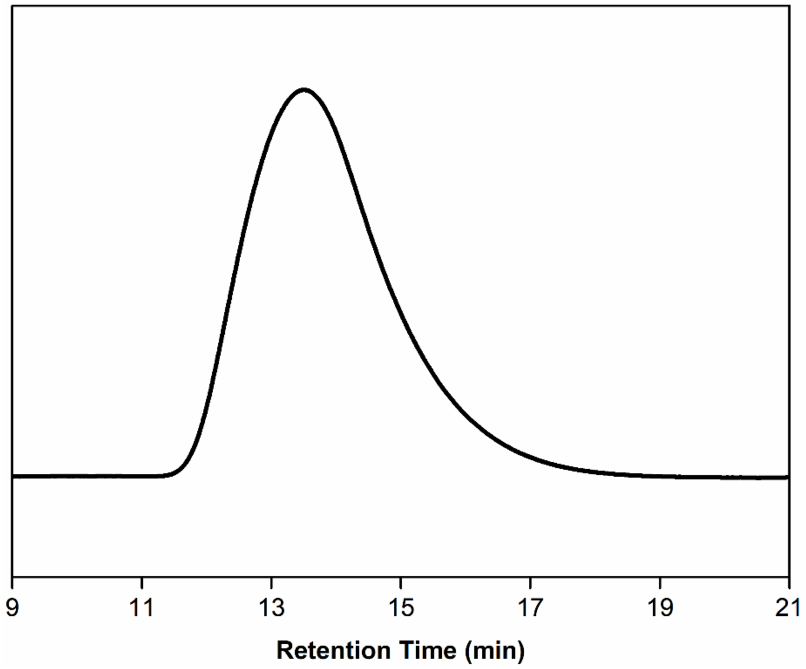

GPC chromatogram of the polymer of Table 3, entry 4

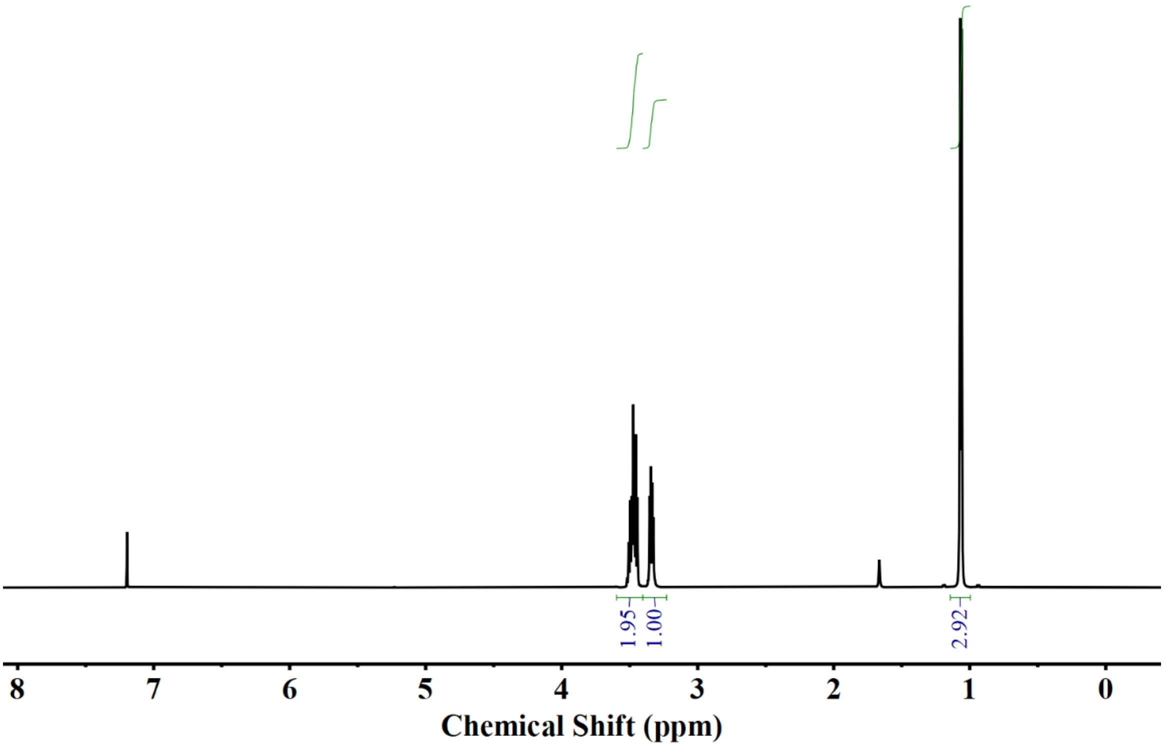

^{1}H NMR spectrum of the polymer of Table 3, entry 4

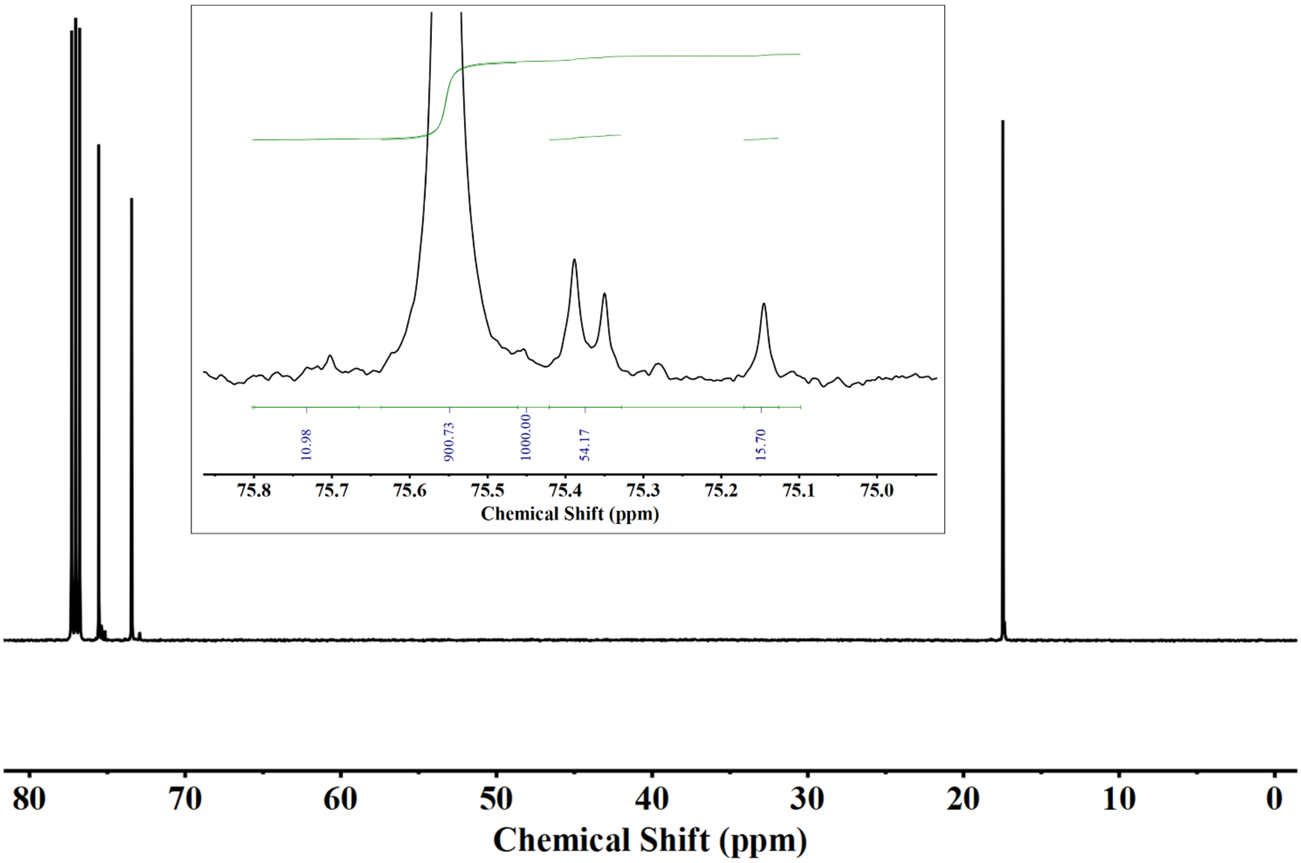

^{13}C NMR spectrum of the polymer of Table 3, entry 4

Table S2, Poly(1,2-Epoxybutane) (PBO):

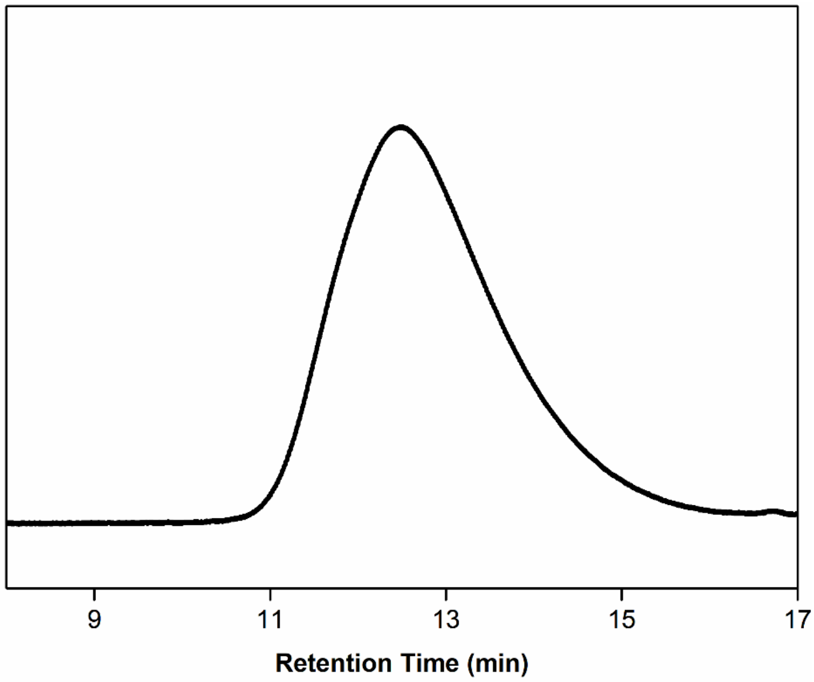

GPC chromatogram of PBO (Table S2)

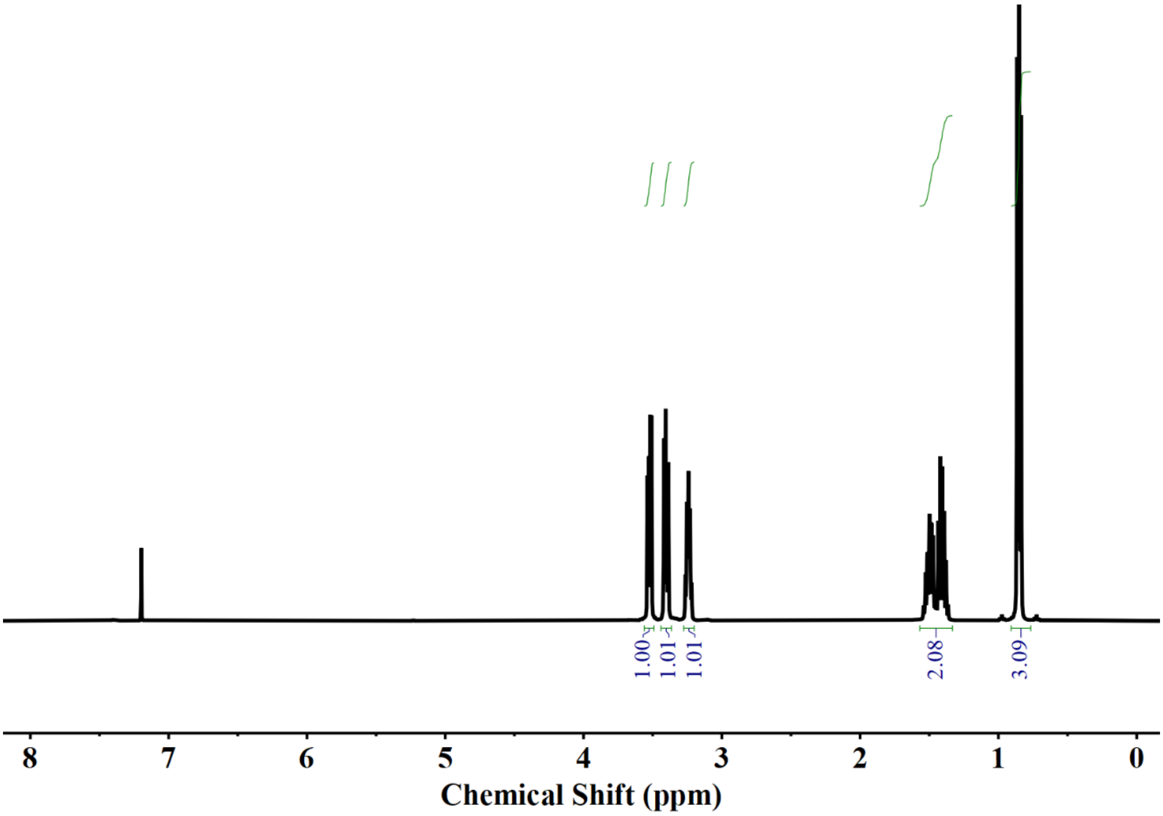

^1H NMR spectrum of PBO (Table S2)

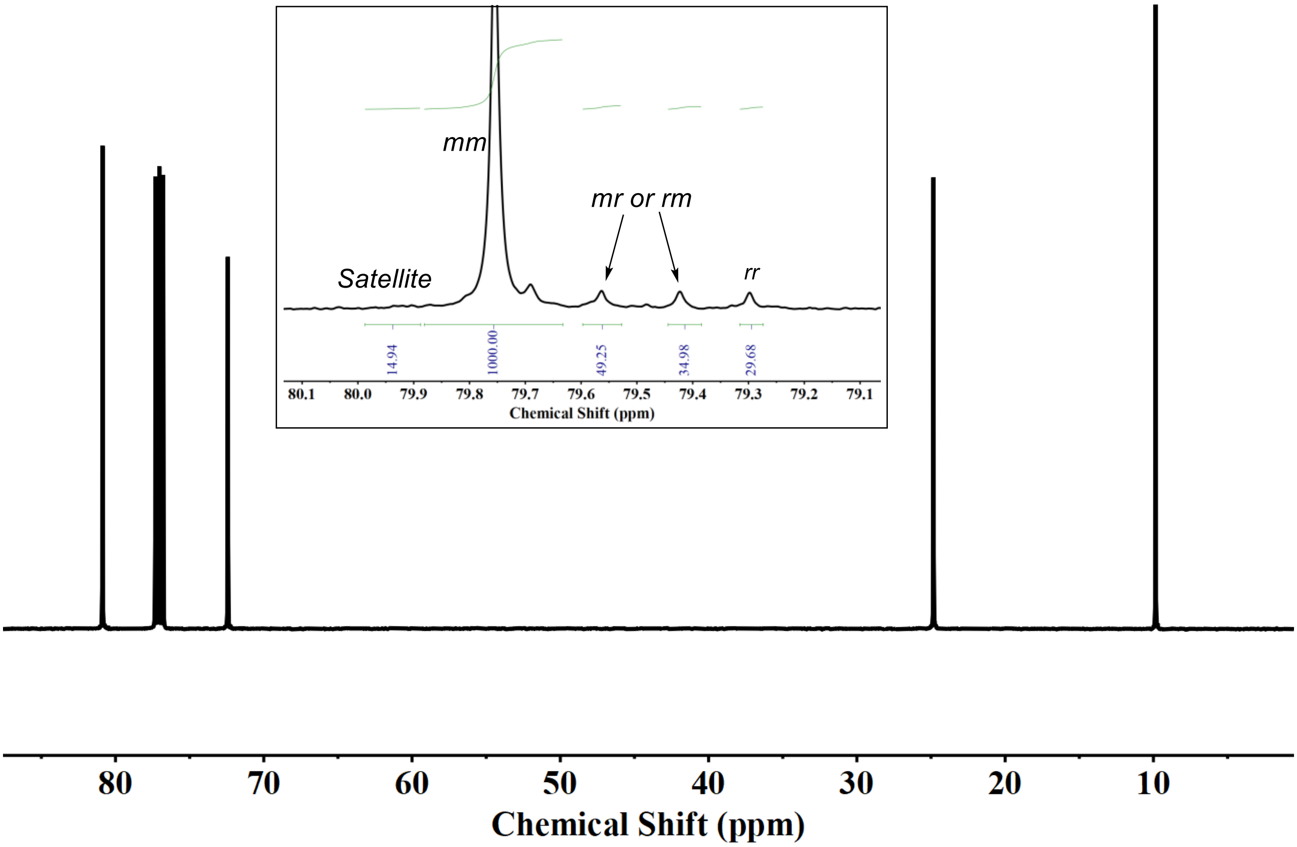

^{13}C NMR spectrum of PBO (Table S2)

Table S2, Poly(1,2-1,2-Epoxyhexane) (PHO):

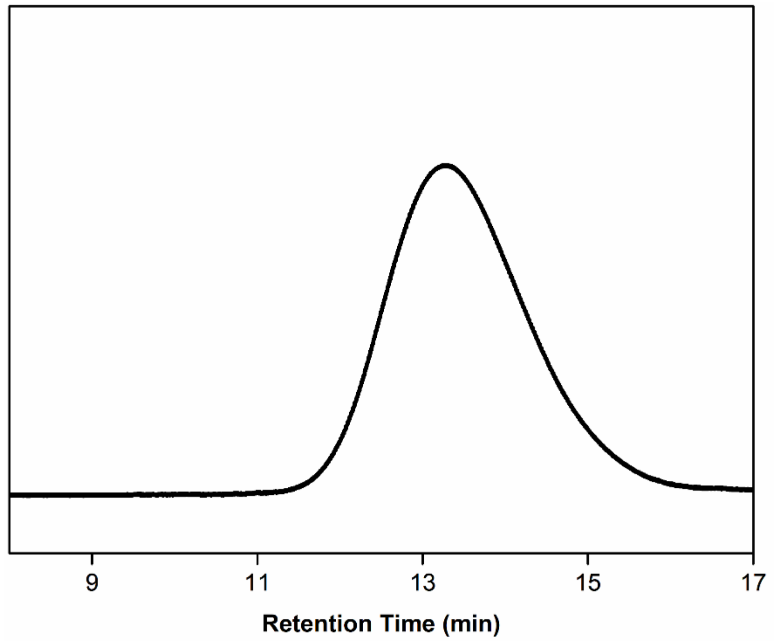

GPC chromatogram of PHO (Table S2)

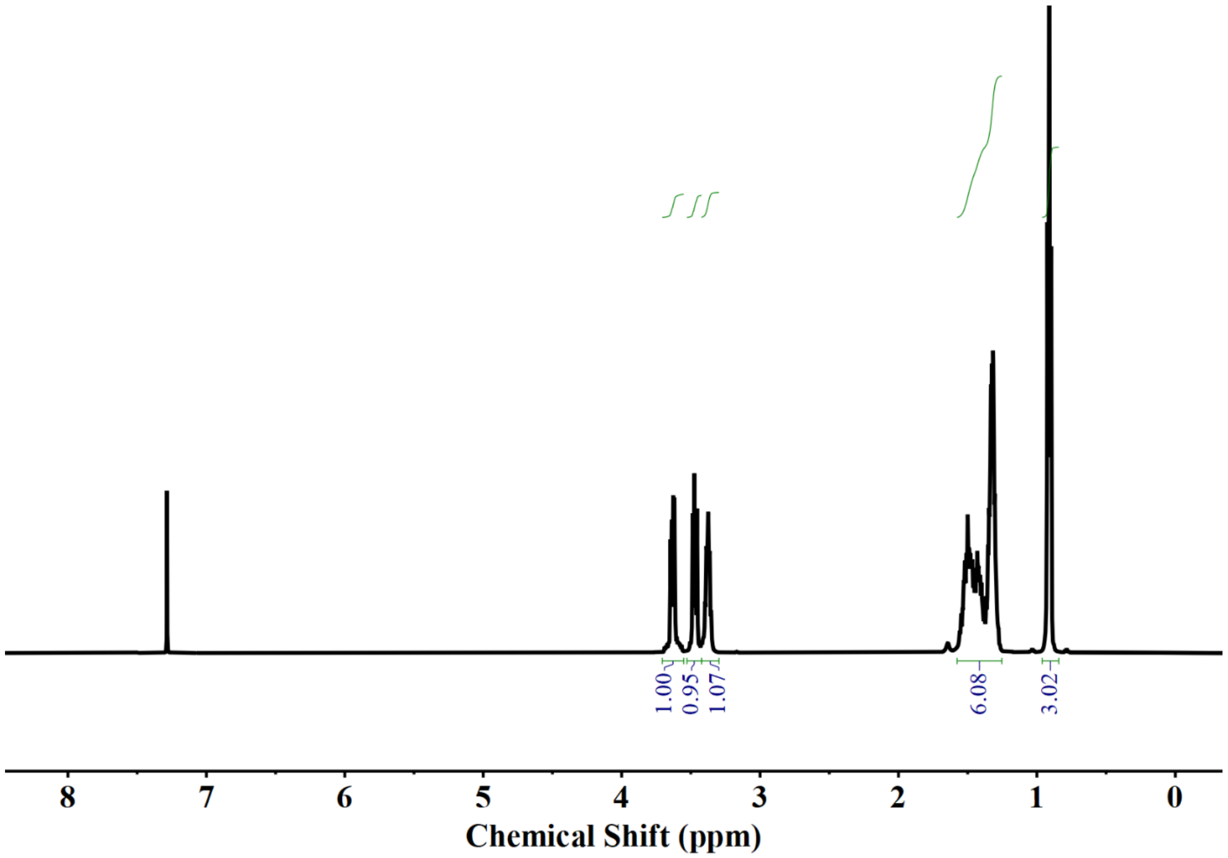

^{1}H NMR spectrum of PHO (Table S2)

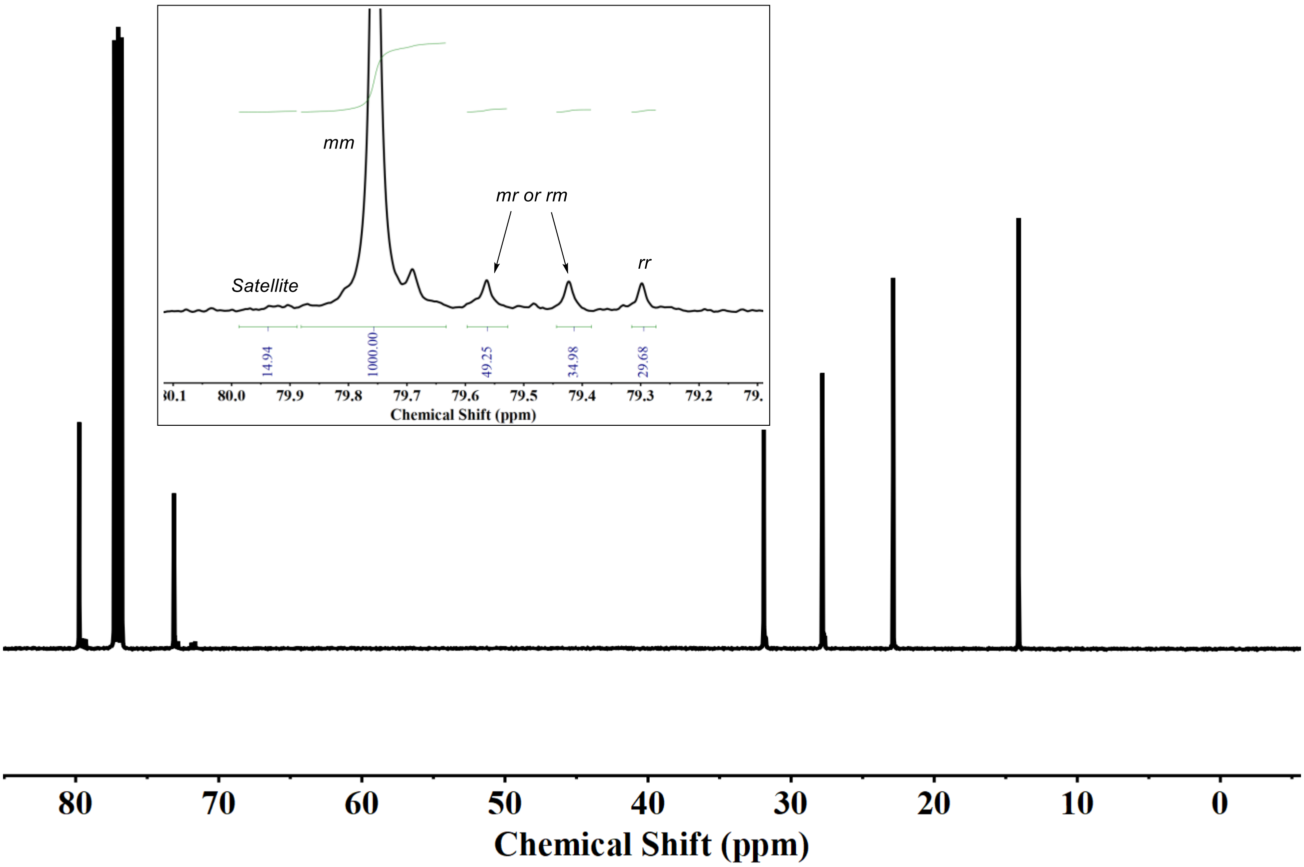

^{13}C NMR spectrum of PHO (Table S2)

7. Synthetic Procedures

The synthesis or purchase of all catalysts, ligands, and corresponding components are detailed in this section.

7.1 Diacids and Diacid Chlorides

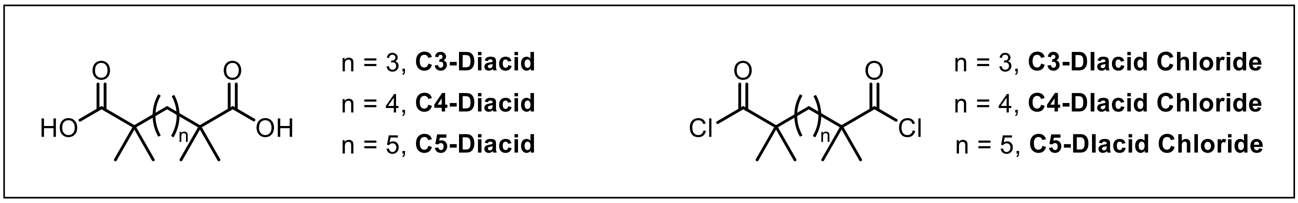

General Diacid Procedure:

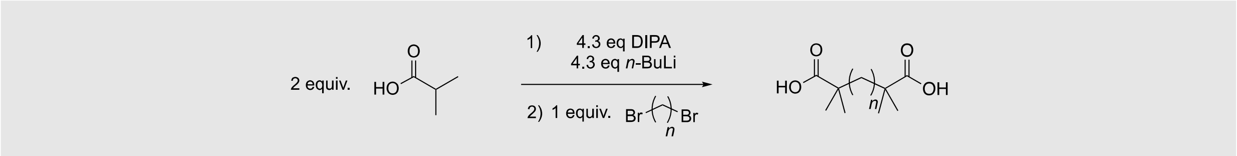

Minor adaptations were made to original published procedures of these diacids[5,6], diisopropylamine (DIPA, 4.3 equiv.) was added to THF using standard Schlenk technique. The reaction was cooled to 0 °C and 4.3 equiv. of *n*-BuLi (2.5 M solution in hexanes) was added dropwise. After stirring for 0.5 h, isobutyric acid (2 equiv.) was added dropwise. The reaction was kept at 0 °C for 1.0 h, then allowed to come to room temperature for 0.5 h. At this point, the reaction was cooled back to 0 °C and the dibromoalkane (1 equiv.) was added dropwise. The reaction was left to stir for 16 h, as it slowly came to room temperature. The reaction was quenched by cooling the flask and adding 1M HCl. The organic layer was isolated and the aqueous layer was extracted (3 times) with ethyl acetate. The organic layers were combined and solvent was removed by vacuum. The resulting solid was re-dissolved in MeOH, dried with Na_2SO_4, filtered, and the solvent was removed by vacuum. The resulting solid was collected on a glass frit and rinsed with DCM and pentanes. The remaining solvent was removed by vacuum. No further purification was necessary.

General Diacid Chloride Procedure:

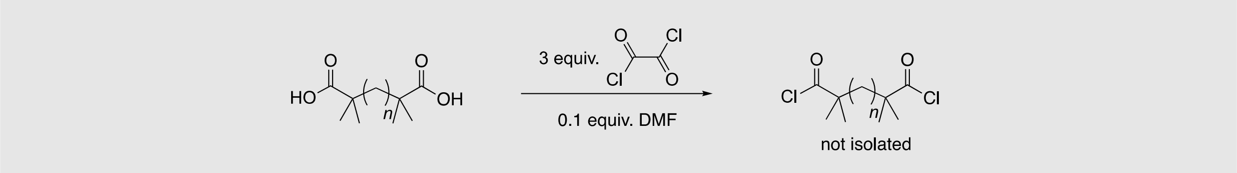

The corresponding diacid (1 equiv.) was added to an oven dried Schlenk flask and placed under an inert atmosphere. DCM and dimethylformamide (DMF, 0.1 equiv.) were added creating a heterogeneous mixture. The flask was cooled to 0 °C and oxalyl chloride (3 equiv.) was added dropwise by syringe. The ice bath was removed and the reaction was stirred until homogeneous (up to 16 h). Volatiles were removed by vacuum and the remaining contents were kept under an inert atmosphere and used without further purification.

C3-Diacid (2,2,6,6-tetramethylheptanedioic acid): Was synthesized via the ‘General Diacid Procedure” using 1,3-dibromopropane. The resulting product was colorless crystals isolated in 27% yield. Spectroscopic data matched the literature.[4]

C4-Diacid (2,2,7,7-tetramethyloctanedioic acid): Was synthesized via the ‘General Diacid Procedure” using 1,4-dibromobutane. The resulting product was colorless crystals isolated in 62% yield. Spectroscopic data matched the literature.[4]

C5-Diacid (2,2,8,8-tetramethylnonanedioic acid): Was synthesized via the ‘General Diacid Procedure” using 1,5-dibromopentane. The resulting product was colorless crystals isolated in 44% yield. Spectroscopic data matched the literature.[4]

C3-Diacid Chloride (2,2,6,6-tetramethylheptanedioyl dichloride): Was synthesized via the ‘General Diacid Chloride Procedure” using **C3-Diacid**. The resulting product was not isolated, but used as is for the synthesis of corresponding ligands.

C4-Diacid Chloride (2,2,7,7-tetramethyloctanedioyl dichloride): Was synthesized via the ‘General Diacid Chloride Procedure” using **C4-Diacid**. The resulting product was not isolated, but used as is for the synthesis of the corresponding ligand.

C5-Diacid Chloride (2,2,8,8-tetramethylnonanedioyl dichloride): Was synthesized via the ‘General Diacid Chloride Procedure” using **C5-Diacid**. The resulting product was not isolated, but used as is for the synthesis of the corresponding ligand.

7.2 Phenols

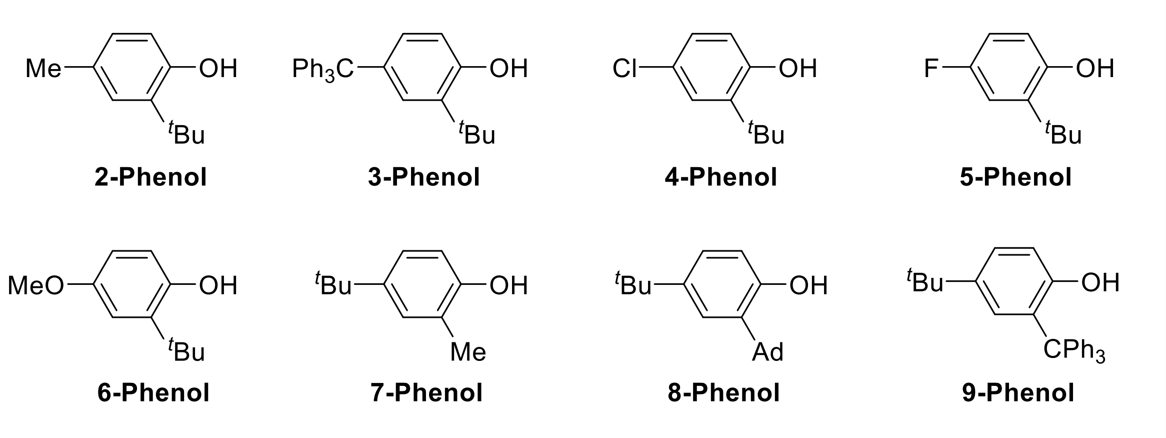

Phenols are listed below with the synthetic procedure or vendor source:

2-Phenol (2-(*tert*-butyl)-4-methylphenol): Was purchased from Sigma-Aldrich and used as received.

3-Phenol (2-(*tert*-butyl)-4-tritylphenol): Was synthesized as reported in the literature from 2-*tert*-butylphenol. Spectroscopic data matched the literature.[7]

4-Phenol (2-(*tert*-butyl)-4-chlorophenol): Was synthesized as reported in the literature from 4-chlorophenol. Spectroscopic data matched the literature.[8]

5-Phenol (2-(*tert*-butyl)-4-fluorophenol): Was synthesized as reported in the literature from 4-fluorophenol. Spectroscopic data matched the literature.[9]

6-Phenol (2-(*tert*-butyl)-4-methoxyphenol): Was purchased from Sigma-Aldrich and used as received.

7-Phenol (4-(*tert*-butyl)-2-methylphenol): Was purchased from Sigma-Aldrich and used as received.

8-Phenol (4-(*tert*-butyl)-2-adamantanylphenol): Was synthesized as reported in the literature from 4-*tert*-butylphenol. Spectroscopic data matched the literature.[10]

9-Phenol (4-(*tert*-butyl)-2-tritylphenol): Was synthesized as reported in the literature from 4-*tert*-butylphenol. Spectroscopic data matched the literature.[11]

7.3 Salicylaldehydes

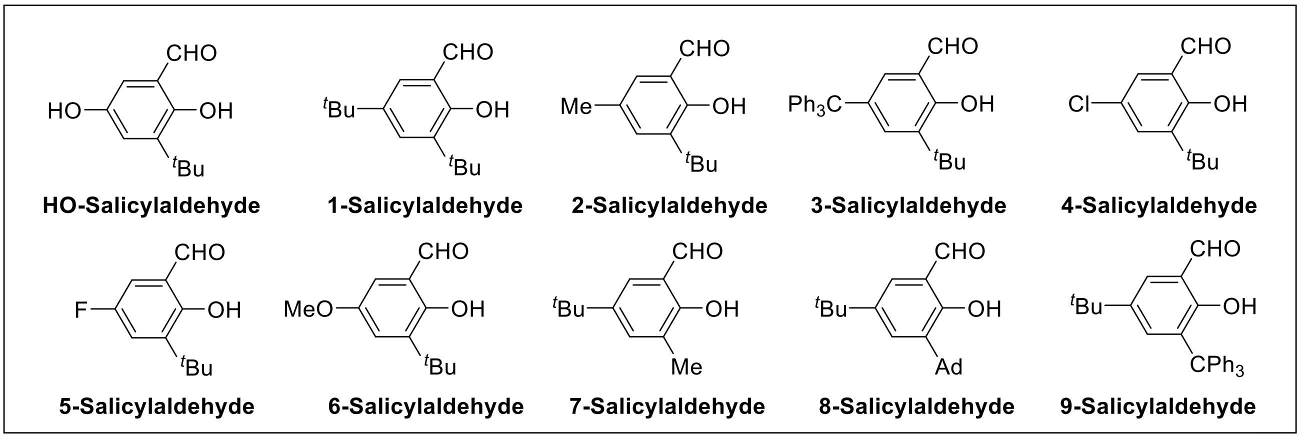

General Formylation Procedure:

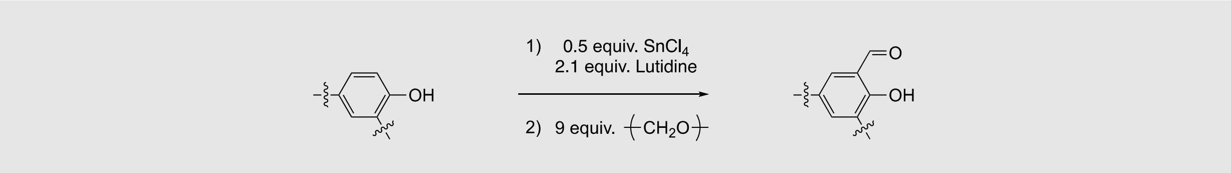

#-Phenol (1 equiv.) was added to an oversized Schlenk flask and placed under N_2 and dry, inert atmosphere. Toluene was added via cannula and the reaction was cooled to 0 °C. Lutidine (2.1 equiv.) and tin tetrachloride (0.5 equiv.) were added by syringe and the reaction was allowed to stir for 1 hour at room temperature. Paraformaldehyde (9 equiv.) was added against a positive pressure of N_2 gas. The reaction vessel was then sealed and heated to 90 °C to stir for 18 hours. The large headspace of the oversized flask removed the risk of over pressurizing, and therefore, the total liquid volume was kept below one-third of the flask capacity. The reaction was cooled to room temperature and filtered over Celite. Solvent was removed via rotary evaporation. The reaction mixture was redissolved in ethyl acetate before being washed with water (1×) and with 0.1 M HCl (1×). Solvent was removed via rotary evaporation and the reaction mixture was further purified by crystallization or column chromatography.

HO-Salicylaldehyde (3-(*tert*-butyl)-2,5-dihydroxybenzaldehyde): Was synthesized as reported in the literature. Spectroscopic data matched the literature.[12]

1-Salicylaldehyde (3,5-di-(*tert*-butyl)-2-hydroxybenzaldehyde): Was purchased from Sigma-Aldrich and used as received.

2-Salicylaldehyde (3-(*tert*-butyl)-2-hydroxy-5-methylbenzaldehyde): Was synthesized via the "General Formylation Procedure" using **2-Phenol**. Spectroscopic data matched the literature.[13]

3-Salicylaldehyde (3-(*tert*-butyl)-2-hydroxy-5-methylbenzaldehyde): Was synthesized via the "General Formylation Procedure" using **3-Phenol**. Spectroscopic data matched the literature.[14]

4-Salicylaldehyde (3-(*tert*-butyl)-5-chloro-2-hydroxybenzaldehyde): Was synthesized via the "General Formylation Procedure" using **4-Phenol**. Spectroscopic data matched the literature.[15]

5-Salicylaldehyde (3-(*tert*-butyl)-5-fluoro-2-hydroxybenzaldehyde): Was synthesized via the "General Formylation Procedure" using **5-Phenol**. Spectroscopic data matched the literature.[13]

6-Salicylaldehyde (3-(*tert*-butyl)-2-hydroxy-5-methoxybenzaldehyde): Was synthesized via the "General Formylation Procedure" using **6-Phenol**. Spectroscopic data matched the literature.[11]

7-Salicylaldehyde (5-(*tert*-butyl)-2-hydroxy-3-methylbenzaldehyde): Was synthesized via the "General Formylation Procedure" using **7-Phenol**. Spectroscopic data matched the literature.[16]

8-Salicylaldehyde (3-adamantanyl-5-(*tert*-butyl)-2-hydroxybenzaldehyde): Was synthesized via the "General Formylation Procedure" using **8-Phenol**. Spectroscopic data matched the literature.[8]

9-Salicylaldehyde (5-(*tert*-butyl)-2-hydroxy-3-tritylbenzaldehyde): Was synthesized via the "General Formylation Procedure" using **9-Phenol**. Spectroscopic data matched the literature.[17]

7.4 Salen Moieties

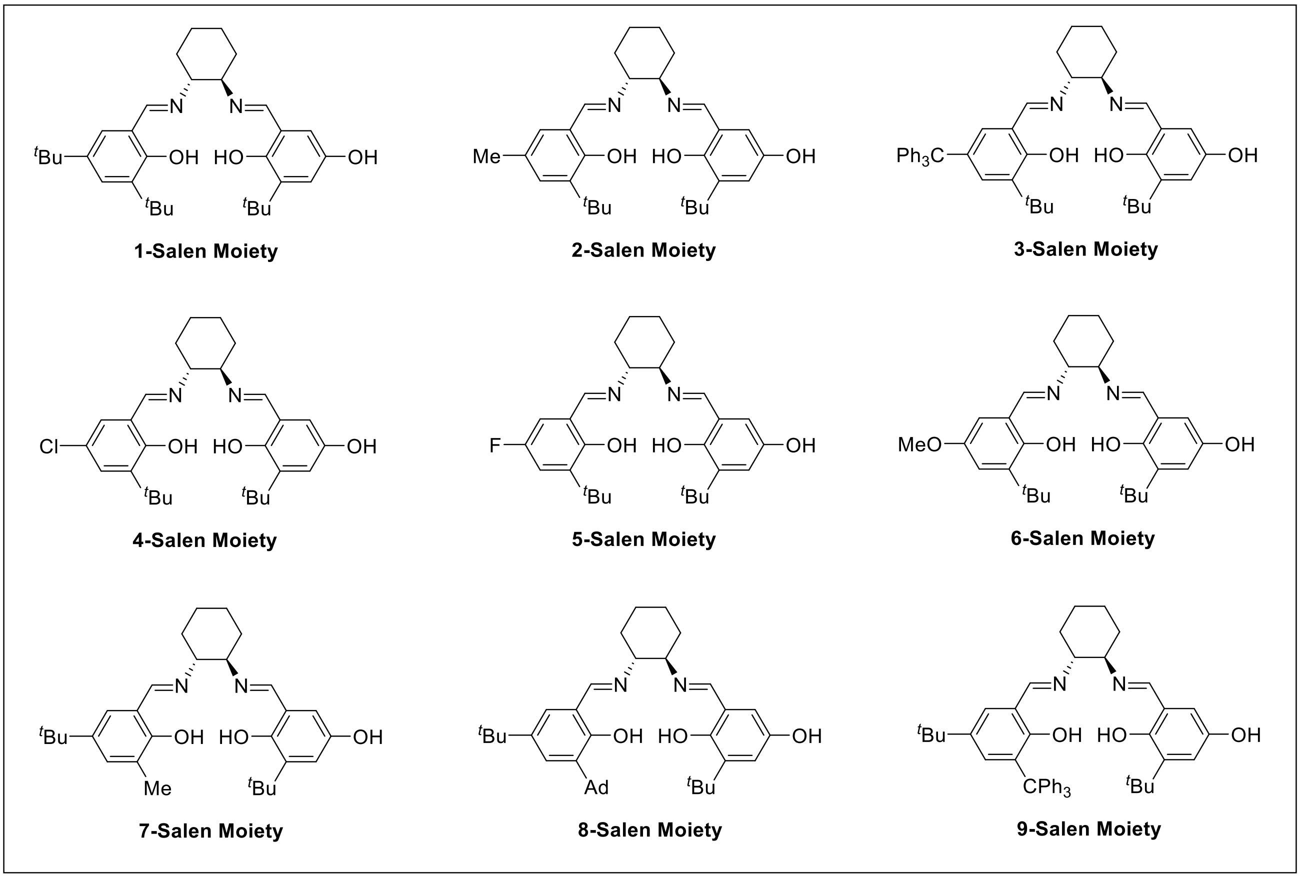

General Salen Moiety Procedure:

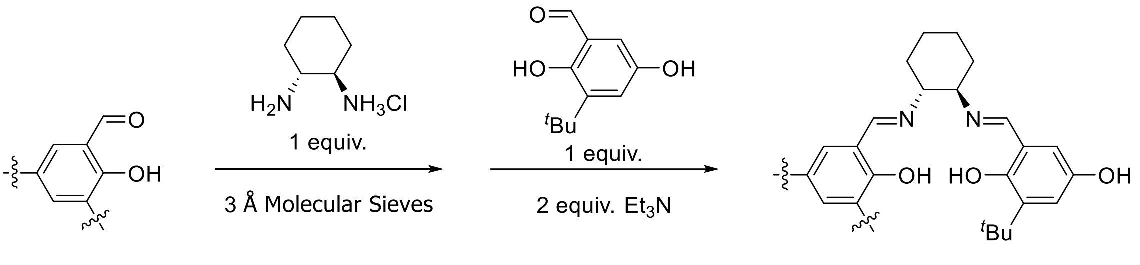

#-Salicylaldehyde (1 equiv., 2 mmol), (*R,R*)-diaminocyclohexanemonohydrochloride (1 equiv., 2 mmol, 300 mg), and 3 Å molecular sieves were added to an oven dried Schlenk flask and put under an inert atmosphere. Methanol (15 mL) was added via cannula and heterogeneous mixture was allowed to stir. **HO-Salicylaldehyde** (1 equiv., 2 mmol, 388 mg) was added to a separate Schlenk flask. Triethylamine (2 equiv., 4 mmol, 0.56 mL) and methanol were added by standard Schlenk technique and the homogeneous mixture was allowed to stir. After 30 minutes, the contents of the latter flask were transferred to the heterogeneous mixture using a cannula. The combined reaction was allowed to

stir for 1 hour at which point it was filtered over Celite and the solvent was removed by rotary evaporation. The crude mixture was dissolved in diethyl ether, washed with 1M HCl (2×) and saturated $NaHCO_3$ (1×) before being dried over Na_2SO_4. The mixture was filtered and the solvent was removed. The product was purified by flash chromatography.

1-Salen Moiety: Was synthesized via the "General Salen Moiety Procedure" using **1-Salicylaldehyde.** Spectroscopic data matched the literature.[18]

2-Salen Moiety: Was synthesized via the "General Salen Moiety Procedure" using **2-Salicylaldehyde.** Column conditions: 20% to 30% Et_2O in hexanes with 1% Et_3N. The resulting product was a yellow-orange foam (279 mg, 30% yield). ^{1}H NMR (500 MHz, $CDCl_3$) δ (ppm): 1.39 (s, 9H), 1.40 (s, 9H), 1.46 (m, 2H), 1.75 (m, 2H), 1.88 (m, 2H), 1.98 (m, 2H), 2.19 (s, 3H), 3.30 (m, 2H), 6.44 (d, J = 3.08 Hz, 1H), 6.78 (d, J = 1.56 Hz, 1H), 6.80 (d, J = 3.07 Hz, 1H), 7.05 (d, J = 1.82 Hz, 1H), 8.16 (s, 1H), 8.22 (s, 1H). ^{13}C NMR (126 MHz, $CDCl_3$) δ (ppm): 20.70, 24.43, 29.36, 29.53, 33.21, 33.23, 34.80, 34.97, 72.50, 72.60, 114.62, 117.96, 118.38, 118.40, 126.61, 129.84, 130.46, 136.92, 138.70, 146.71, 154.63, 158.13, 165.07, 165.70. HRMS (DART-MS) (m/z): Calculated for [**2-Salen Moiety**+1H]$^+$ ($[C_{29}H_{41}N_2O_3]^+$) = 465.3112; found = 465.3094

3-Salen Moiety: Was synthesized via the "General Salen Moiety Procedure" using **3-Salicylaldehyde**. Column conditions: 20% to 25% EtOAc in hexanes with 1% Et_3N. The resulting product was a yellow-orange foam (263 mg, 19% yield). ^{1}H NMR (500 MHz, $CDCl_3$) δ (ppm): 1.26 (s, 9H), 1.37 (s, 9H), 1.44 (m, 2H), 1.69 (m, 2H), 1.90 (m, 4H), 3.32 (m, 2H), 6.49 (d, J = 2.92 Hz, 1H), 6.84 (d, J = 2.25 Hz, 1H), 6.85 (d, J = 2.85 Hz, 1H), 7.07 (d, J = 2.12 Hz, 1H), 7.18 (m, 15H), 8.10 (s, 1H), 8.21 (s, 1H). ^{13}C NMR (126 MHz, $CDCl_3$) δ (ppm): 24.34, 24.40, 29.39, 29.48, 33.20, 33.38, 34.97, 64.48, 72.03, 72.63, 114.61, 117.35, 117.96, 118.44, 125.94, 127.51, 131.19, 133.85, 135.87, 136.06, 138.79, 146.72, 147.03, 154.64, 158.91, 165.03, 165.95. HRMS (DART-MS) (m/z): Calculated for [**3-Salen Moiety**+1H]$^+$ ($[C_{47}H_{53}N_2O_3]^+$) = 693.4051; found = 693.4045

4-Salen Moiety: Was synthesized via the "General Salen Moiety Procedure" using **4-Salicylaldehyde**. Column conditions: 20% to 30% EtOAc in hexanes. The resulting product was a yellow-orange foam (582 mg, 60% yield). ^{1}H NMR (500 MHz, $CDCl_3$) δ (ppm): 1.38 (s, 9H), 1.39 (s, 9H), 1.47 (m, 2H), 1.75 (m, 2H), 1.90 (m, 2H), 1.98 (m, 2H), 3.31 (m, 2H), 6.45 (d, J = 3.06 Hz, 2H), 6.82 (d, J = 3.02 Hz, 2H), 6.95 (d, J = 2.63 Hz, 2H), 7.17 (d, J = 2.62 Hz, 2H), 8.16 (s, 1H), 8.19 (s, 1H). ^{13}C NMR (126 MHz, $CDCl_3$) δ (ppm): 24.36, 24.38, 29.26, 29.35, 33.02, 33.15, 35.00, 35.14, 72.47, 72.54, 114.56, 118.16, 118.29, 119.29, 122.58, 128.69, 129.58, 138.86, 139.50, 146.77, 154.59,

159.12, 164.63, 165.17. HRMS (DART-MS) (m/z): Calculated for [**4-Salen Moiety**+1H]$^+$ ([$C_{28}H_{38}N_2O_3Cl$]$^+$) = 485.2571; found = 485.2572.

5-Salen Moiety: Was synthesized via the "General Salen Moiety Procedure" using **5-Salicylaldehyde**. Column conditions: 20% to 30% Et_2O in hexanes. The resulting product was a yellow-orange foam (394 mg, 42% yield). 1H NMR (500 MHz, $CDCl_3$) δ (ppm): 1.39 (s, 18H), 1.47 (m, 2H), 1.75 (m, 2H), 1.90 (m, 2H), 1.97 (m, 2H), 3.31 (m, 2H), 6.46 (d, J = 3.04 Hz, 1H), 6.67 (dd, J = 7.73 Hz, J = 3.07 Hz, 1H), 6.81 (d, J = 3.02 Hz, 1H), 6.99 (dd, J = 10.8 Hz, J = 3.10 Hz, 1H), 8.17 (s, 1H), 8.20 (s, 1H). ^{13}C NMR (125 MHz, $CDCl_3$) δ (ppm): 24.37, 24.39, 29.24, 29.35, 33.09, 33.17, 34.99, 35.09, 72.50, 72.59, 114.12, 114.30, 114.56, 116.95, 117.14, 118.10, 118.15, 118.32, 138.82, 139.36, 139.41, 146.76, 154.00, 154.59, 155.87, 156.54, 164.75, 165.14. HRMS (DART-MS) (m/z): Calculated for [**5-Salen Moiety**+1H]$^+$ ([$C_{28}H_{38}N_2O_3F$]$^+$) = 469.2861; found = 469.2865.

6-Salen Moiety: Was synthesized via the "General Salen Moiety Procedure" using **6-Salicylaldehyde**. Column conditions: 20% to 60% Et_2O in hexanes with 1% Et_3N. The resulting product was a yellow-orange foam (471 mg, 49% yield). 1H NMR (500 MHz, $CDCl_3$) δ (ppm): 1.38 (s, 9H), 1.39 (s, 9H), 1.46 (m, 2H), 1.74 (m, 2H), 1.89 (m, 2H), 1.96 (m, 2H), 3.30 (m, 2H), 3.68 (s, 3H), 6.44 (d, J = 3.02 Hz, 1H), 6.47 (d, J = 3.07 Hz, 1H), 6.81 (d, J = 3.16 Hz, 1H), 6.90 (d, J = 3.14 Hz, 1H), 8.16 (s, 1H), 8.23 (s, 1H). ^{13}C NMR (125 MHz, $CDCl_3$) δ (ppm): 24.41, 29.37, 29.40, 33.17, 33.24, 34.97, 35.05, 55.83, 72.50, 72.57, 111.52, 114.64, 117.96, 118.02, 118.37, 118.40, 138.67, 138.88, 146.80, 151.22, 154.58, 154.98, 165.15, 165.48. HRMS (DART-MS) (m/z): Calculated for [**6-Salen Moiety**+1H]$^+$ ([$C_{29}H_{41}N_2O_4$]$^+$) = 481.3061; found = 481.3046.

7-Salen Moiety: Was synthesized via the "General Salen Moiety Procedure" using **7-Salicylaldehyde**. Column conditions: 15% to 20% EtOAc in hexanes with 1% Et_3N. The resulting product was a yellow-orange foam (279 mg, 30% yield). ^{1}H NMR (500 MHz, $CDCl_3$) δ (ppm): 1.22 (s, 9H), 1.37 (s, 9H), 1.45 (m, 2H), 1.71 (m, 2H), 1.86 (m, 2H), 1.93 (m, 2H), 2.23 (s, 3H), 3.25 (m, 1H), 3.32 (m, 1H), 6.42 (d, J = 3.01 Hz, 1H), 6.80 (d, J = 3.03 Hz, 1H), 6.96 (d, J = 2.49 Hz, 1H), 7.17 (d, J = 2.39 Hz, 1H), 8.12 (s, 1H), 8.26 (s, 1H). ^{13}C NMR (125 MHz, $CDCl_3$) δ (ppm): 15.87, 24.38, 29.37, 31.51, 33.24, 33.30, 33.92, 34.96, 72.52, 72.70, 114.65, 117.26, 118.03, 118.36, 125.26, 125.52, 130.97, 138.70, 140.83, 146.82, 154.57, 157.36, 164.97, 165.54. HRMS (DART-MS) (m/z): Calculated for [**7-Salen Moiety**+1H]$^+$ ([$C_{29}H_{41}N_2O_3$]$^+$) = 465.3112; found = 465.3112.

8-Salen Moiety: Was synthesized via the "General Salen Moiety Procedure" using **8-Salicylaldehyde**. Column conditions: 50% to 80% DCM in hexanes. The resulting product was a yellow-orange foam (632 mg, 54% yield). ^{1}H NMR (500 MHz, $CDCl_3$) δ (ppm): 1.23 (s, 9H), 1.38 (s,

9H), 1.45, (m, 2H), 1.80 (m, 8H), 1.88 (m, 2H), 1.95 (m, 2H), 2.08 (m, 3H), 2.15 (m, 6H), 3.31 (m, 2H), 6.46 (d, J = 2.88 Hz, 1H), 6.80 (d, J = 3.05 Hz, 1H), 6.95 (d, J = 2.38 Hz, 1H), 7.25 (d, J = 2.47 Hz, 1H), 8.17 (s, 1H), 8.28 (s, 1H). ^{13}C NMR (125 MHz, $CDCl_3$) δ (ppm): 24.46, 29.27, 29.38, 31.56, 33.22, 33.37, 34.23, 34.97, 37.31, 37.33, 40.45, 72.46, 72.56, 114.68, 117.89, 117.94, 118.43, 126.02, 126.98, 136.81, 138.69, 140.16, 146.69, 154.68, 158.40, 165.07, 166.19. HRMS (DART-MS) (m/z): Calculated for [**8-Salen Moiety**+1H]$^+$ ($[C_{38}H_{53}N_2O_3]^+$) = 585.4051; found = 585.4040.

9-Salen Moiety: Was synthesized via the "General Salen Moiety Procedure" using **9-Salicylaldehyde**. Column conditions: 20% to 25% EtOAc in hexanes with 1% Et_3N. The resulting product was a yellow-orange foam (624 mg, 45% yield). ^{1}H NMR (500 MHz, $CDCl_3$) δ (ppm): 1.10 (s, 9H), 1.39 (s, 9H), 1.64 (m, 4H), 1.84 (m, 4H), 3.16 (m, 2H), 6.24 (d, J = 3.16 Hz, 1H), 6.82 (d, J = 3.04 Hz, 1H), 7.03 (d, J = 2.43 Hz, 1H), 7.17 (m, 4H), 7.20 (m, 7H), 7.21 (m, 4H), 7.26 (m, 1H), 7.88 (s, 1H), 8.19 (s, 1H). ^{13}C NMR (125 MHz, $CDCl_3$) δ (ppm): 24.35, 24.43, 29.40, 31.35, 33.00, 33.03, 34.11, 34.96, 63.49, 72.22, 72.60, 114.77, 117.84, 118.03, 118.36, 125.60, 127.08, 127.24, 131.12, 131.80, 133.82, 138.51, 139.79, 145.82, 146.77, 154.47, 157.79, 165.12, 165.56. HRMS (DART-MS) (m/z): Calculated for [**9-Salen Moiety**+1H]$^+$ ($[C_{47}H_{53}N_2O_3]^+$) = 693.4051; found = 693.4052.

7.5 Ligands

General Ligand Procedure:

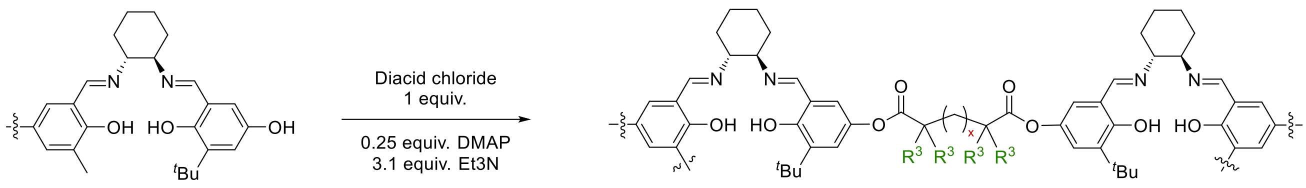

#-Salen moiety (2 equiv., 1 mmol) and DMAP (0.25 equiv., 0.13 mmol, 16 mg) were combined in an oven dried flask and place under an inert atmosphere. Et_3N (3.1 equiv**., 1.56 mmol, 0.22 mL**) and DCM (15 mL) were added by standard Schlenk technique. The reaction was cooled using an ice bath and allowed to stir. In a second dry flask, **#-diacid** chloride (1 equiv., 0.5 mmol) was added and DCM were combined under an inert atmosphere. The diacid chloride solution was transferred to the cooled reaction flask by cannula. The reaction was allowed to come to room temperature and stir for ~16 h. The solution was then diluted with additional DCM and washed with 1M HCl (2×), brine (1×), and saturated $NaHCO_3$ (1×). The remaining organics were dried over Na_2SO_4, filtered, and the solvent was removed by vacuum. The product was purified by flash chromatography or silica plug.

1-Ligand: Was synthesized via the "General Salen Moiety Procedure" using **1-Salen Moiety** and suberoyl chloride. Spectroscopic data matched the literature.[16]

2-Ligand: Was synthesized via the "General Salen Ligand Procedure" using **2-Salen Moiety** and suberoyl chloride. Silica plug: 20% Et_2O in hexanes with 1% Et_3N. The resulting product was a yellow powder (288 mg, 54% yield). ^1H NMR (500 MHz, $CDCl_3$) δ (ppm): 1.40 (s, 36H), 1.46 (m, 8H), 1.75 (m, 8H), 1.87 (m, 4H), 1.96 (m, 4H), 2.20 (s, 6H), 2.51 (t, J = 7.34, 4H), 3.32 (m, 4H), 6.75 (d, J = 2.60 Hz, 2H), 6.80 (d, J = 1.36 Hz, 2H), 6.93 (d, J = 2.60 Hz , 2H), 7.05 (d, J = 1.52 Hz, 2H), 8.22 (s, 2H), 8.25 (s, 2H), 13.54 (s, 2H), 13.83 (s, 2H). ^{13}C NMR (125 MHz, $CDCl_3$) δ (ppm): 20.69, 24.39, 24.41, 24.85, 28.88, 29.27, 29.55, 33.16, 33.25, 34.33, 34.78, 35.03, 72.31, 72.59, 118.29, 118.39, 121.47, 122.92, 126.64, 129.85, 130.51, 136.91, 138.67, 141.69, 158.08, 158.19, 164.84, 165.69, 172.63. HRMS (DART-MS) (m/z): Calculated for [**2-Ligand**+1H]$^+$ ([C66H91N4O8]+) = 1067.6831; found = 1067.6781.

3-Ligand: Was synthesized via the "General Salen Ligand Procedure" using **3-Salen Moiety** and suberoyl chloride. Silica plug: 50% Et_2O in hexanes. The resulting product was a yellow powder (556

mg, 73% yield). ^1H NMR (500 MHz, $CDCl_3$) δ (ppm): 1.25 (s, 18H), 1.36 (s, 18H), 1.48 (m, 8H), 1.70 (m, 4H), 1.77 (m, 4H), 1.89 (m, 8H), 2.54 (t, J = 7.49, 4H), 3.31 (m, 4H), 6.80 (d, J = 2.54 Hz, 2H), 6.85 (d, J = 2.14 Hz, 2H), 6.97 (d, J = 2.6 Hz, 2H), 7.07 (d, J = 2.17 Hz, 2H), 7.18 (m, 30H), 8.13 (s, 2H), 8.26 (s, 2H), 13.79 (s, 2H). ^{13}C NMR (125 MHz, $CDCl_3$) δ (ppm): 24.34, 24.37, 24.87, 28.91, 29.28, 29.49, 33.29, 33.34, 34.34, 34.95, 35.02, 64.49, 72.33, 72.45, 117.40, 118.37, 121.51, 122.95, 125.91, 127.50, 131.21, 131.29, 133.83, 135.94, 136.01, 138.77, 141.63, 147.04, 158.25, 158.73, 164.73, 165.91, 172.76. HRMS (DART-MS) (m/z): Calculated for [**3-Ligand**+1H]$^+$ ([$C_{102}H_{115}N_4O_8$]$^+$) = 1523.8709; found = 1523.8665.

4-Ligand: Was synthesized via the "General Salen Ligand Procedure" using **4-Salen Moiety** and suberoyl chloride. Silica plug: 50% Et_2O in hexanes. The resulting product was a yellow powder (470 mg, 85% yield). ^1H NMR (500 MHz, $CDCl_3$) δ (ppm): 1.38 (s, 18H), 1.39 (s, 18H), 1.45 (m, 8H), 1.74 (m, 8H), 1.93 (m, 8H), 2.51 (t, J = 7.59 Hz, 4H), 3.33 (m, 4H), 6.75 (d, J = 2.65 Hz, 2H), 6.93 (d, J = 2.65 Hz, 2H), 6.98 (d, J = 2.53 Hz, 2H), 7.18 (d, J = 2.52 Hz, 2H), 8.21 (s, 2H), 8.22 (s, 2H). ^{13}C NMR (125 MHz, $CDCl_3$) δ (ppm): 24.32, 24.37, 24.84, 28.88, 29.25, 29.27, 33.00, 33.19, 34.33, 35.06, 35.13, 72.17, 72.67, 118.20, 119.30, 121.44, 121.65, 123.09, 128.72, 129.63, 138.85, 139.48, 141.71, 158.15, 159.05, 164.60, 164.92, 172.69. HRMS (DART-MS) (m/z): Calculated for [**4-Ligand**+1H]$^+$ ([$C_{64}H_{85}N_4O_8Cl_2$]$^+$) = 1107.5739; found = 1107.5726.

5-Ligand: Was synthesized via the "General Salen Ligand Procedure" using **5-Salen Moiety** and suberoyl chloride. Silica plug: 50% Et_2O in hexanes. The resulting product was a yellow powder (490 mg, 91% yield). ^1H NMR (500 MHz, $CDCl_3$) δ (ppm): 1.38 (s, 36 H), 1.44 (m, 8H), 1.74 (m, 8H), 1.93 (m, 8H), 2.51 (t, J = 7.50 Hz, 4H), 3.33 (m, 4H), 6.70 (dd, J = 3.15 Hz, 7.99 Hz, 2H), 6.75 (d, J = 2.8 Hz, 2H), 6.92 (d, J = 2.75 Hz, 2H), 6.99 (dd, J = 3.05 Hz, 10.80 Hz, 2H), 8.21 (s, 2H), 8.23 (s, 2H). ^{13}C NMR (125 MHz, $CDCl_3$) δ (ppm): 24.32, 24.37, 24.84, 28.88, 29.25, 33.06, 33.21, 34.32, 35.04, 35.08, 72.21, 72.70, 114.14, 114.32, 117.00, 117.19, 188.08, 118.14, 118.22, 121.45, 123.05, 138.82, 139.36, 139.40, 141.69, 154.04, 155.91, 156.51, 158.16, 164.71, 164.74, 164.90, 172.68. HRMS (DART-MS) (m/z): Calculated for [**5-Ligand**+1H]$^+$ ([$C_{64}H_{85}N_4O_8F_2$]$^+$) = 1075.6330; found = 1075.6306.

6-Ligand: Was synthesized via the "General Salen Ligand Procedure" using **6-Salen Moiety** and suberoyl chloride. Silica plug: 25% Et_2O in hexanes with 1% Et_3N. The resulting product was a yellow powder (247 mg, 45% yield). ^1H NMR (500 MHz, $CDCl_3$) δ (ppm): 1.39 (s, 36H), 1.46 (m, 8H), 1.74 (m, 8H), 1.87 (m, 4H), 1.96 (m, 4H), 2.51 (t, J = 7.42 Hz, 4H), 3.32 (m, 4H), 3.69 (s, 6H), 6.49 (d, J = 2.81 Hz, 2H), 6.75 (d, J = 2.57 Hz, 2H), 6.90 (d, J = 2.82 Hz, 2H), 6.92 (d, J = 2.56 Hz, 2H). 8.22 (s,

2H), 8.26 (s, 2H), 13.37 (s, 2H), 13.85 (s, 2H). ^{13}C NMR (125 MHz, $CDCl_3$) δ (ppm): 24.38, 24.84, 28.88, 29.26, 29.41, 33.14, 33.28, 34.33, 35.03, 55.85, 72.23, 72.70, 111.56, 117.95, 118.28, 118.46, 121.50, 122.97, 138.67, 138.85, 141.68, 151.29, 154.92, 158.19, 164.89, 165.46, 172.40. HRMS (DART-MS) (m/z): Calculated for [**6-Ligand**+1H]$^+$ ([$C_{66}H_{91}N_4O_{10}$]$^+$) = 1099.6730; found = 1099.6676.

7-Ligand: Was synthesized via the "General Salen Ligand Procedure" using **7-Salen Moiety** and suberoyl chloride. Column conditions: 30% Et_2O in hexanes. The resulting product was a yellow powder (384 mg, 72% yield). ^{1}H NMR (500 MHz, $CDCl_3$) *δ* (ppm): 1.23 (s, 18H), 1.38 (s, 18H), 1.45 (m, 8H), 1.88 (m, 16H), 2.23 (s, 6H), 2.51 (t, J = 7.69 Hz, 4H), 3.31 (m, 4H), 6.77 (d, J = 2.59 Hz, 2H), 6.92 (d, J = 2.81 Hz, 2H), 6.98 (d, J = 2.15 Hz, 2H), 7.16 (d, J = 2.10 Hz, 2H), 8.20 (s, 2H), 8.30 (s, 2H), 13.35 (s, 2H), 13.86 (s, 2H). ^{13}C NMR (125 MHz, $CDCl_3$) *δ* (ppm): 15.85, 24.35, 24.37, 24.85, 28.88, 29.27, 31.53, 33.25, 33.36, 33.92, 34.34, 35.03, 72.52, 72.79, 117.31, 118.29, 121.52, 122.93, 125.18, 125.49, 130.94, 138.68, 140.84, 141.64, 157.09, 158.22, 164.62, 165.46, 172.73. HRMS (DART-MS) (m/z): Calculated for [**7-Ligand**+1H]$^+$ ([$C_{66}H_{91}N_4O_8$]$^+$) = 1067.6831; found = 1067.6841.

8-Ligand: Was synthesized via the "General Salen Ligand Procedure" using **8-Salen Moiety** and suberoyl chloride. Silica plug: 30% Et_2O in hexanes with 1% Et_3N. The resulting product was a yellow powder (562 mg, 86% yield). ^{1}H NMR (500 MHz, $CDCl_3$) *δ* (ppm): 1.24 (s, 18H), 1.38 (s, 18H), 1.45 (m, 8H), 1.77 (m, 20H), 1.88 (m, 4H), 1.94 (m, 4H), 2.07 (s, 6H), 2.15 (s, 12H), 2.51 (t, J = 7.54 Hz, 4H), 3.33 (m, 4H), 6.77 (d, J = 2.77 Hz, 2H), 6.92 (d, J = 2.80 Hz, 2H), 6.97 (d, J = 2.24 Hz, 2H), 7.25 (d, J = 2.37 Hz, 2H), 8.23 (s, 2H), 8.30 (s, 2H), 13.59 (s, 2H), 13.81 (s, 2H). ^{13}C NMR (125 MHz, $CDCl_3$) δ (ppm): 24.44, 24.83, 28.90, 29.27, 29.29, 31.57, 33.22, 33.38, 34.23, 34.33, 35.03, 37.29, 37.35, 40.44, 72.34, 72.58, 117.88, 118.34, 121.53, 122.88, 126.00, 127.03, 136.79, 138.66, 140.19, 141.64, 158.24, 158.33, 164.85, 166.15, 172.68. HRMS (DART-MS) (m/z): Calculated for [**8-Ligand**+1H]$^+$ ([$C_{84}H_{115}N_4O_8$]$^+$) = 1307.8709; found = 1307.8671.

9-Ligand: Was synthesized via the "General Salen Ligand Procedure" using **9-Salen Moiety** and suberoyl chloride. Silica plug: 30% Et_2O in hexanes with 1% Et_3N. The resulting product was a yellow powder (411 mg, 54% yield). ^{1}H NMR (500 MHz, $CDCl_3$) *δ* (ppm): 1.11 (s, 18H), 1.36 (m, 2H), 1.40 (s, 18H), 1.55 (m, 8H), 1.68 (m, 2H), 1.83 (m, 12H), 2.60 (t, J = 7.53 Hz, 4H), 3.18 (m, 4H), 6.67 (d, J = 2.78 Hz, 2H), 6.97 (d, J = 2.80 Hz, 2H), 7.06 (s, 2H), 7.18 (m, 30H) 7.25 (d, J = 2.47 Hz, 2H), 7.95 (s, 2H), 8.23 (s, 2H), 13.21 (s, 2H), 13.89 (s, 2H). ^{13}C NMR (125 MHz, $CDCl_3$) *δ* (ppm): 24.21, 24.26, 24.79, 28.87, 29.17, 29.72, 31.24, 32.89, 33.03, 33.99, 34.26, 34.92, 63.32, 71.87, 72.74, 117.87, 118.12, 121.42, 122.89, 125.46, 127.09, 131.02, 131.67, 133.81, 138.54, 139.69, 141.48, 145.63,

157.62, 158.17, 164.50, 165.40, 172.57. HRMS (DART-MS) (m/z): Calculated for [**9-Ligand**+1H]$^+$ ([$C_{102}H_{115}N_4O_8$]$^+$) = 1523.8709; found = 1523.8715.

10-Ligand: Was synthesized via the "General Salen Ligand Procedure" using **1-Salen Moiety** and **C4-Diacid Chloride**. Silica plug: 20% Et_2O in hexanes with 3% Et_3N. The resulting product was a yellow powder (543 mg, 90% yield). ^{1}H NMR (500 MHz, $CDCl_3$) δ (ppm): 1.24 (s, 18H), 1.28 (d, J = 2.09 Hz, 12H), 1.34 (m, 4H), 1.39 (s, 18H), 1.41 (s, 18H), 1.46 (m, 4H), 1.66 (m, 8H), 1.94 (m, 8H), 3.32 (m, 4H), 6.72 (d, J = 2.81 Hz, 2H), 6.87 (d, J = 2.74 Hz, 2H), 6.98 (d, J = 2.42 Hz, 2H), 7.32 (d, J = 2.38 Hz, 2H), 8.22 (s, 2H), 8.30 (s, 2H), 13.62 (s, 2H), 13.86 (s, 2H). ^{13}C NMR (125 MHz, $CDCl_3$) δ (ppm): 24.41, 25.23, 25.37, 25.63, 29.27, 29.58, 31.56, 33.24, 33.27, 34.17, 34.98, 35.08, 40.64, 42.60, 72.32, 72.57, 117.87, 118.33, 121.44, 122.77, 126.07, 127.03, 136.47, 138.60, 140.09, 141.91, 158.06, 158.12, 164.86, 166.02, 177.01. HRMS (DART-MS) (m/z): Calculated for [**10-Ligand**+1H]$^+$ ([$C_{76}H_{111}N_4O_8$]+) = 1207.8396; found = 1207.8435 (Mass Error: 3.23 ppm).

11-Ligand: Was synthesized via the "General Salen Ligand Procedure" using **1-Salen Moiety** and **C3-Diacid Chloride**. Silica plug: 20% Et_2O in hexanes with 3% Et_3N. The resulting product was a yellow powder (537 mg, 90% yield). ^{1}H NMR (500 MHz, $CDCl_3$) δ (ppm): 1.24 (s, 18H), 1.29 (s, 12H), 1.37 (m, 18H), 1.41 (s, 18H), 1.47 (m, 6H), 1.71 (m, 8H), 1.93 (m, 8H), 3.29 (m, 4H), 6.69 (d, J = 2.76 Hz, 2H), 6.86 (d, J = 2.78 Hz, 2H), 6.98 (d, J = 2.41 Hz, 2H), 7.32 (d, J = 2.42 Hz, 2H), 8.13 (s, 2H), 8.29 (s, 2H), 13.60 (s, 2H), 13.88 (s, 2H). ^{13}C NMR (125 MHz, $CDCl_3$) δ (ppm): 24.39, 25.27, 29.27, 29.60, 31.56, 33.19, 34.17, 34.98, 35.09, 41.14, 42.66, 72.18, 72.54, 117.88, 118.32, 121.49, 122.84, 126.06, 127.02, 136.50, 138.66, 140.07, 141.87, 158.07, 158.16, 164.81, 166.00, 176.96. HRMS (DART-MS) (m/z): Calculated for [**11-Ligand**+1H]$^+$ ([$C_{75}H_{109}N_4O_8$]$^+$) = 1193.8240; found = 1193.8274.

12-Ligand: Was synthesized via the "General Salen Ligand Procedure" using **1-Salen Moiety** and **C5-Diacid Chloride**. Silica plug: 20% Et_2O in hexanes with 3% Et_3N. The resulting product was a yellow powder (397 mg, 65% yield). ^{1}H NMR (500 MHz, $CDCl_3$) δ (ppm): 1.24 (s, 18H), 1.27 (s 12H), 1.35 (m, 5H) 1.39 (s, 18H), 1.41 (s, 18H), 1.46 (m, 5H), 1.73 (m, 8H), 1.94 (m, 8H), 3.32 (m, 4H), 6.72 (d, J = 2.77 Hz, 2H), 6.88 (d, J = 2.84 Hz, 2H), 6.98 (d, J = 2.51 Hz, 2H), 7.32 (d, J = 2.44 Hz, 2H), 8.24 (s, 2H), 8.30 (s, 2H), 13.62 (s, 2H), 13.84 (s, 2H). ^{13}C NMR (125 MHz, $CDCl_3$) δ (ppm): 24.41, 25.03, 25.25, 25.28, 29.27, 29.58, 30.74, 31.56, 33.23, 33.28, 34.17, 34.98, 35.08, 40.71, 42.60, 72.33, 72.56, 117.87, 118.33, 121.42, 122.79, 126.08, 127.04, 136.48, 138.58, 140.09, 141.94, 158.06, 158.10, 164.88, 166.03, 177.08. HRMS (DART-MS) (m/z): Calculated for [**12-Ligand**+1H]+ ([$C_{77}H_{113}N_4O_8$]$^+$) = 1221.8553; found = 1221.8602.

13-Ligand: Was synthesized via the "General Salen Ligand Procedure" using **8-Salen Moiety** and **C3-Diacid Chloride**. Silica plug: 20% Et_2O in hexanes with 3% Et_3N. The resulting product was a yellow powder (580 mg, 86% yield). ^{1}H NMR (500 MHz, $CDCl_3$) δ (ppm): 1.23 (s, 18H), 1.28 (s, 12H), 1.36 (s, 18H), 1.43 (m, 6H), 1.67 (m, 8H), 1.78 (m, 12H) 1.91 (m, 8H), 2.06 (m, 6H), 2.14 (s, 12H), 3.29 (m, 4H), 6.68 (d, J = 2.83 Hz, 2H), 6.85 (d, J = 2.73 Hz, 2H), 6.96 (d, J = 2.22 Hz, 2H), 7.25 (d, J = 2.51 Hz, 2H), 8.12 (s, 2H), 8.28 (s, 2H), 13.59 (s, 2H), 13.89 (s, 2H). ^{13}C NMR (125 MHz, $CDCl_3$) *δ* (ppm): 24.39, 24.41, 25.23, 25.27, 29.27, 31.57, 33.10, 33.27, 34.22, 34.98, 37.29, 37.35, 40.43, 41.10, 42.65, 72.12, 72.64, 117.86, 118.32, 121.45, 122.82, 125.97, 126.99, 136.79, 138.65, 140.15, 141.86, 158.19, 158.33, 164.79, 166.13, 176.97. HRMS (DART-MS) (m/z): Calculated for [**13-Ligand**+1H]$^+$ ($[C_{87}H_{120}N_4O_8]^+$) = 1349.9184; found = 1349.9216.

7.6 Catalysts

General Metalation Procedure:

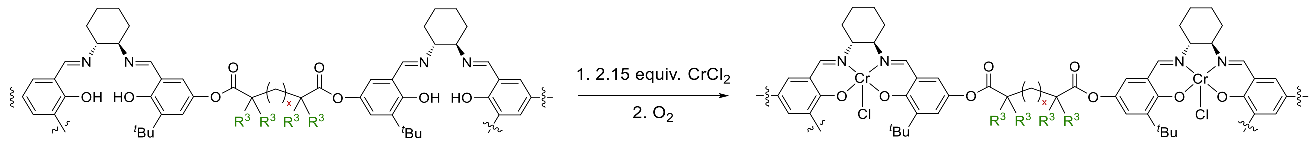

#-Ligand (1 equiv., 0.2 mmol) was added to a flame dried Schlenk flask under an inert atmosphere before being dissolved in minimal THF. While working in a N_2 filled glovebox, anhydrous chromium(II) chloride (2.15 equiv., 0.43 mmol, 53 mg) was added to second Schlenk flask. The flask was sealed, removed from the glovebox, and placed under a N_2 atmosphere using a standard Schlenk line. A grey, heterogeneous slurry was made from adding THF (4 mL) to this flask. The dissolved ligand was then added by cannula to the suspension of chromium(II) chloride in THF. The reaction was then sealed and stirred at 40 °C for three hours to yield a homogeneous dark red-brown solution. After coming to room temperature, the reaction vessel was fitted with a drying tube and stirred overnight in the presence of O_2 (exposed to air). The resulting solution was diluted to twice its original volume with diethyl ether and washed with saturated NH_4Cl (3×) and brine (3×). The organic layer was dried over Na_2SO_4, filtered, and dried under vacuum. The crude solid was purified by sonicating in pentanes and filtered over a fine glass frit to yield a dark red-brown solid. Due to their paramagnetic nature, these complexes were not characterized by NMR spectrometry.

1: Was synthesized via the "General Metalation Procedure" using **1-Ligand**. The resulting product was a dark red-brown powder (224 mg, 85% yield). HRMS (DART-MS) (m/z): Calculated for [**1**–2Cl]$^{2+}$ ([$C_{72}H_{98}N_4O_8Cr_2$]$^{2+}$) = 625.30975; found = 625.30825.

2: Was synthesized via the "General Metalation Procedure" using **2-Ligand**. The resulting product was a dark red-brown powder (178 mg, 72% yield). HRMS (DART-MS) (m/z): Calculated for [**2**–1Cl]$^{+}$ ([$C_{66}H_{86}N_4O_8ClCr_2$]$^{+}$) = 1201.4944; found = 1201.4926.

3: Was synthesized via the "General Metalation Procedure" using **3-Ligand**. The resulting product was a dark red-brown powder (216 mg, 64% yield). HRMS (DART-MS) (m/z): Calculated for [**3**–1Cl]$^{+}$ ([$C_{102}H_{110}N_4O_8ClCr_2$]$^{+}$) = 1657.6822; found = 1657.6806.

4: Was synthesized via the "General Metalation Procedure" using **4-Ligand**. The resulting product was a dark red-brown powder (189 mg, 74% yield). HRMS (DART-MS) (m/z): Calculated for [**4**–1Cl]$^+$ ([$C_{64}H_{80}N_4O_8Cl_3Cr_2$]$^+$) = 1241.3852; found = 1241.3839.

5: Was synthesized via the "General Metalation Procedure" using **5-Ligand**. The resulting product was a dark red-brown powder (194 mg, 78% yield). HRMS (DART-MS) (m/z): Calculated for [**5**–2Cl]$^{2+}$ ([$C_{64}H_{80}N_4O_8F_2Cr_2$]$^{2+}$) = 587.23773; found = 587.23610.

6: Was synthesized via the "General Metalation Procedure" using **6-Ligand**. The resulting product was a dark red-brown powder (185 mg, 73% yield). HRMS (DART-MS) (m/z): Calculated for [**6**–1Cl]$^+$ ([$C_{66}H_{86}N_4O_{10}Cr_2Cl$]$^+$) = 1233.4843; found = 1233.4835.

7: Was synthesized via the "General Metalation Procedure" using **7-Ligand**. The resulting product was a dark red-brown powder (173 mg, 70% yield). HRMS (DART-MS) (m/z): Calculated for [**7**–2Cl]$^{2+}$ ([$C_{66}H_{86}N_4O_8Cr_2$]$^{2+}$) = 583.2623; found = 583.2613.

8: Was synthesized via the "General Metalation Procedure" using 8-Ligand. The resulting product was a dark red-brown powder (254 mg, 86% yield). HRMS (DART-MS) (m/z): Calculated for [**8**–2Cl]$^{2+}$ ([$C_{84}H_{110}N_4O_8Cr_2$]$^{2+}$) = 703.3567; found = 703.3556.

9: Was synthesized via the "General Metalation Procedure" using **9-Ligand**. The resulting product was a dark red-brown powder (301 mg, 89% yield). HRMS (DART-MS) (m/z): Calculated for [**9**–2Cl]$^{2+}$ ([$C_{102}H_{110}N_4O_8Cr_2$]$^{2+}$) = 811.3567; found = 811.3550.

10: Was synthesized via the "General Metalation Procedure" using **10-Ligand**. The resulting product was a dark red-brown powder (248 mg, 90% yield). HRMS (DART-MS) (m/z): Calculated for [**10**–2Cl]$^{2+}$ ([$C_{76}H_{106}N_4O_8Cr_2$]$^{2+}$) = 653.3411; found = 653.3412.

11: Was synthesized via the "General Metalation Procedure" using **11-Ligand**. The resulting product was a dark red-brown powder (188 mg, 69% yield). HRMS (DART-MS) (m/z): Calculated for [**11**–2Cl]$^{2+}$ ([$C_{75}H_{104}N_4O_8Cr_2$]$^{2+}$) = 646.3332; found = 646.3337.

12: Was synthesized via the "General Metalation Procedure" using **12-Ligand**. The resulting product was a dark red-brown powder (156 mg, 56% yield). HRMS (DART-MS) (m/z): Calculated for [**12**–2Cl]$^{2+}$ ([$C_{77}H_{108}N_4O_8Cr_2$]$^{2+}$) = 660.3489; found = 660.3489.

13: Was synthesized via the "General Metalation Procedure" using **13-Ligand**. The resulting product was a dark red-brown powder (246 mg, 81% yield). HRMS (DART-MS) (m/z): Calculated for [**13**–2Cl]$^{2+}$ ([$C_{87}H_{116}N_4O_8Cr_2$]$^{2+}$) = 724.3802; found = 724.3811.

8. ^{1}H and ^{13}C NMR Spectra

2-Salen Moiety, ^{1}H NMR spectrum (500 MHz, $CDCl_3$)

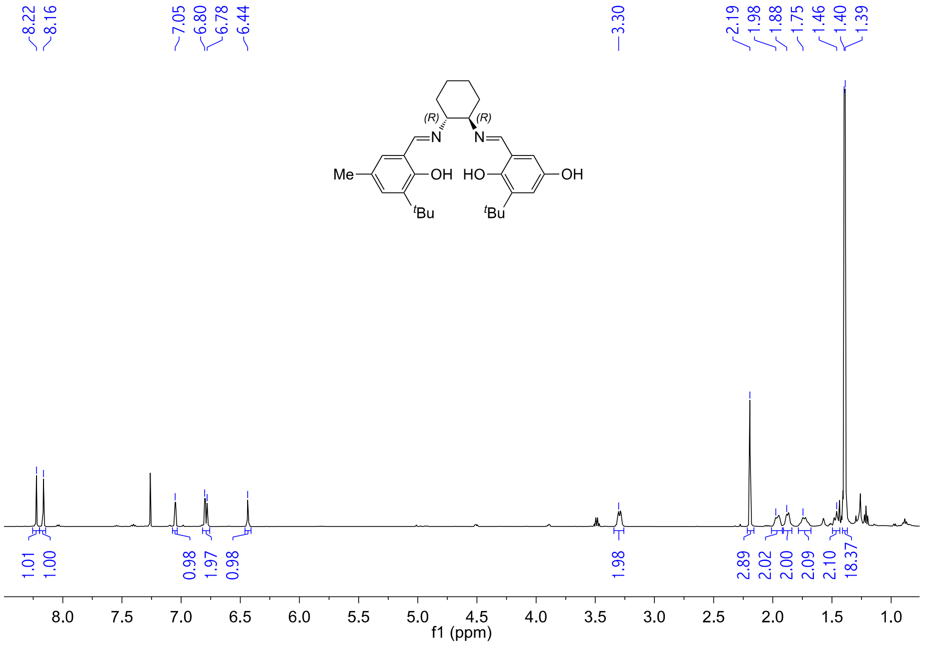

2-Salen Moiety, ^{13}C NMR spectrum (125 MHz, $CDCl_3$)

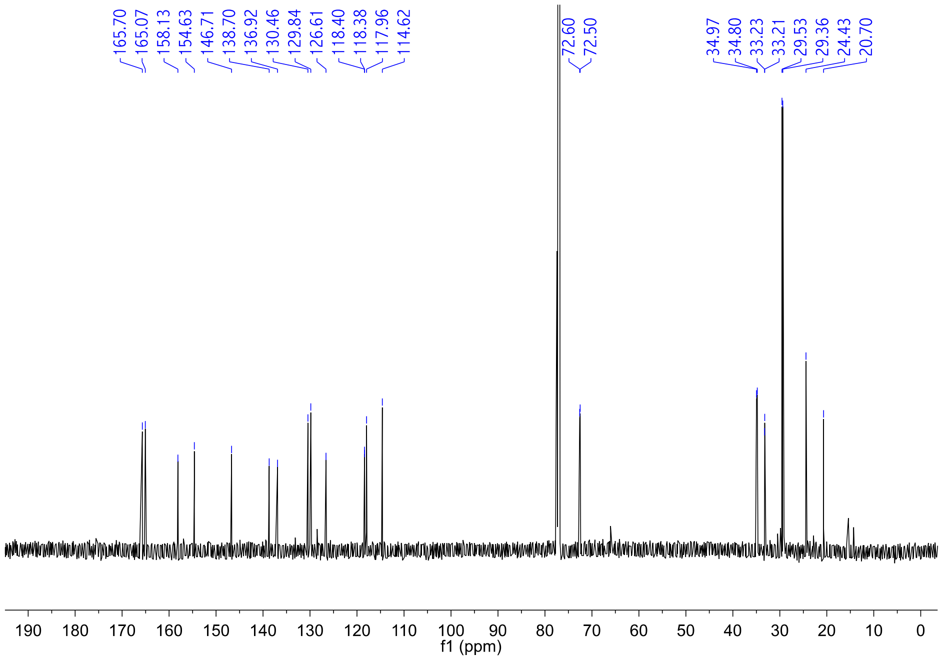

3-Salen Moiety, ^{1}H NMR spectrum (500 MHz, $CDCl_3$)

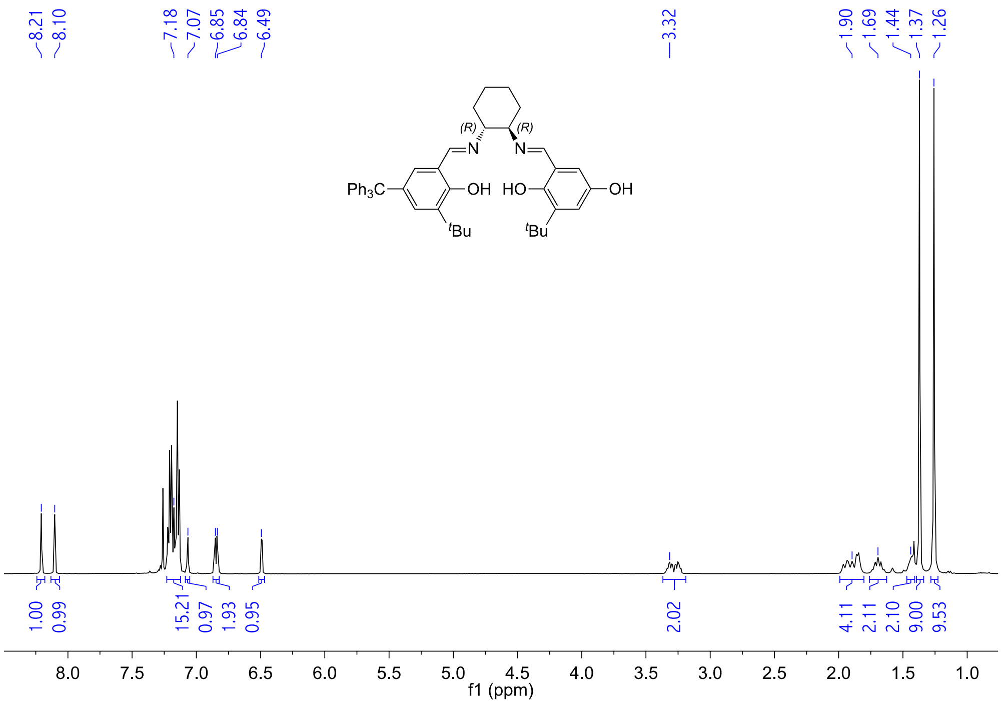

3-Salen Moiety, ^{13}C NMR spectrum (125 MHz, $CDCl_3$)

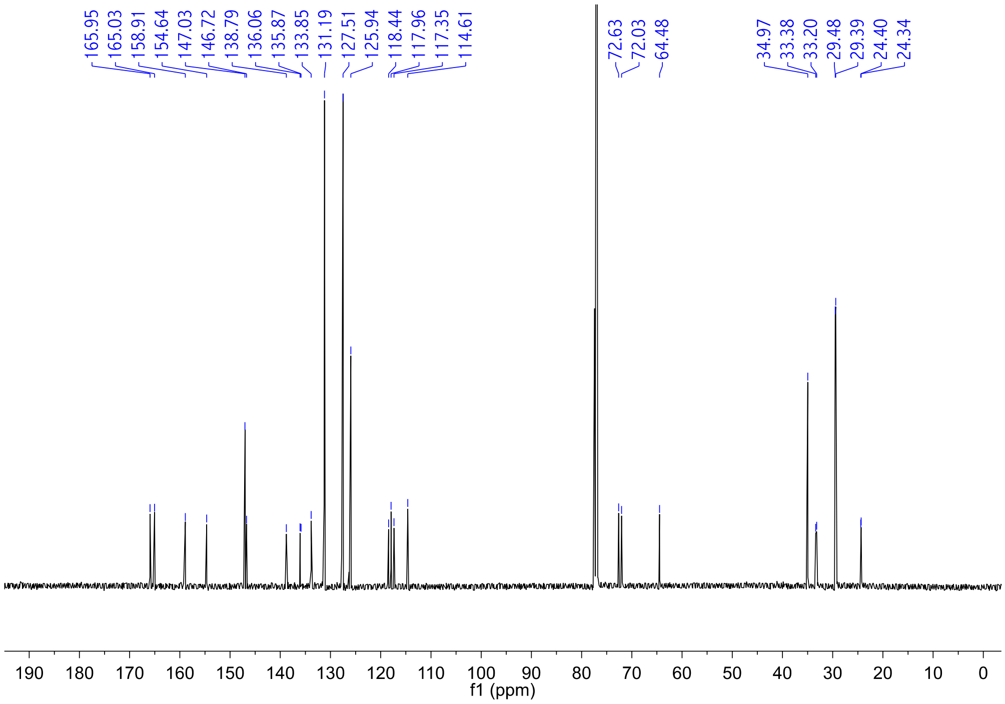

4-Salen Moiety, ^{1}H NMR spectrum (500 MHz, $CDCl_3$)

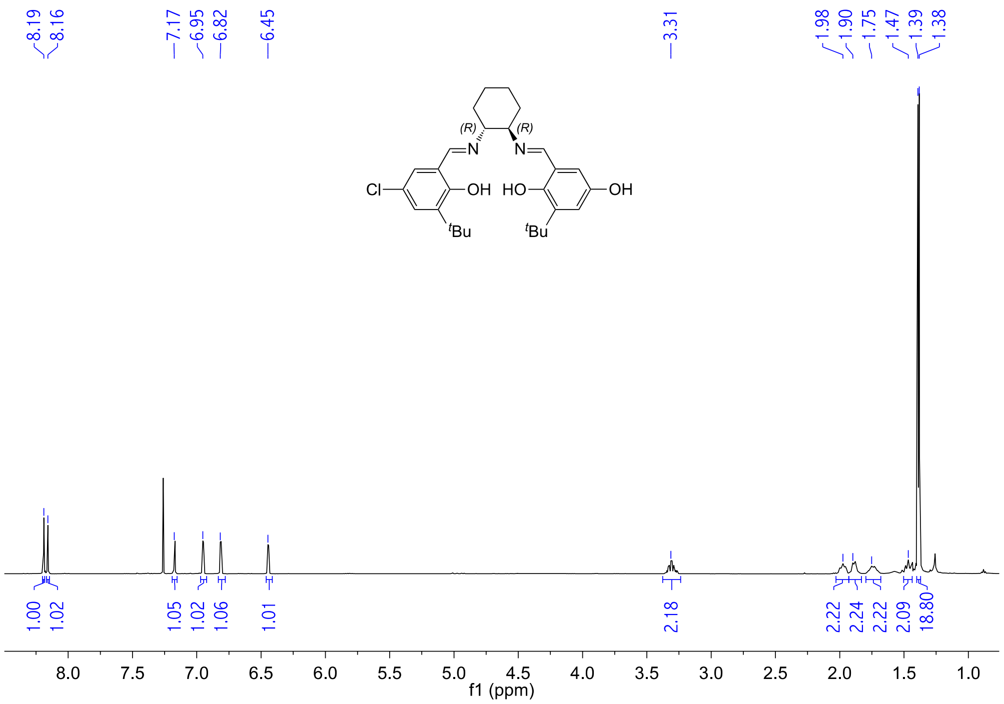

4-Salen Moiety, ^{13}C NMR spectrum (125 MHz, $CDCl_3$)

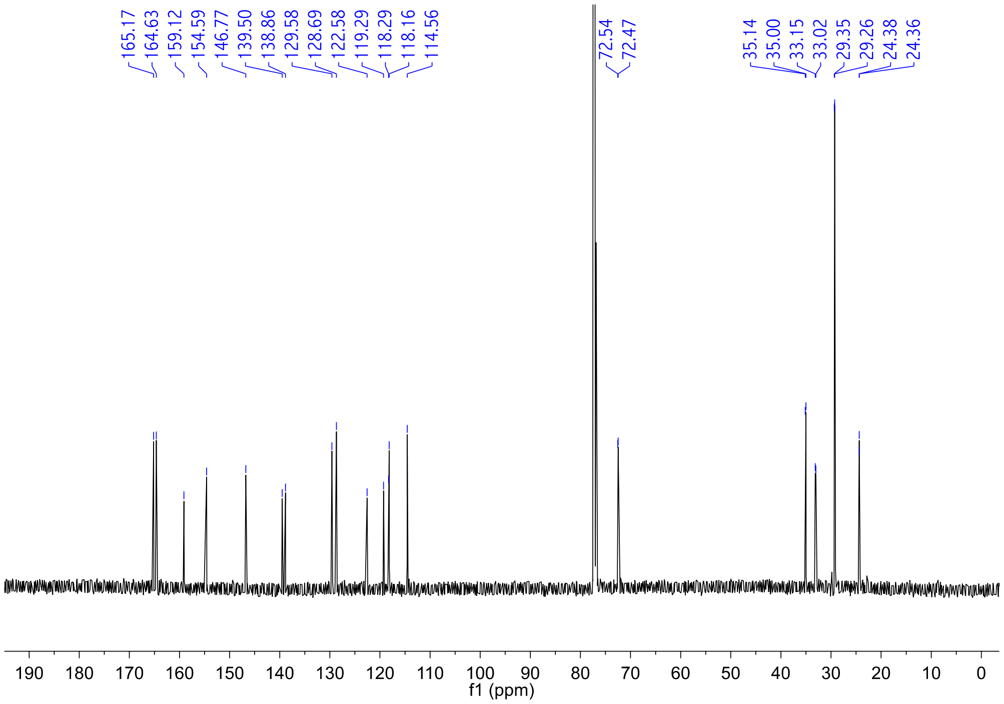

5-Salen Moiety, ^{1}H NMR spectrum (500 MHz, $CDCl_3$)

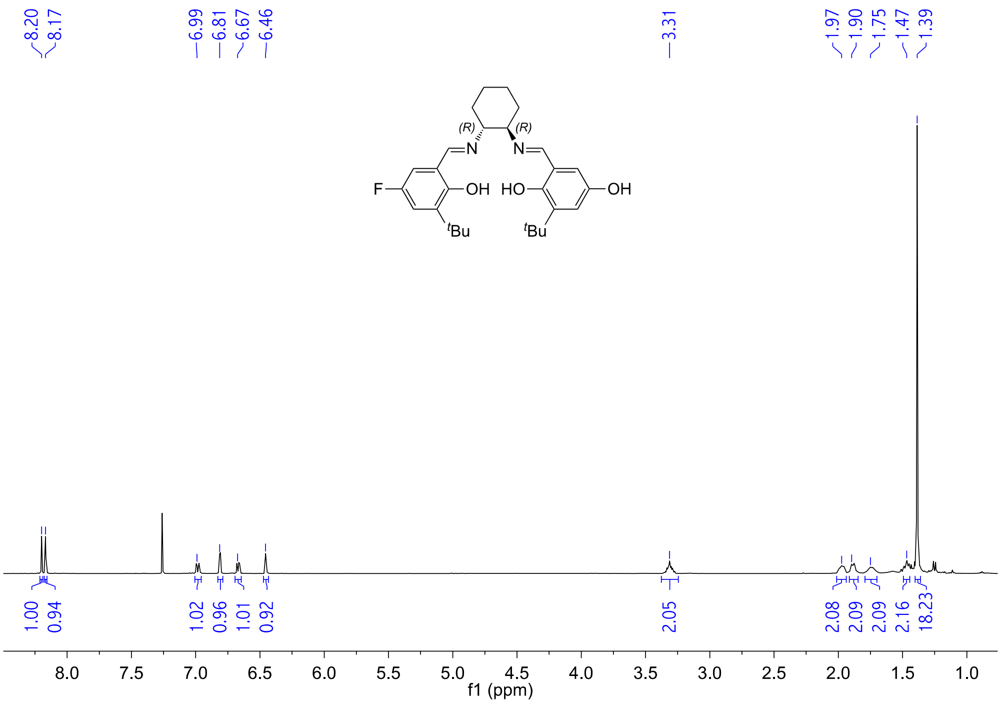

5-Salen Moiety, ^{13}C NMR spectrum (125 MHz, $CDCl_3$)

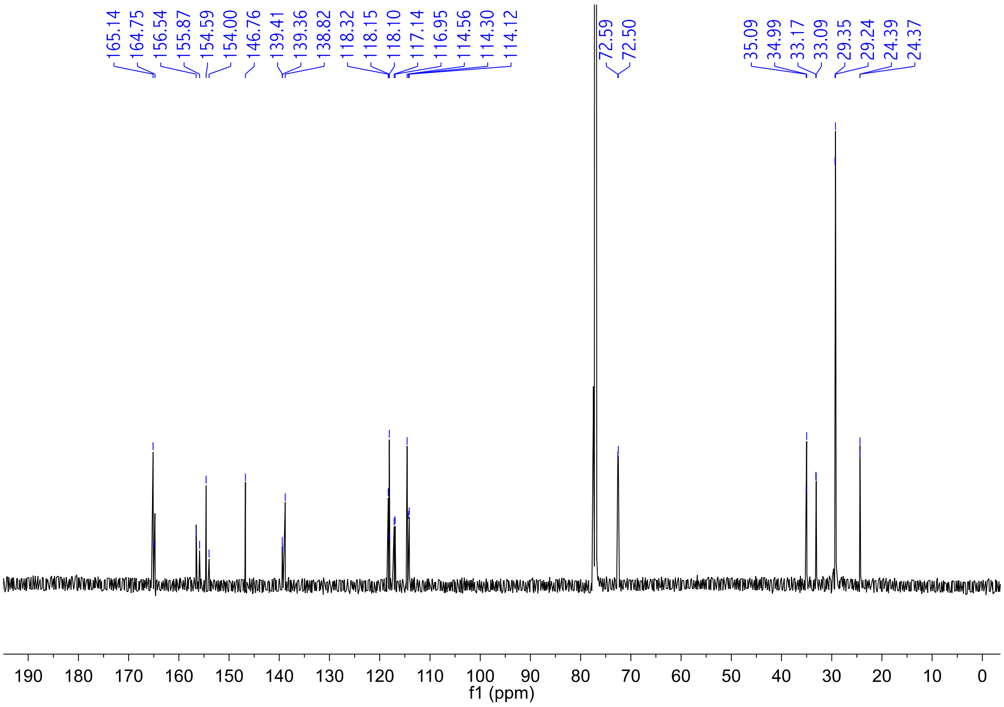

6-Salen Moiety, ^{1}H NMR spectrum (500 MHz, $CDCl_3$)

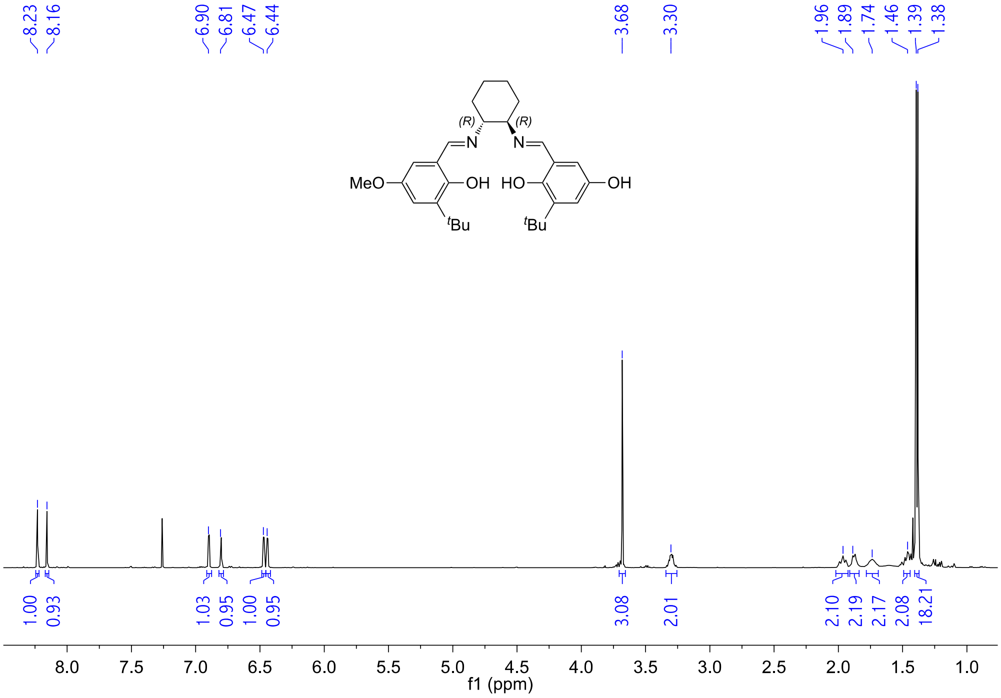

6-Salen Moiety, ^{13}C NMR spectrum (125 MHz, $CDCl_3$)

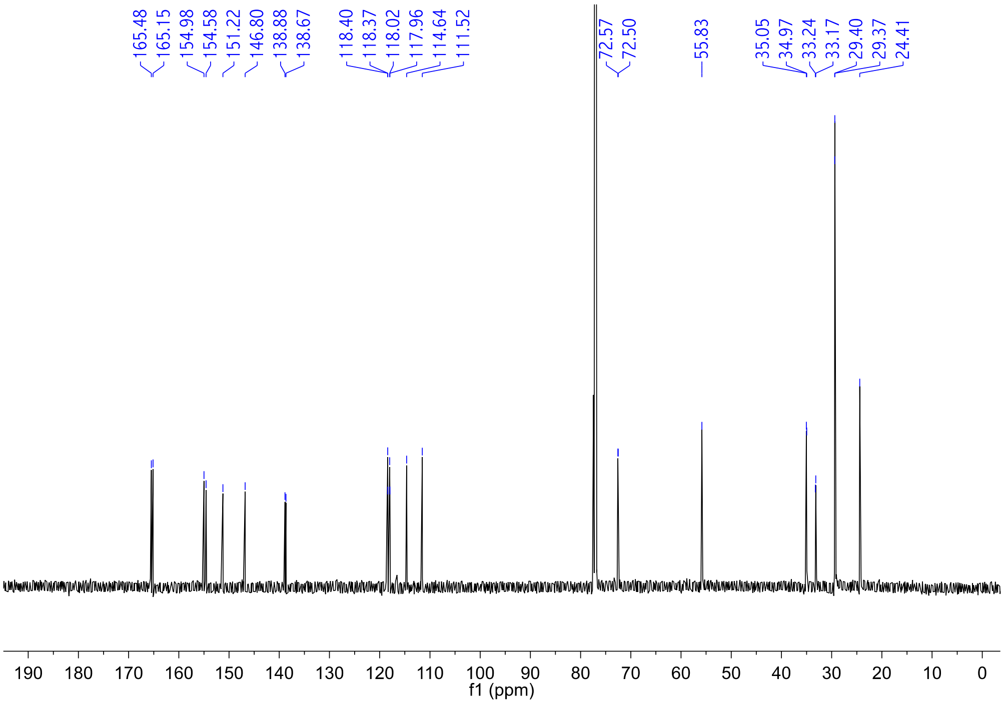

7-Salen Moiety, ^{1}H NMR spectrum (500 MHz, $CDCl_3$)

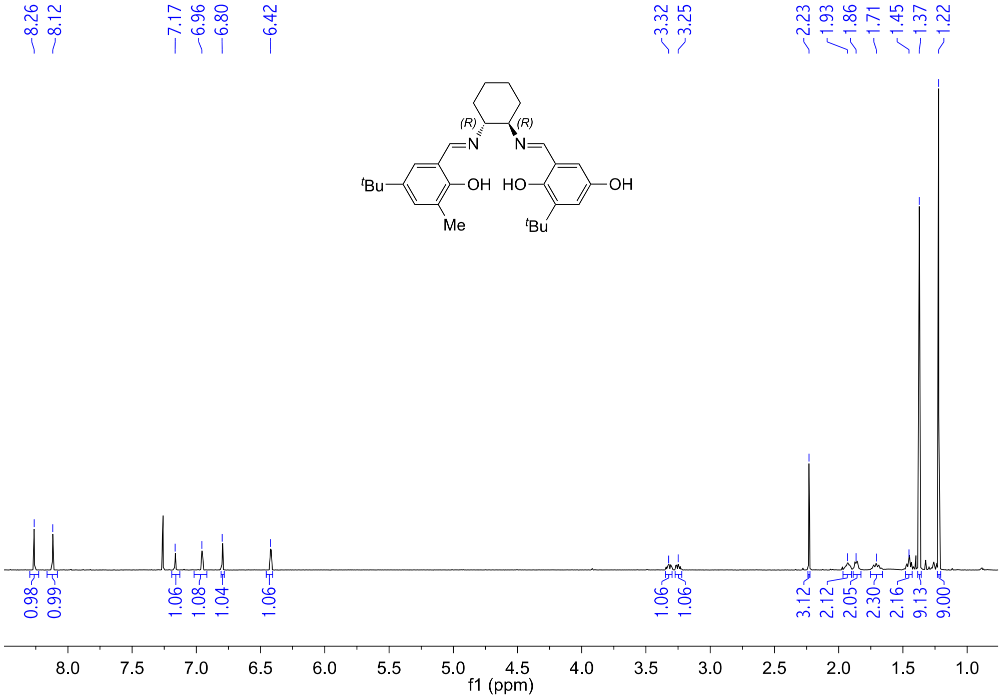

7-Salen Moiety, ^{13}C NMR spectrum (125 MHz, $CDCl_3$)

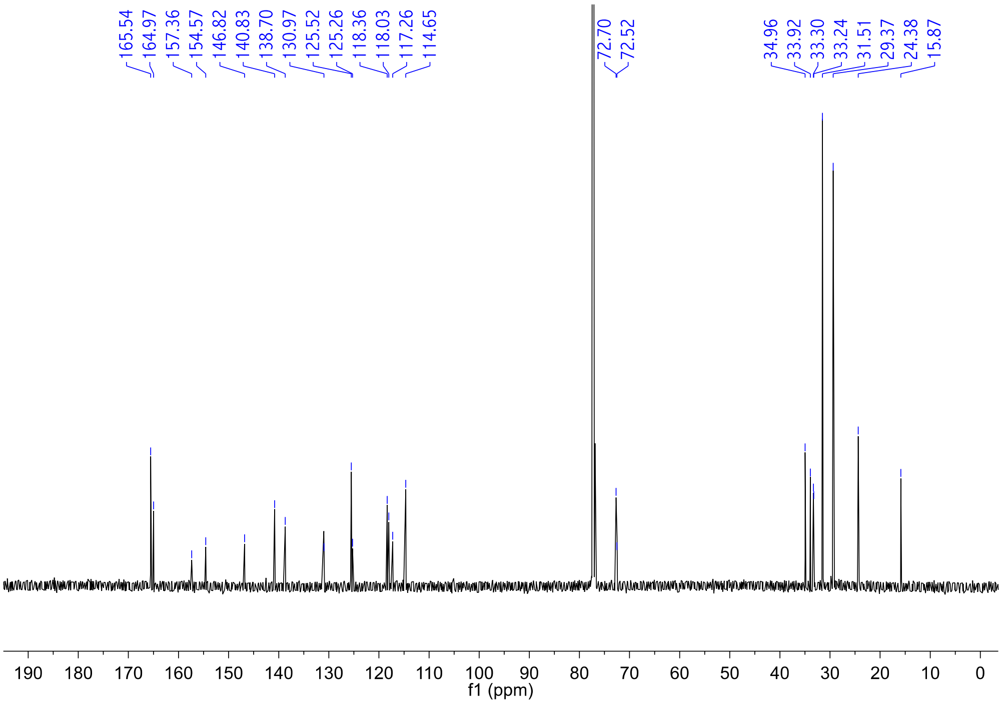

8-Salen Moiety, ^{1}H NMR spectrum (500 MHz, $CDCl_3$)

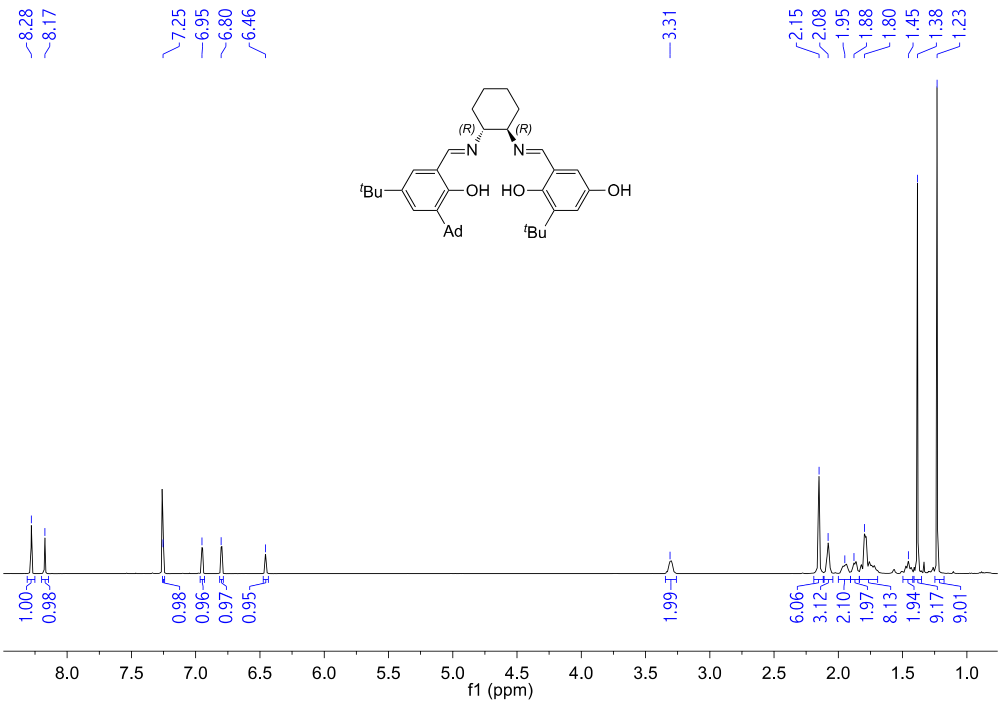

8-Salen Moiety, ^{13}C NMR spectrum (125 MHz, $CDCl_3$)

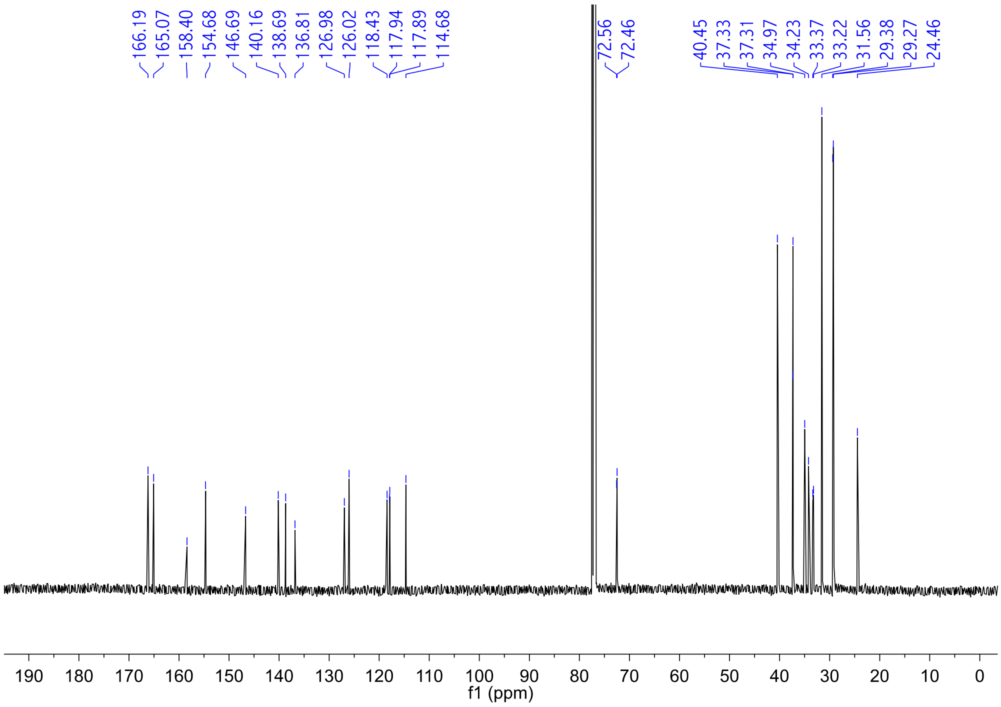

9-Salen Moiety, ^{1}H NMR spectrum (500 MHz, $CDCl_3$)

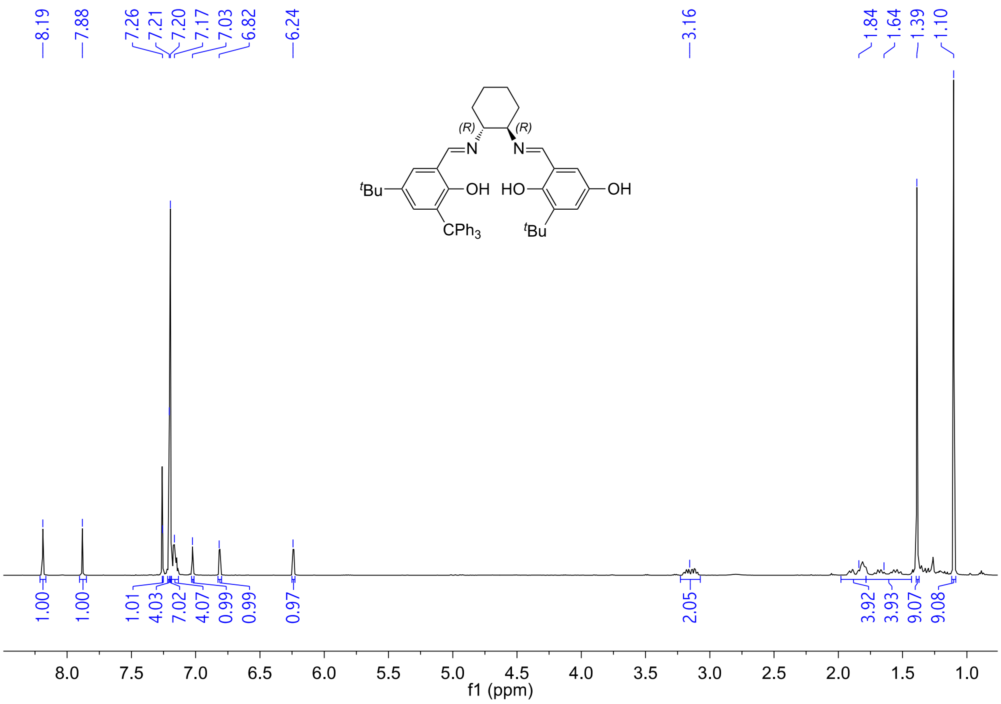

9-Salen Moiety, ^{13}C NMR spectrum (125 MHz, $CDCl_3$)

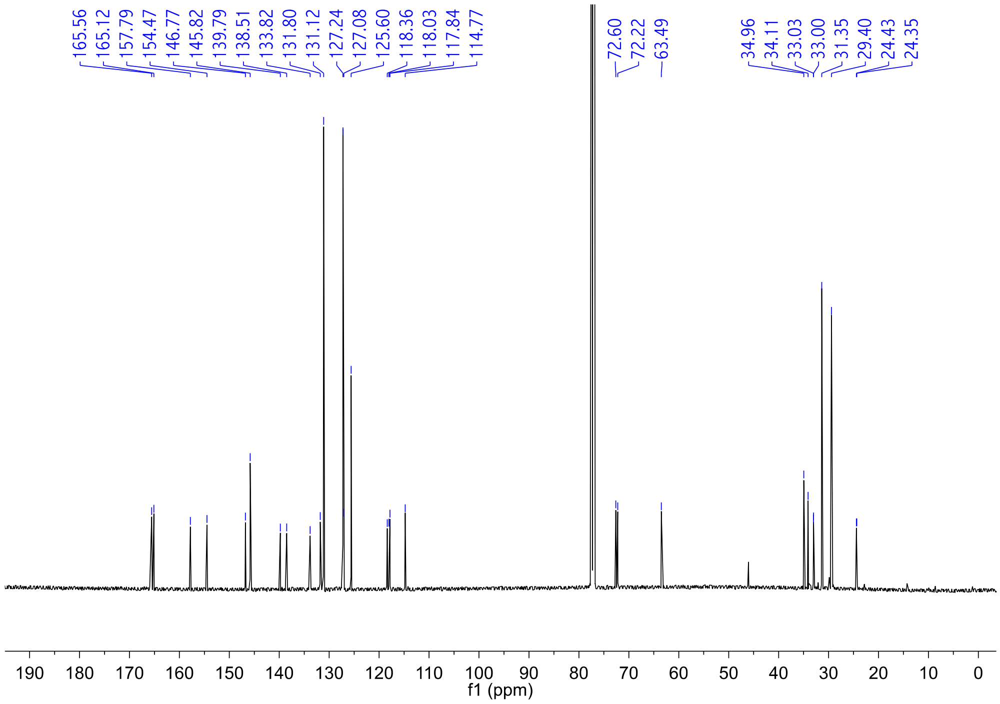

2-Ligand, ^{1}H NMR spectrum (500 MHz, $CDCl_3$)

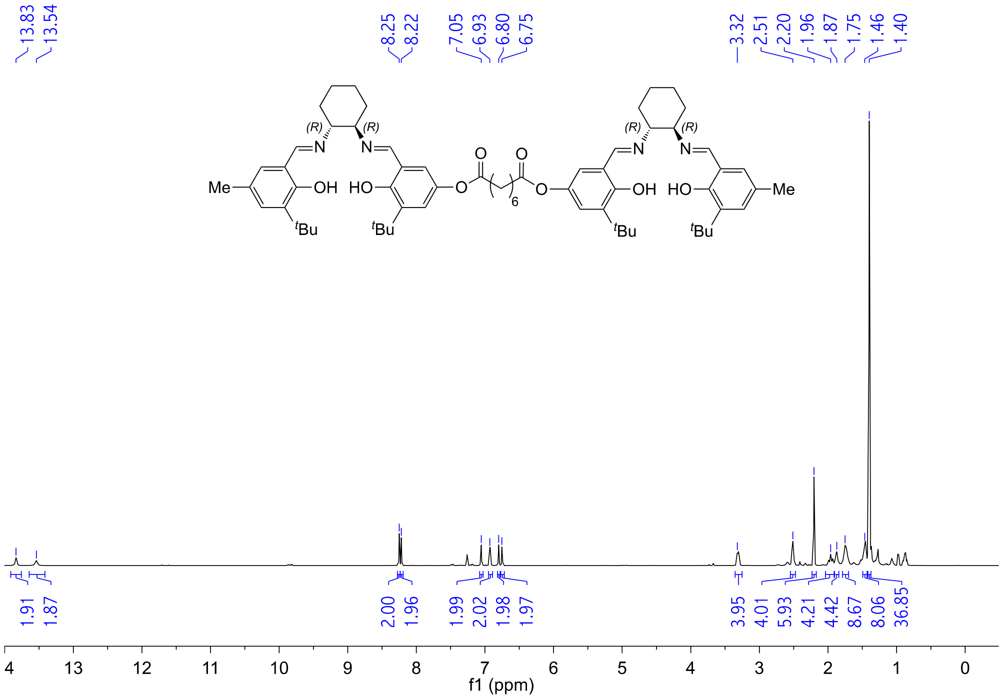

2-Ligand, ^{13}C NMR spectrum (125 MHz, $CDCl_3$)

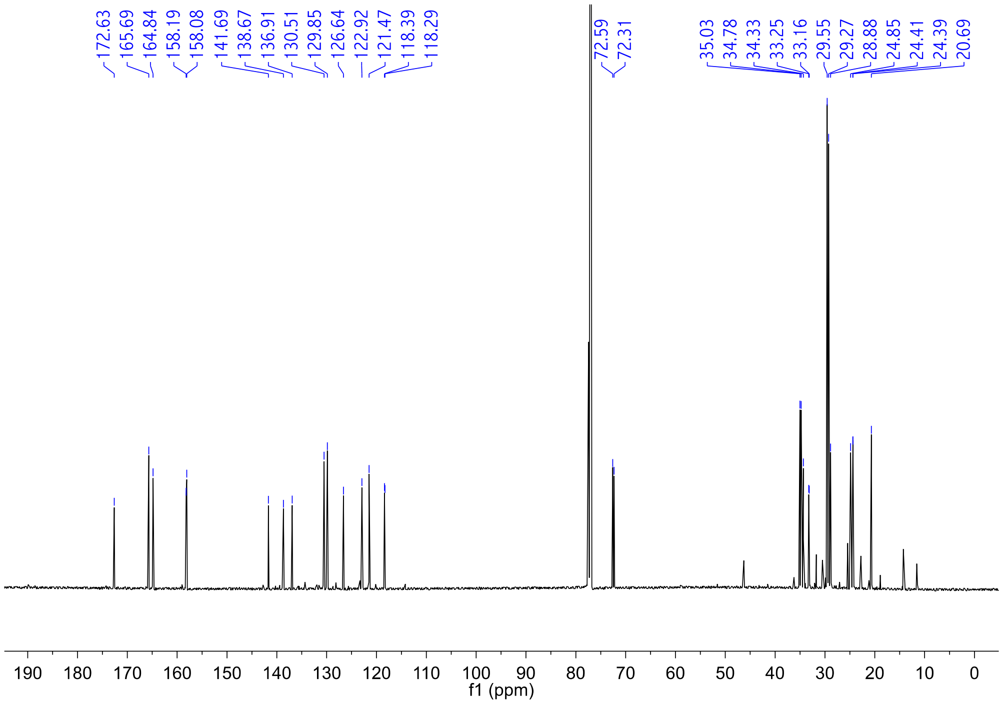

3-Ligand, ^{1}H NMR spectrum (500 MHz, $CDCl_3$)

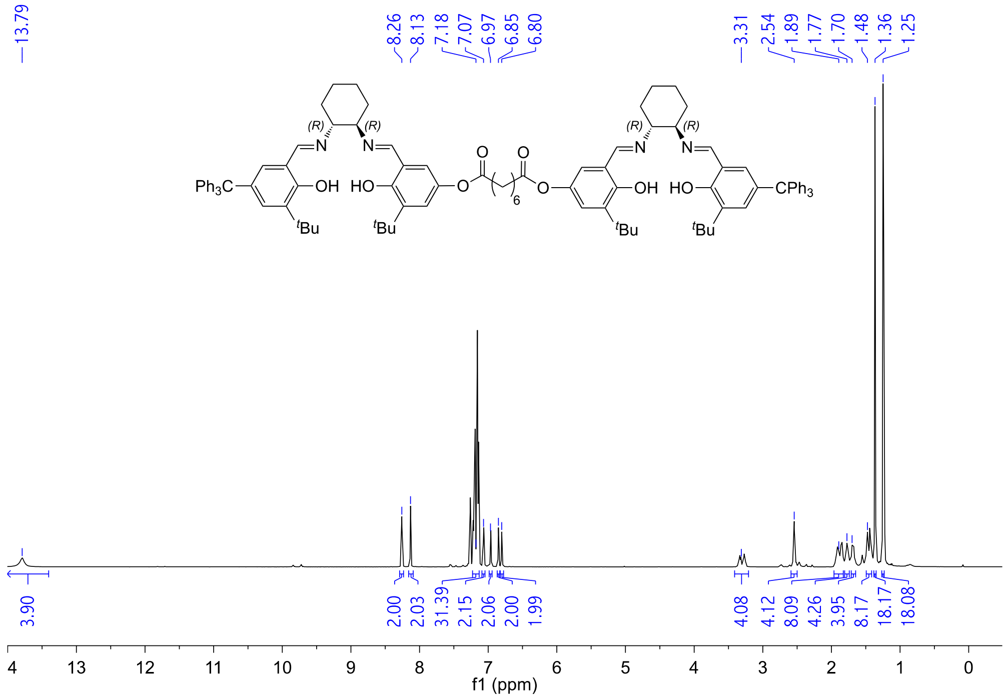

3-Ligand, ^{13}C NMR spectrum (125 MHz, $CDCl_3$)

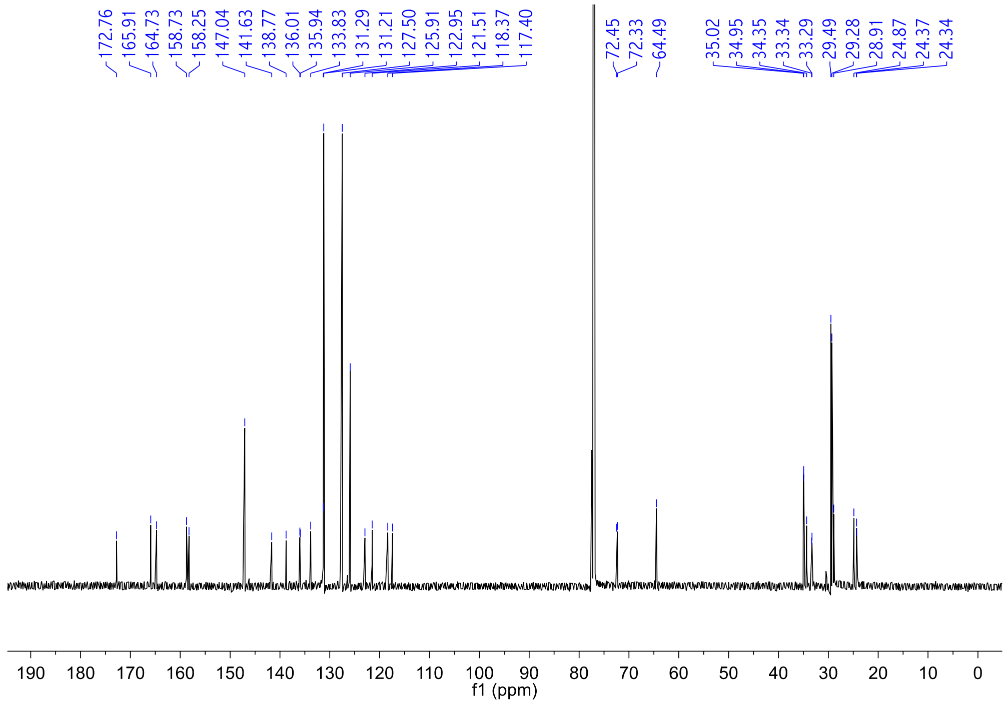

4-Ligand, ^{1}H NMR spectrum (500 MHz, $CDCl_3$)

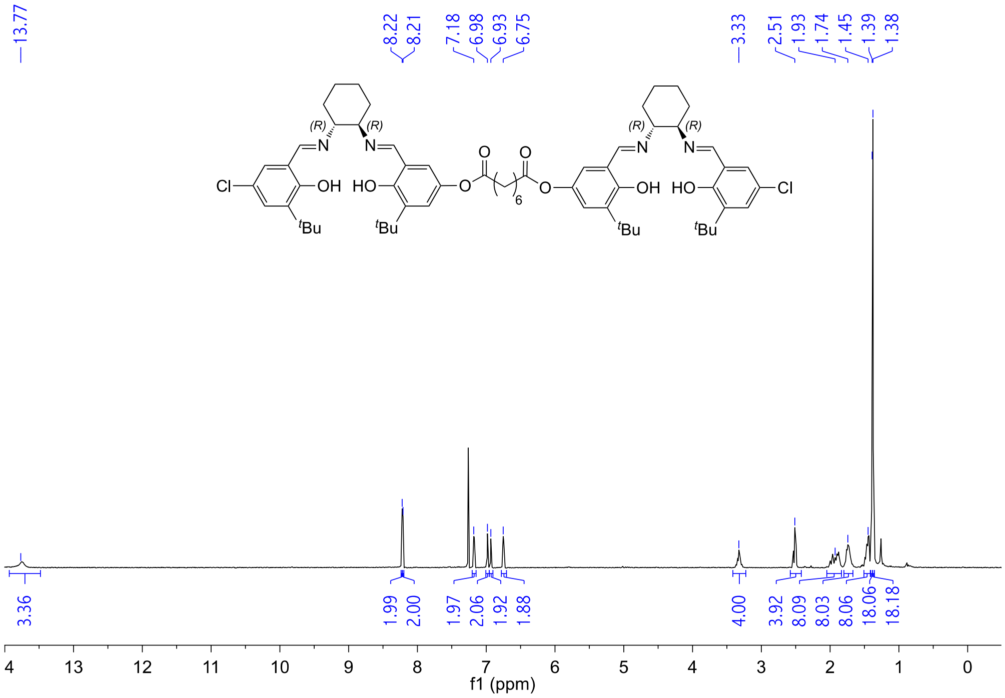

4-Ligand, ^{13}C NMR spectrum (125 MHz, $CDCl_3$)

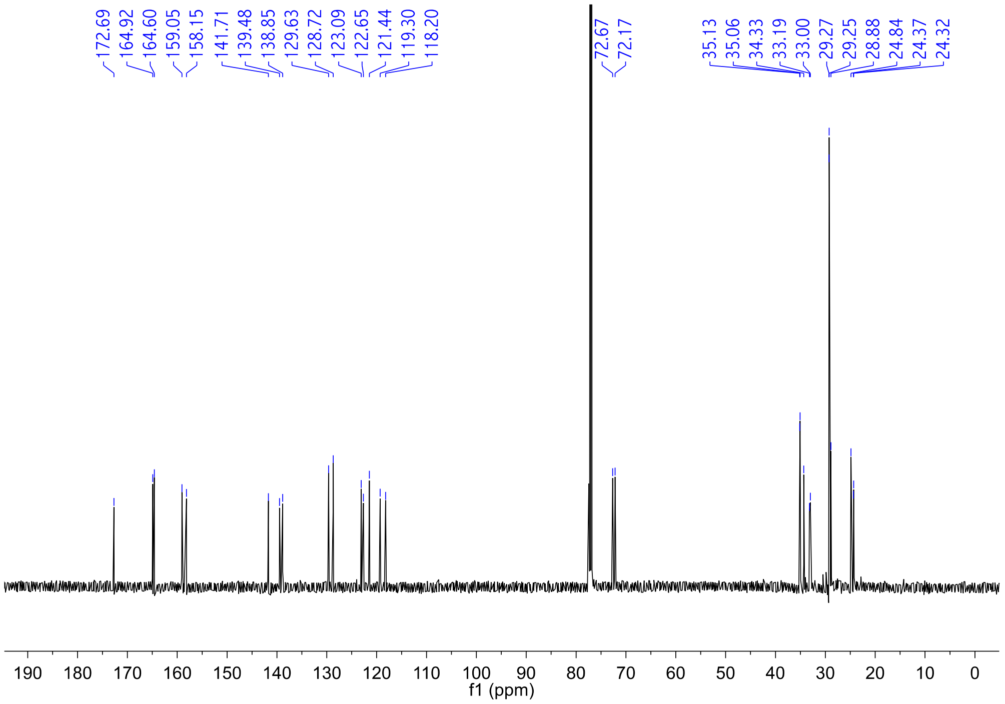

5-Ligand, ^{1}H NMR spectrum (500 MHz, CDCl$_3$)

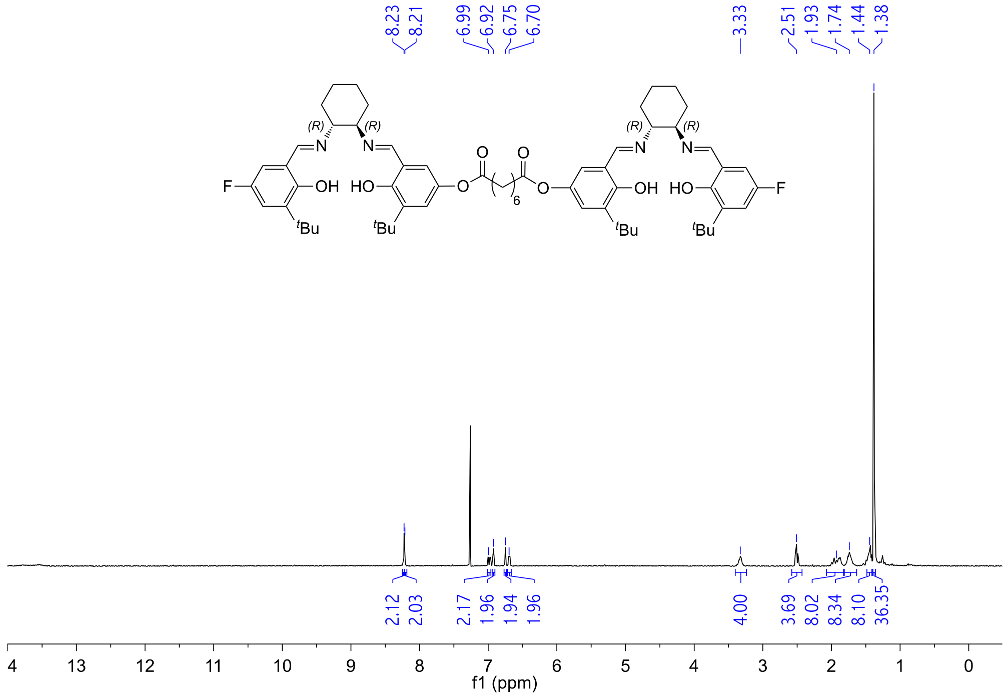

5-Ligand, ^{13}C NMR spectrum (125 MHz, CDCl$_3$)

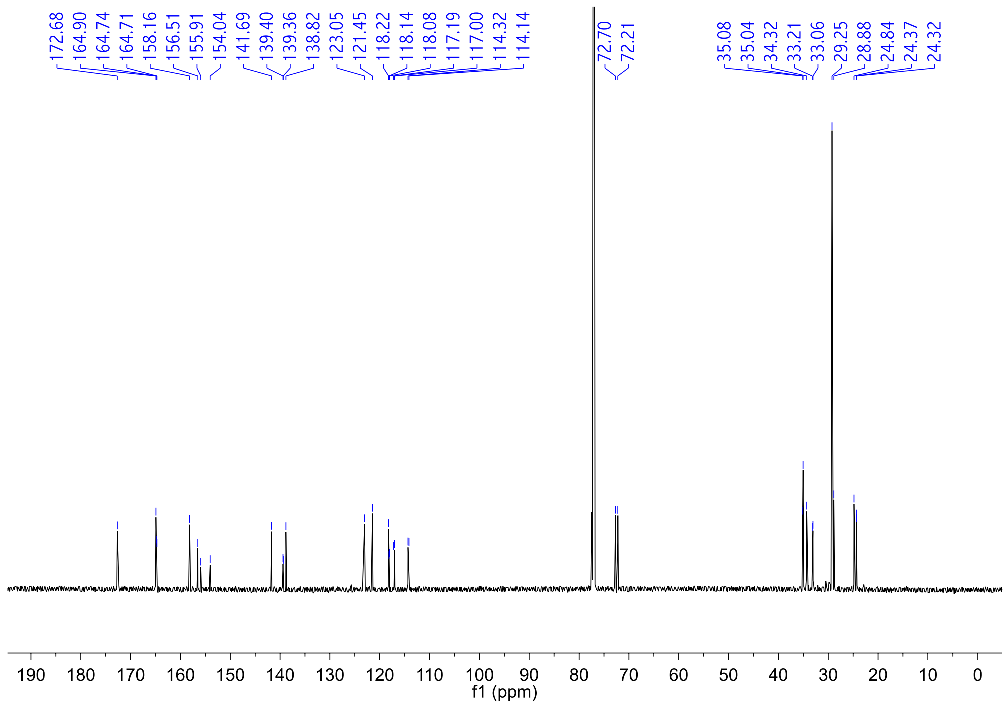

6-Ligand, ^{1}H NMR spectrum (500 MHz, $CDCl_3$)

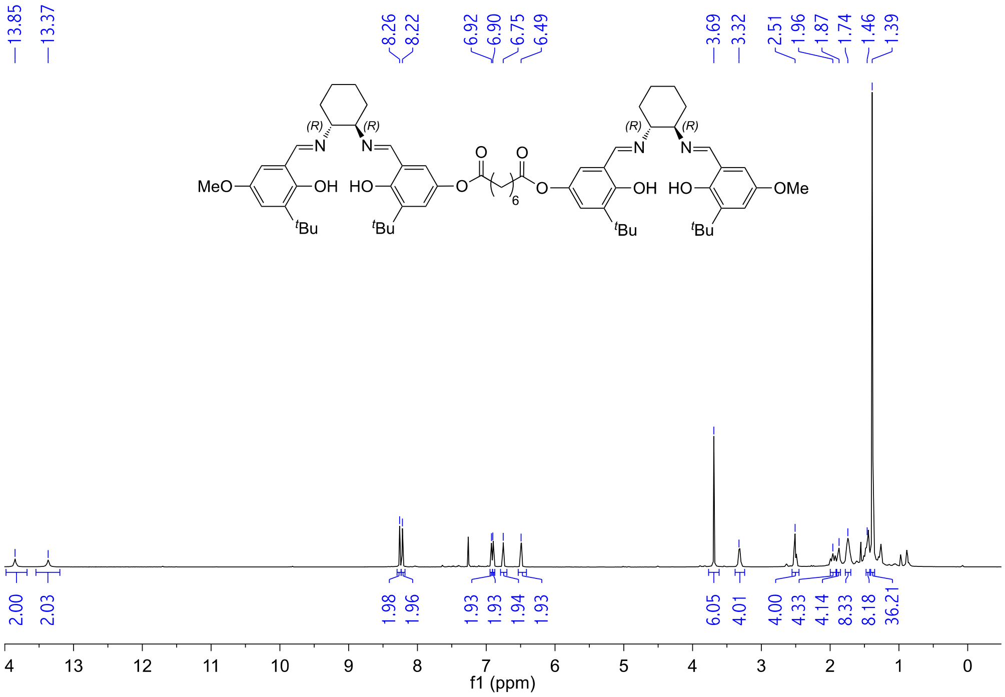

6-Ligand, ^{13}C NMR spectrum (125 MHz, $CDCl_3$)

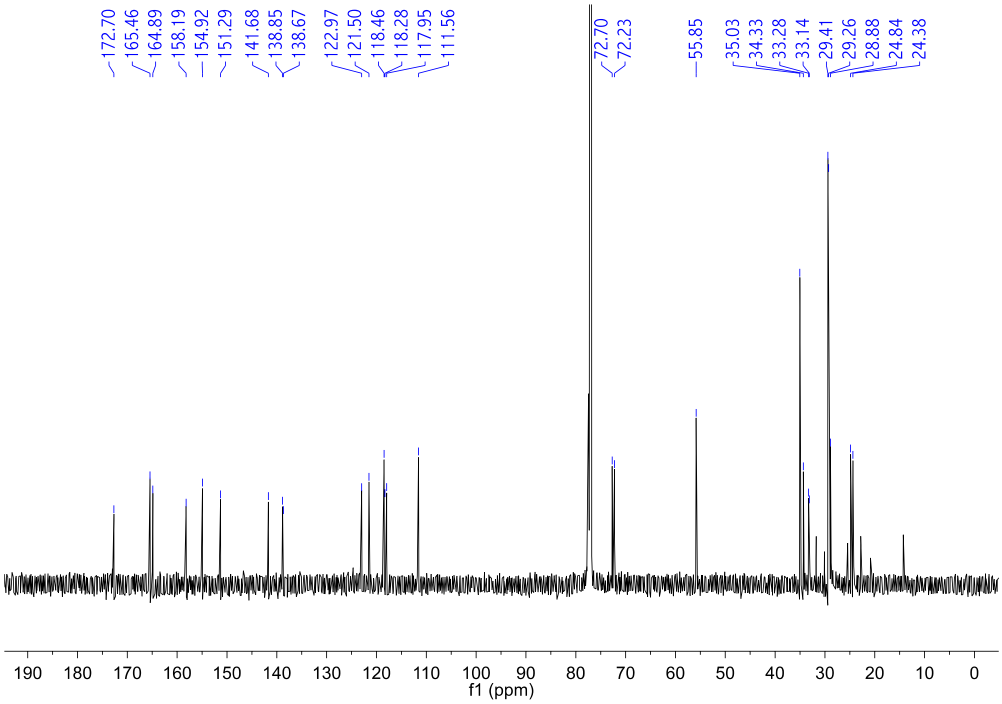

7-Ligand, ^{1}H NMR spectrum (500 MHz, $CDCl_3$)

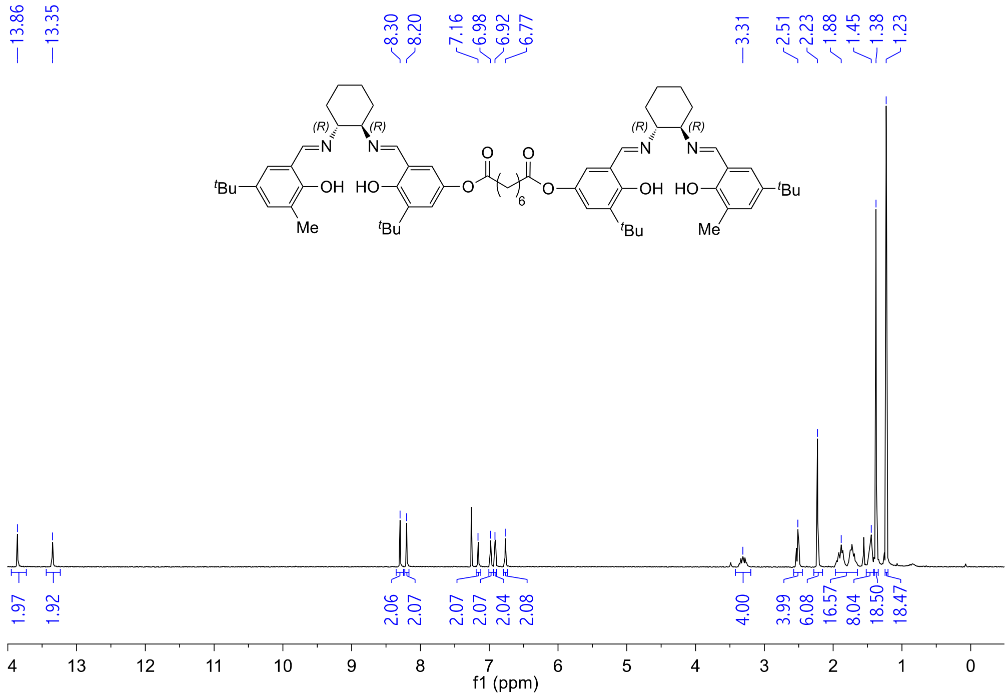

7-Ligand, ^{13}C NMR spectrum (125 MHz, $CDCl_3$)

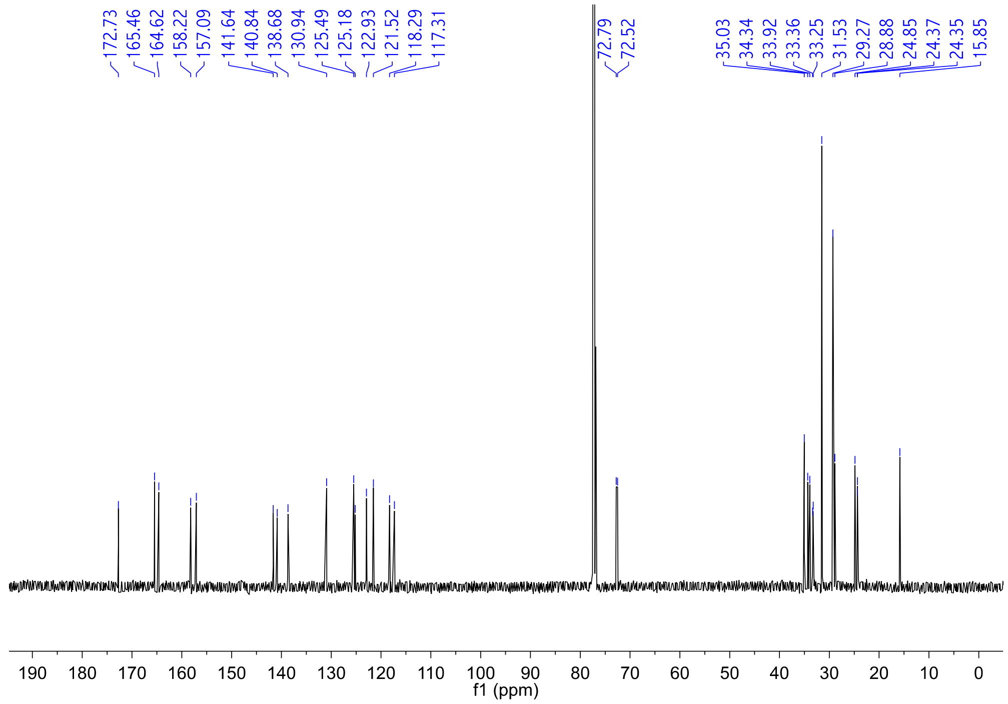

8-Ligand, ^{1}H NMR spectrum (500 MHz, $CDCl_3$)

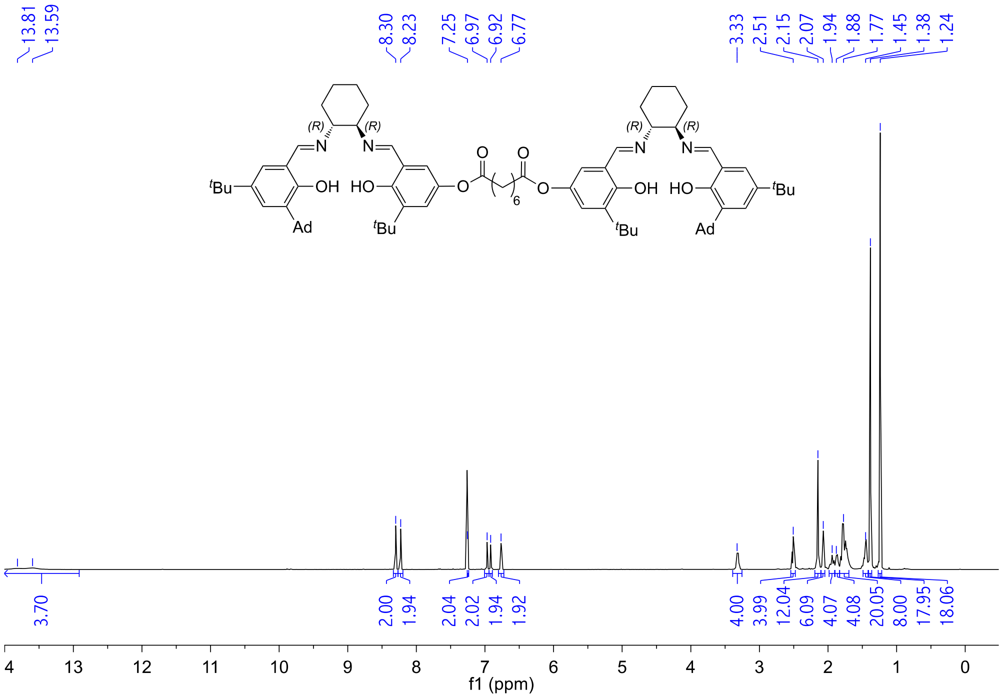

8-Ligand, ^{13}C NMR spectrum (125 MHz, $CDCl_3$)

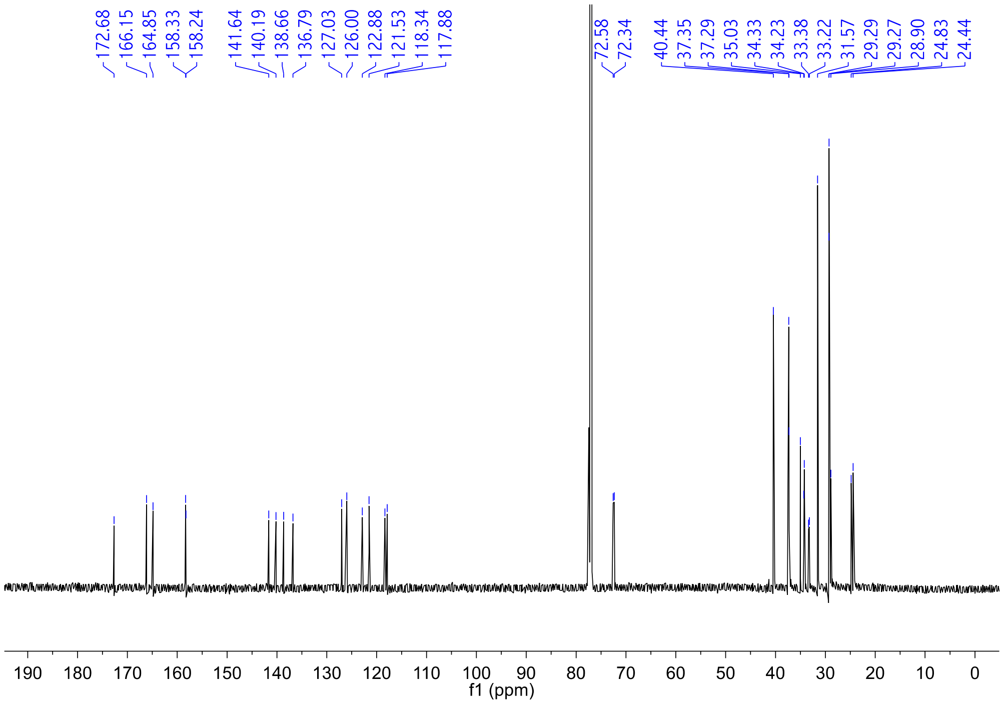

9-Ligand, ^{1}H NMR spectrum (500 MHz, $CDCl_3$)

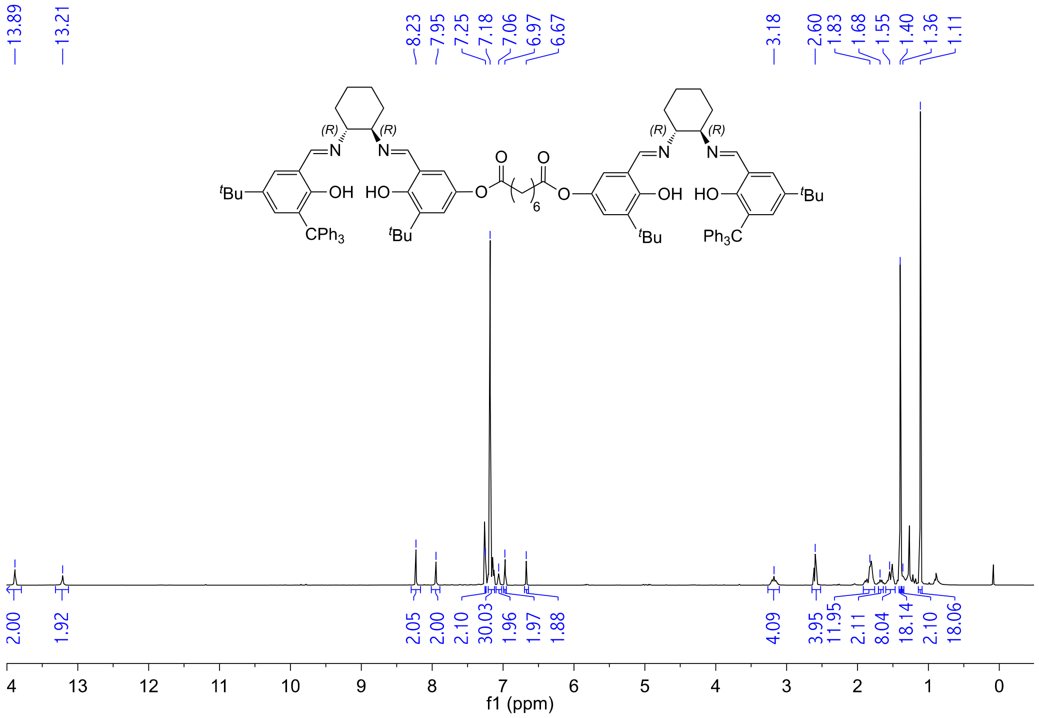

9-Ligand, ^{13}C NMR spectrum (125 MHz, $CDCl_3$)

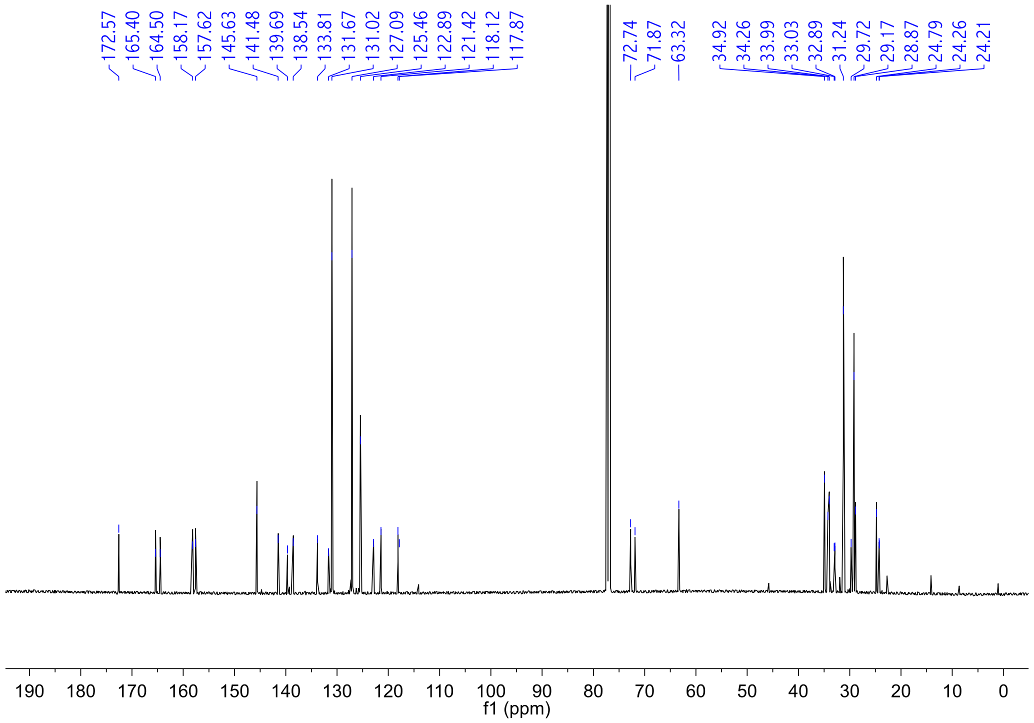

10-Ligand, ^{1}H NMR spectrum (500 MHz, $CDCl_3$)

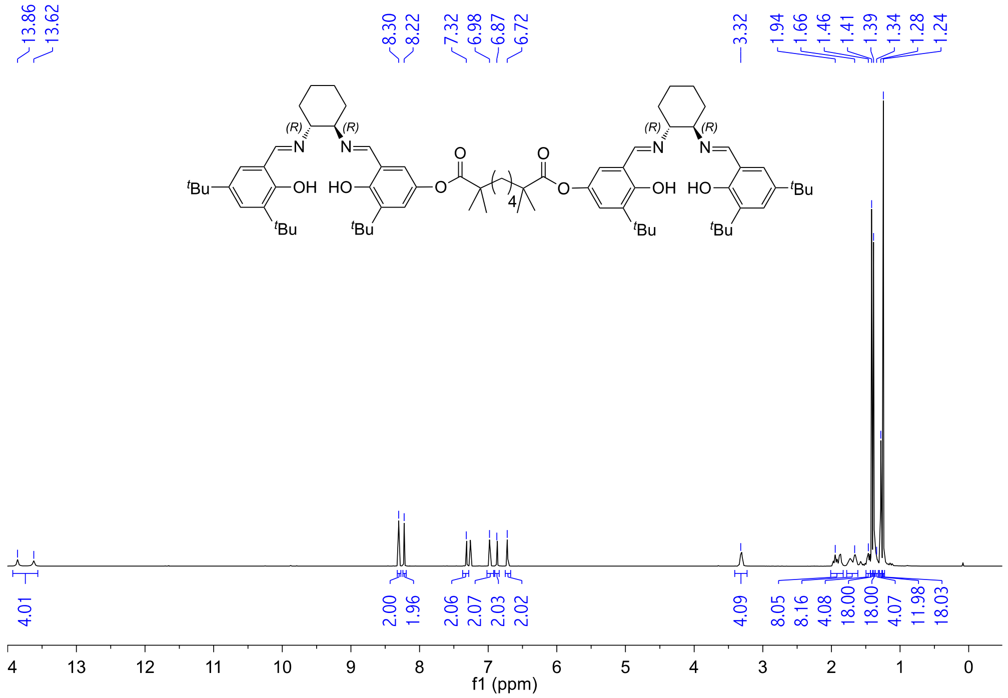

10-Ligand, ^{13}C NMR spectrum (125 MHz, $CDCl_3$)

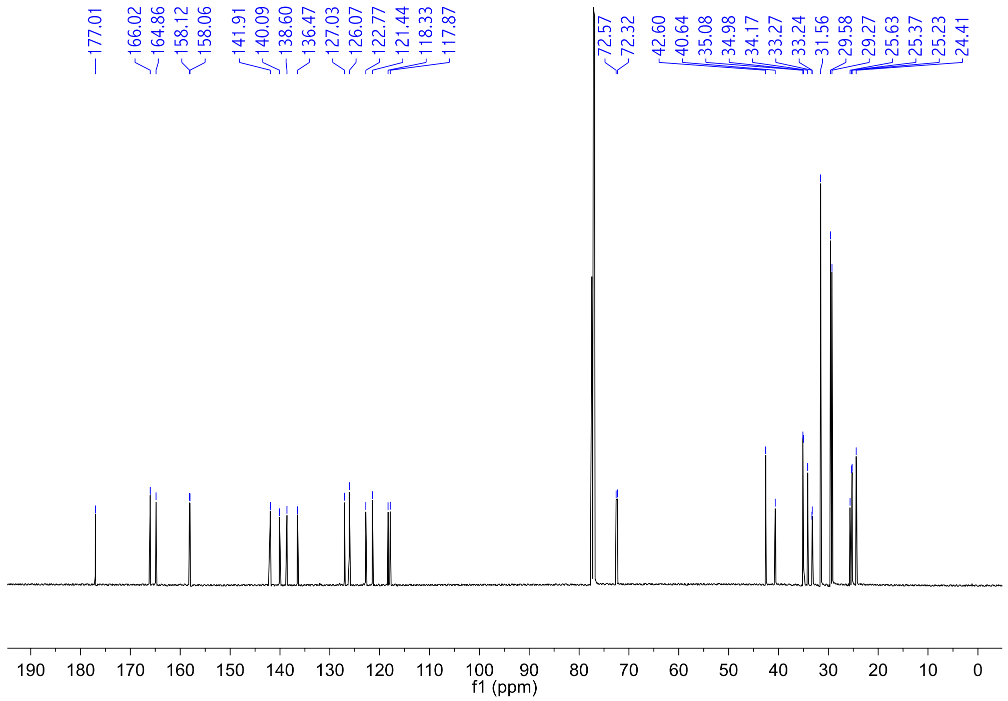

11-Ligand, ^{1}H NMR spectrum (500 MHz, $CDCl_3$)

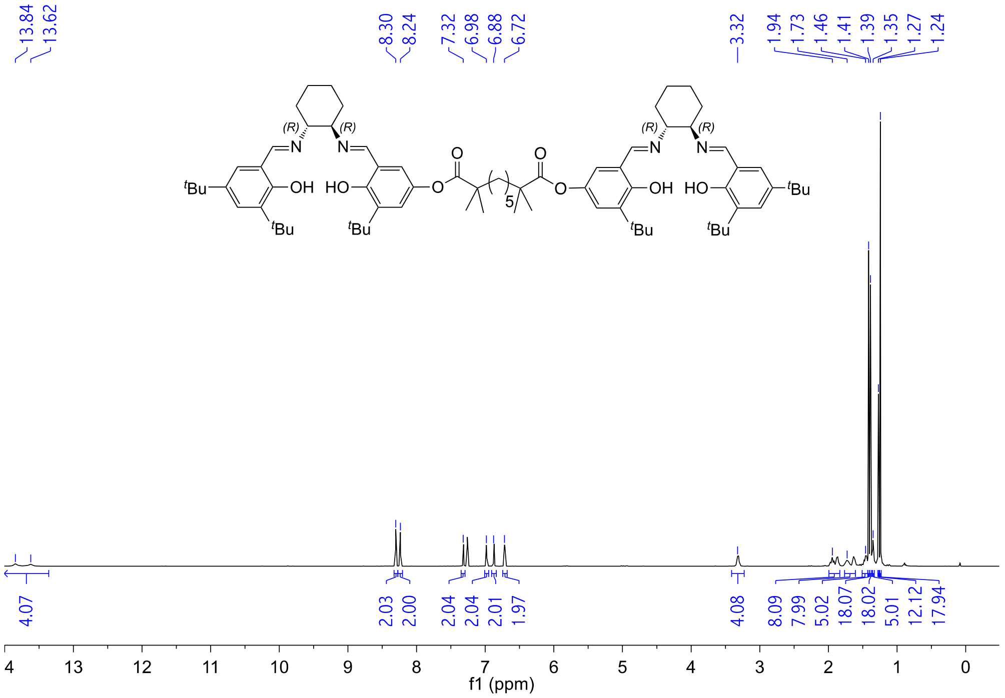

11-Ligand, ^{13}C NMR spectrum (125 MHz, $CDCl_3$)

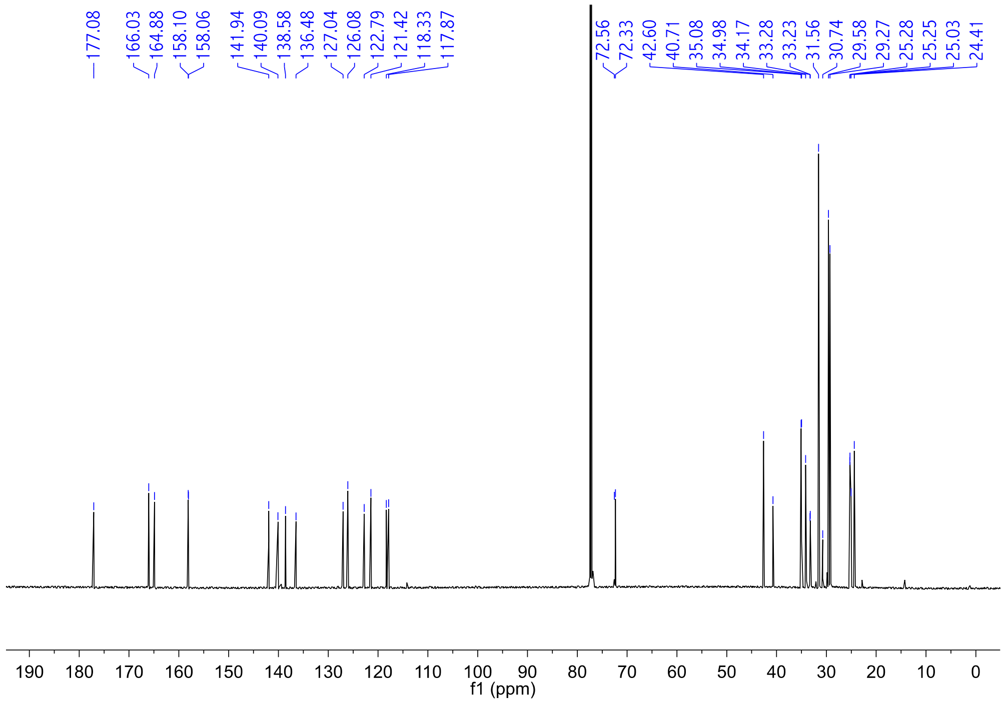

12-Ligand, ^{1}H NMR spectrum (500 MHz, $CDCl_3$)

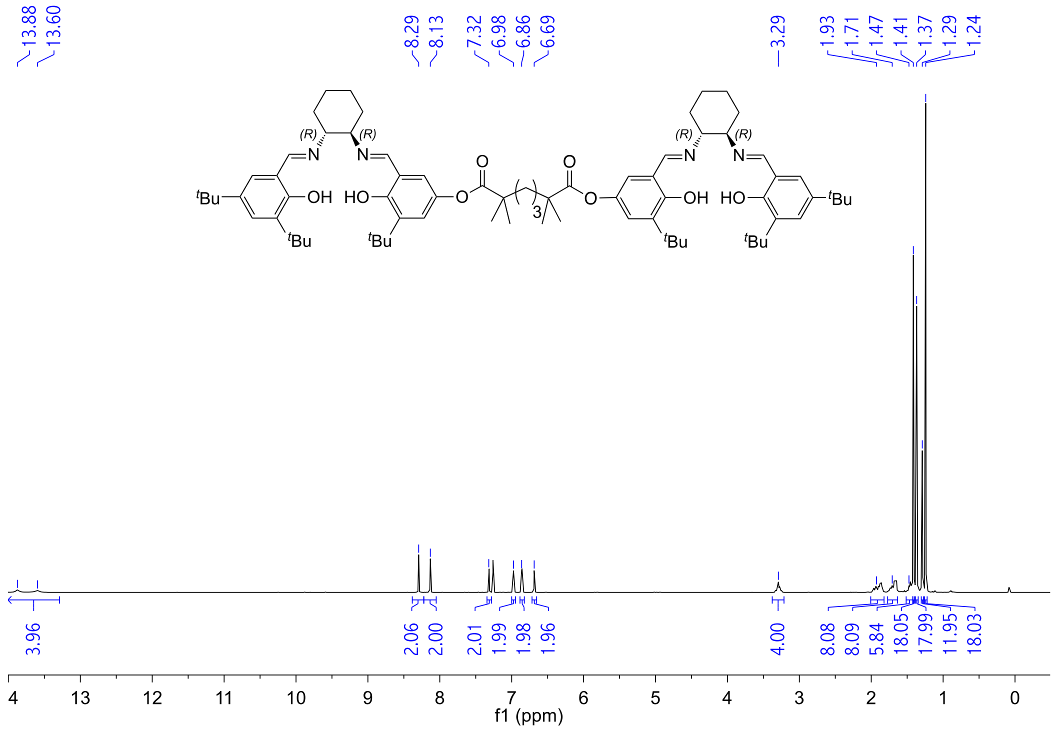

12-Ligand, ^{13}C NMR spectrum (125 MHz, $CDCl_3$)

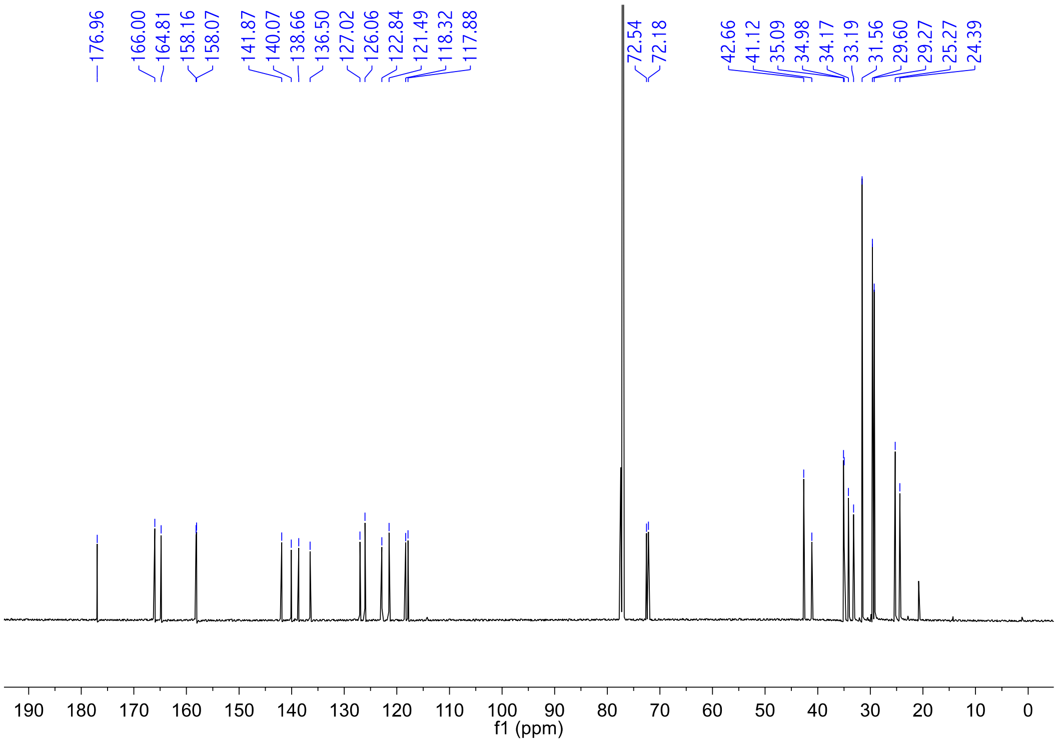

13-Ligand, ^{1}H NMR spectrum (500 MHz, $CDCl_3$)

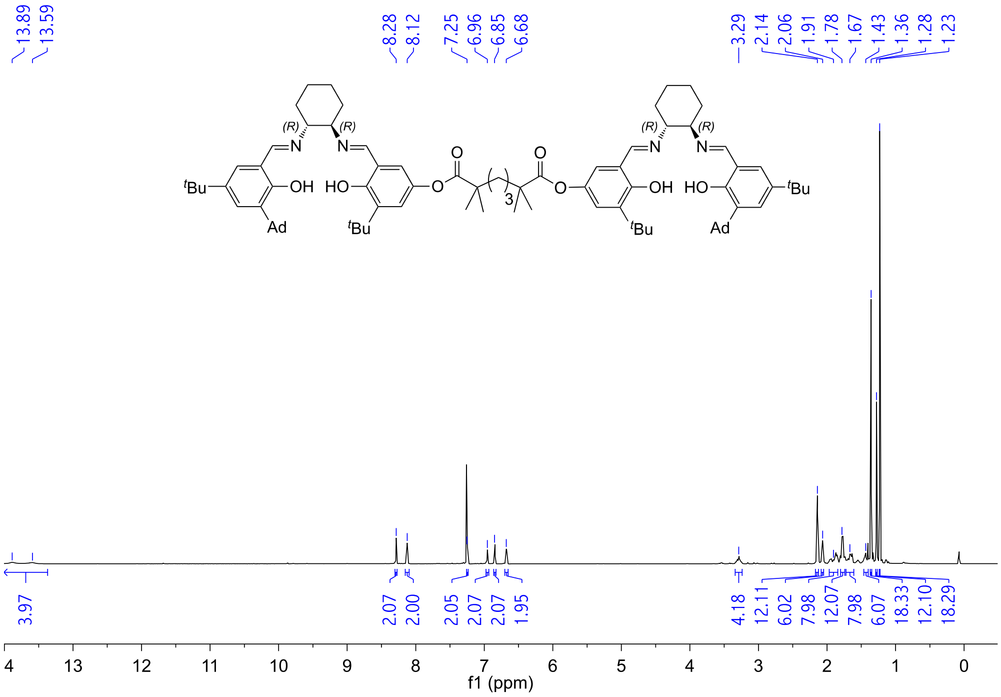

13-Ligand, ^{13}C NMR spectrum (125 MHz, $CDCl_3$)

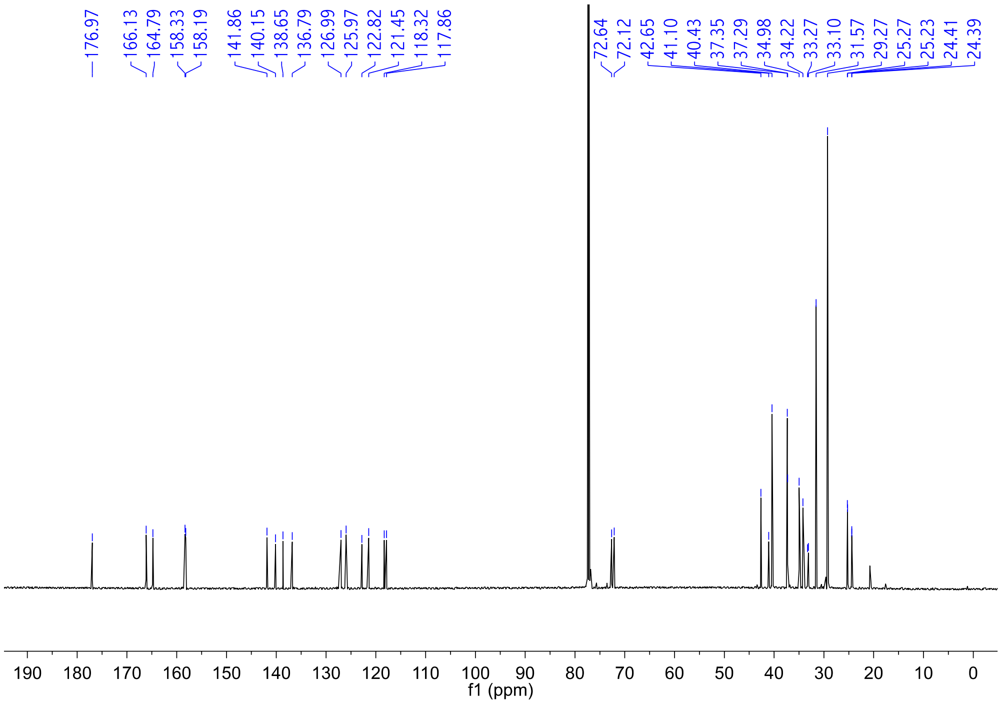

9. Reference

(1) (a) Frisch, M. J.; Trucks, G. W.; Schlegel, H. B.; Scuseria, G. E.; Robb, M. A.; Cheeseman, J. R.; Scalmani, G.; Barone, V.; Petersson, G. A.; Nakatsuji, H.; Li, X.; Caricato, M.; Marenich, A. V.; Bloino, J.; Janesko, B. G.; Gomperts, R.; Mennucci, B.; Hratchian, H. P.; Ortiz, J. V.; Izmaylov, A. F.; Sonnenberg, J. L.; Williams; Ding, F.; Lipparini, F.; Egidi, F.; Goings, J.; Peng, B.; Petrone, A.; Henderson, T.; Ranasinghe, D.; Zakrzewski, V. G.; Gao, J.; Rega, N.; Zheng, G.; Liang, W.; Hada, M.; Ehara, M.; Toyota, K.; Fukuda, R.; Hasegawa, J.; Ishida, M.; Nakajima, T.; Honda, Y.; Kitao, O.; Nakai, H.; Vreven, T.; Throssell, K.; Montgomery Jr., J. A.; Peralta, J. E.; Ogliaro, F.; Bearpark, M. J.; Heyd, J. J.; Brothers, E. N.; Kudin, K. N.; Staroverov, V. N.; Keith, T. A.; Kobayashi, R.; Normand, J.; Raghavachari, K.; Rendell, A. P.; Burant, J. C.; Iyengar, S. S.; Tomasi, J.; Cossi, M.; Millam, J. M.; Klene, M.; Adamo, C.; Cammi, R.; Ochterski, J. W.; Martin, R. L.; Morokuma, K.; Farkas, O.; Foresman, J. B.; Fox, D. J. Gaussian 16 Rev. B.01, Wallingford, CT, 2016. (b) Ahmed S. M., Poater A.; Childers M. I.; Widger P. C. B.; LaPointe A. M.; Lobkovsky E. B.; Coates G. W.; Cavallo L. Enantioselective Polymerization of Epoxides Using Biaryl-linked Bimetallic Cobalt Catalysts: A Mechanistic Study. *J. Am. Chem. Soc.* **2013**, *135*, 18901–18911.

(2) Lipinski, B. M.; Morris, L. S.; Silberstein, M. N.; Coates, G. W. Isotactic Poly(Propylene Oxide): A Photodegradable Polymer with Strain Hardening Properties. *J. Am. Chem. Soc.* **2020**, *142*, 6800–6806.

(3) Campbell, E. J.; Nguyen, S. T. Unsymmetrical Salen-Type Ligands: High Yield Synthesis of Salen-Type Schiff Bases Containing Two Different Benzaldehyde Moieties. *Tetrahedron Lett*. **2001**, *42*, 1221–1225.

(4) Hirahata, W.; Thomas, R. M.; Lobkovsky, E. B.; Coates, G. W. Enantioselective Polymerization of Epoxides: A Highly Active and Selective Catalyst for the Preparation of Stereoregular Polyethers and Enantiopure Epoxides. *J. Am. Chem. Soc*. **2008**, *130*, 17658–17659.

(5) Ten Cate, A. T.; Dankers, P. Y. W.; Kooijman, H.; Spek, A. L.; Sijbesma, R. P.; Meijer, E. W. Enantioselective Cyclization of Racemic Supramolecular Polymers. *J. Am. Chem. Soc.* **2003**, *125*, 6860–6861.

(6) Ten Cate, A. T.; Kooijman, H.; Spek, A. L.; Sijbesma, R. P.; Meijer, E. W. Conformational Control in the Cyclization of Hydrogen-Bonded Supramolecular Polymers. *J. Am. Chem. Soc.* **2004**, *126*, 3801–3808.

(7) Esguerra, K. V. N.; Fall, Y.; Petitjean, L.; Lumb, J.P. Controlling the Catalytic Aerobic Oxidation of Phenols. *J. Am. Chem. Soc.* **2014**, *136*, 7662–7668.

(8) Kurahashi, T.; Fujii, H. One-Electron Oxidation of Electronically Diverse Manganese(III) and Nickel(II) Salen Complexes: Transition from Localized to Delocalized Mixed-Valence Ligand Radicals. *J. Am. Chem. Soc*. **2011**, *133*, 8307–8316.

(9) Gao, Z.; Lim, Y. H.; Tredwell, M.; Li, L.; Verhoog, S.; Hopkinson, M.; Kaluza, W.; Collier, T. L.; Passchier, J.; Huiban, M.; Gouverneur, V. Metal-free Oxidative Fluorination of Phenols With [18F]fluoride. *Angew. Chem. Int. Ed.* **2012**, *51*, 6733–6737.

(10) Ay, S.; Nieger, M.; Bräse, S. Co-Metal-Free Enantioselective Conjugate Addition Reactions of Zinc Reagents. *Chem. Eur. J.* **2008**, *14*, 11539–11556.

(11) Green, G. D.; Swedo, R.; Butterick, R. US Patent WO2014/008164A1, 2014.

(12) White, D. E.; Tadross, P. M.; Lu, Z.; Jacobsen, E. N. A Broadly Applicable and Practical Oligomeric (Salen)Co Catalyst for Enantioselective Epoxide Ring-Opening Reactions. *Tetrahedron* **2014**, *70*, 4165–4180.

(13) DiCiccio, A. M.; Longo, J. M.; Rodríguez-Calero, G. G.; Coates, G. W. Development of Highly Active and Regioselective Catalysts for the Copolymerization of Epoxides with Cyclic Anhydrides: An Unanticipated Effect of Electronic Variation. *J. Am. Chem. Soc.* **2016**, *138*, 7107–7113.

(14) Pietikäinen, P. Asymmetric Epoxidation of Unfunctionalized Alkenes with Ammonium and Phosphonium Monopersulfates Catalyzed by Chiral Mn(III)–Salen Complexes. *Tetrahedron* **2000**, *56*, 417–424.

(15) Van Zee, N. J.; Sanford, M. J.; Coates, G. W. Electronic Effects of Aluminum Complexes in the Copolymerization of Propylene Oxide with Tricyclic Anhydrides: Access to Well-Defined, Functionalizable Aliphatic Polyesters. *J. Am. Chem. Soc.* **2016**, *138*, 2755–2761.

(16) Rudzevich, V.; Schollmeyer, D.; Braekers, D.; Desreux, J. F.; Diss, R.; Wipff, G.; Böhmer, V. Carbamoylmethylphosphinoxide Derivatives Based on the Triphenylmethane Skeleton. Synthesis and Extraction Properties. *J. Org. Chem.* **2005**, *70*, 6027–6033.

(17) Kochnev, A. I.; Oleynik, I. I.; Oleynik, I. V.; Ivanchev, S. S.; Tolstikov, G. A. Synthesis of Salicylaldehydes Bearing Bulky Substituents in the Positions 3 and 5. *Russ. Chem. Bull.* **2007**, 56, 1125–1129.

(18) Morris, L. S.; Childers, M. I.; Coates, G. W. Bimetallic Chromium Catalysts with Chain Transfer Agents: A Route to Isotactic Poly(propylene oxide)s with Narrow Dispersities. *Angew. Chem. Int. Ed.* **2018**, *57*, 5731–5734.
